# Supplementary material for: High-resolution single-cell atlas reveals diversity and plasticity of tissue-resident neutrophils in non-small cell lung cancer
Source: Cancer Cell. 2022 Dec 12;40(12):1503–1520.e8. doi: 10.1016/j.ccell.2022.10.008 (PMC9767679; doi:10.1016/j.ccell.2022.10.008)
Supplement: Document S1. Figures S1–S7 and Tables S2–S4, S7, and S8 [file mmc1.pdf]

Cancer Cell, Volume *40*

Supplemental information

High-resolution single-cell atlas reveals diversity and plasticity of tissue-resident neutrophils in non-small cell lung cancer

Stefan Salcher, Gregor Sturm, Lena Horvath, Gerold Untergasser, Christiane Kuempers, Georgios Fotakis, Elisa Panizzolo, Agnieszka Martowicz, Manuel Trebo, Georg Pall, Gabriele Gamerith, Martina Sykora, Florian Augustin, Katja Schmitz, Francesca Finotello, Dietmar Rieder, Sven Perner, Sieghart Sopper, Dominik Wolf, Andreas Pircher, and Zlatko Trajanoski

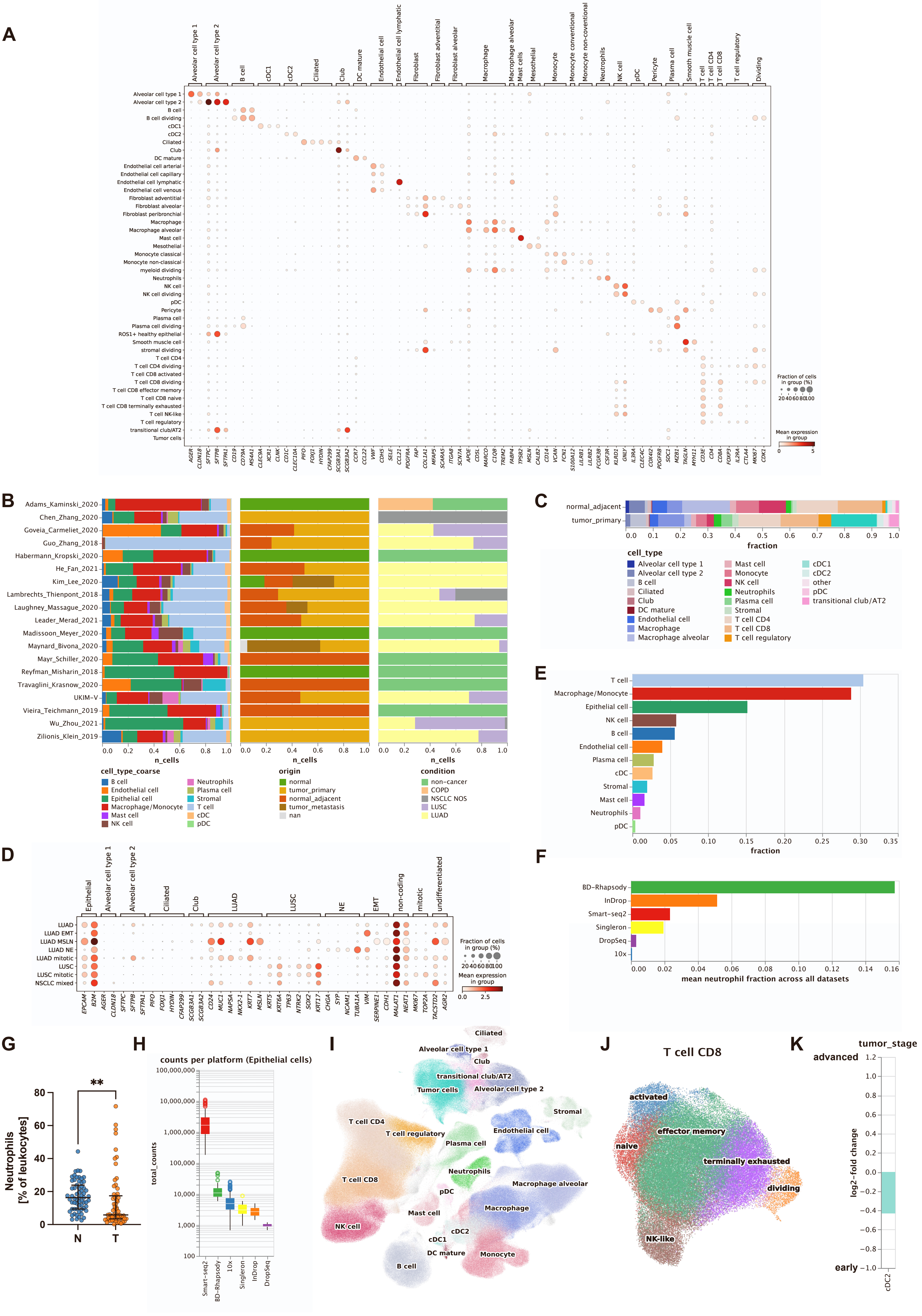
A
Alveolar cell type 1
Alveolar cell type 2
B cell
cDC1
cDC2
Ciliated
Club
DC mature
Endothelial cell
Endothelial cell lymphatic
Fibroblast
Fibroblast adventitial
Fibroblast alveolar
Macrophage
Macrophage alveolar
Mast cells
Mesothelial
Monocyte
Monocyte conventional
Monocyte non-coventional
Neutrophils
NK cell
pDC
Pericyte
Plasma cell
Smooth muscle cell
T cell
T cell CD4
T cell CD8
T cell regulatory
Dividing
Alveolar cell type 1
Alveolar cell type 2
B cell
B cell dividing
cDC1
cDC2
Ciliated
Club
DC mature
Endothelial cell arterial
Endothelial cell capillary
Endothelial cell lymphatic
Endothelial cell venous
Fibroblast adventitial
Fibroblast alveolar
Fibroblast peribronchial
Macrophage
Macrophage alveolar
Mast cell
Mesothelial
Monocyte classical
Monocyte non-classical
myeloid dividing
Neutrophils
NK cell
NK cell dividing
pDC
Pericyte
Plasma cell
Plasma cell dividing
ROS1+ healthy epithelial
Smooth muscle cell
stromal dividing
T cell CD4
T cell CD4 dividing
T cell CD8 activated
T cell CD8 dividing
T cell CD8 effector memory
T cell CD8 naive
T cell CD8 terminally exhausted
T cell NK-like
T cell regulatory
transitional club/AT2
Tumor cells
AGER CLDN18 SFTPC SFTPB SFTPA1 CD19 CD79A MS4A1 CLEC9A XCR1 CLNK CD1C CLEC10A PIFO FOXJ1 HYDIN CFAP299 SCGB3A1 SCGB3A2 CCR7 CCL22 VWF CDH5 SELE CCL21 PDGFRA FAP COL1A1 MFAP5 SCARA5 ITGA8 SCN7A APOE CD5L MARCO C1QB TREM2 FABP4 TPSB2 MSLN CALB2 CD14 VCAN FCN1 S100A12 LILRB1 LILRB2 FCGR3B CSF3R KLRD1 GNLY IL3RA CLEC4C COX4I2 PDGFRB SDC1 MZB1 TAGLN MYH11 CD3E CD4 CD8A FOXP3 IL2RA CTLA4 MKI67 CDK1
Fraction of cells in group (%)
20 40 60 80 100
Mean expression in group
0 5
B
Adams_Kaminski_2020
Chen_Zhang_2020
Goveia_Carmeliet_2020
Guo_Zhang_2018
Habermann_Kropski_2020
He_Fan_2021
Kim_Lee_2020
Lambrechts_Thienpont_2018
Laughney_Massague_2020
Leader_Merad_2021
Madissoon_Meyer_2020
Maynard_Bivona_2020
Mayr_Schiller_2020
Reyfman_Misharin_2018
Travaglini_Krasnow_2020
UKIM-V
Vieira_Teichmann_2019
Wu_Zhou_2021
Zilionis_Klein_2019
0.0 0.2 0.4 0.6 0.8 1.0
n_cells
cell_type_coarse
B cell
Endothelial cell
Epithelial cell
Macrophage/Monocyte
Mast cell
NK cell
Neutrophils
Plasma cell
Stromal
T cell
cDC
pDC
origin
normal
tumor_primary
normal_adjacent
tumor_metastasis
nan
condition
non−cancer
COPD
NSCLC NOS
LUSC
LUAD
C
normal_adjacent
tumor_primary
0.0 0.1 0.2 0.3 0.4 0.5 0.6 0.7 0.8 0.9 1.0
fraction
cell_type
Alveolar cell type 1
Alveolar cell type 2
B cell
Ciliated
Club
DC mature
Endothelial cell
Macrophage
Macrophage alveolar
Mast cell
Monocyte
NK cell
Neutrophils
Plasma cell
Stromal
T cell CD4
T cell CD8
T cell regulatory
cDC1
cDC2
other
pDC
transitional club/AT2
E
T cell
Macrophage/Monocyte
Epithelial cell
NK cell
B cell
Endothelial cell
Plasma cell
cDC
Stromal
Mast cell
Neutrophils
pDC
0.00 0.05 0.10 0.15 0.20 0.25 0.30 0.35
fraction
D
Epithelial
Alveolar cell type 1
Alveolar cell type 2
Ciliated
Club
LUAD
LUSC
NE
EMT
non-coding
mitotic
undifferentiated
LUAD
LUAD EMT
LUAD MSLN
LUAD NE
LUAD mitotic
LUSC
LUSC mitotic
NSCLC mixed
EPCAM B2M AGER CLDN18 SFTPC SFTPB SFTPA1 PIFO FOXJ1 HYDIN CFAP299 SCGB3A1 SCGB3A2 CD24 MUC1 NAPSA NKX2-1 KRT7 MSLN KRT5 KRT6A TP63 NTRK2 SOX2 KRT17 CHGA SYP NCAM1 TUBA1A VIM SERPINE1 CDH1 MALAT1 NEAT1 MKI67 TOP2A TACSTD2 AGR2
Fraction of cells in group (%)
20 40 60 80 100
Mean expression in group
0.0 2.5
F
BD−Rhapsody
InDrop
Smart−seq2
Singleron
DropSeq
10x
0.00 0.02 0.04 0.06 0.08 0.10 0.12 0.14 0.16
mean neutrophil fraction across all datasets
G
Neutrophils [% of leukocytes]
**
0 20 40 60 80
N T
H
counts per platform (Epithelial cells)
100,000,000
10,000,000
1,000,000
100,000
10,000
1,000
100
total_counts
Smart−seq2 BD−Rhapsody 10x Singleron InDrop DropSeq
I
Ciliated
Alveolar cell type 1
Club
transitional club/AT2
Tumor cells
Alveolar cell type 2
Stromal
T cell CD4
T cell regulatory
Plasma cell
Endothelial cell
Neutrophils
T cell CD8
Macrophage alveolar
pDC
Macrophage
Mast cell
NK cell
cDC2
cDC1
DC mature
Monocyte
B cell
J
T cell CD8
activated
effector memory
naive
terminally exhausted
dividing
NK-like
K
tumor_stage
advanced
early
log2−fold change
1.2 1.0 0.8 0.6 0.4 0.2 0.0 −0.2 −0.4 −0.6 −0.8 −1.0
cDC2

Figure S1: related to Figure 1. Composition of the NSCLC single-cell atlas.

(A) Dotplot of cell type marker genes used for cell-type annotation.

(B) Fractions of cell types, sample origins and conditions per study (extended atlas).

(C) Relative cell type proportions by tissue origin in the core atlas. The depicted fractions are the average of all patients, independent of their cell-count.

(D) Marker genes for cancer cell classification.

(E) Cell type fractions in the extended atlas.

(F) Mean neutrophil fraction per sequencing platform across all datasets.

(G) Flow-cytometry of neutrophils (shown as percentage of leucocytes) in tumor tissue and patient-matched normal-adjacent tissue (n=63; Paired Wilcoxon test, **$p<0.01$). The horizontal line represents the median, whiskers extend to the inter-quartile range.

(H) Number of reads (Smart-seq2) or UMIs (other platforms) in epithelial cells by sequencing platform. The central line denotes the median, boxes represent the interquartile range (IQR) and whiskers extend to the most extreme values within 1.5 * IQR. Points outside 1.5 * IQR are shown as outliers.

(I) UMAP of the extended atlas, colored by the medium resolution cell-type annotation used for most data analyses.

(J) UMAP of $CD8^+$ T cell subclusters according to gene signatures published by Oliveira et al.[22].

(K) Cell type composition by tumor stage (early, late) calculated with scCODA (Bayesian model for differential composition analysis) using cancer cells as reference cell-type (assumed to be constant between conditions), including tumor type (LUAD, LUSC) as a covariate and running 500,000 Markov-chain monte carlo iterations. FDR=0.1.

Table S2: related to Figure 1. Patient number per cell type.

	Number of patients with ≥ 5/ ≥10 / ≥ 30 cells		
**Cell type**	**5**	**10**	**30**
Alveolar cell type 1	133	113	69
Alveolar cell type 2	172	161	122
B cell	226	196	164
B cell dividing	26	16	6
cDC1	131	99	32
cDC2	256	231	172
Ciliated	175	140	87
Club	101	54	21
DC mature	133	79	21
Endothelial cell arterial	108	65	33
Endothelial cell capillary	118	93	54
Endothelial cell lymphatic	129	90	37
Endothelial cell venous	207	171	96
Fibroblast adventitial	123	80	33
Fibroblast alveolar	107	75	38
Fibroblast peribronchial	115	78	38
Macrophage	291	273	242
Macrophage alveolar	228	201	177
Mast cell	205	178	120
Mesothelial	31	18	2
Monocyte classical	276	257	222
Monocyte non-classical	149	120	74
Myeloid dividing	195	153	76
Neutrophils	60	48	35
NK cell	238	219	186
NK cell dividing	71	28	3
pDC	151	113	55
Pericyte	83	52	22
Plasma cell	218	186	131
Plasma cell dividing	44	26	6
ROS1+ healthy epithelial	38	29	16
Smooth muscle cell	85	48	25
Stromal dividing	14	9	3
T cell CD4	269	253	209
T cell CD4 dividing	141	99	32
T cell CD8 activated	144	122	76
T cell CD8 dividing	109	71	27
T cell CD8 effector memory	252	223	193
T cell CD8 naive	183	156	115
T cell CD8 terminally exhausted	166	131	89
T cell NK-like	192	163	125
T cell regulatory	216	192	150
Transitional club/AT2	193	159	106

	Number of patients with ≥ 5/ ≥10 / ≥ 30 cells		
**Cell type**	**5**	**10**	**30**
Tumor cells LUAD	225	192	136
Tumor cells LUAD EMT	43	26	13
Tumor cells LUAD mitotic	98	64	23
Tumor cells LUAD MSLN	6	3	1
Tumor cells LUAD NE	6	3	3
Tumor cells LUSC	86	62	36
Tumor cells LUSC mitotic	79	60	32
Tumor cells NSCLC mixed	24	19	12

Table S3: related to Figure 1. Sample disposition.

Figure	Dataset	Sample origin
1A	overview	
1B	core atlas	all
1C	extended atlas	all
1D	UKIM-V	all
1E	extended atlas	all
1F	extended atlas	primary tumor
1G	independent Lübeck cohort	
2A	extended atlas	primary tumor
2B	extended atlas (cancer cells)	primary tumor
2C	extended atlas (cancer cells)	primary tumor
3A	extended atlas	primary tumor
3B	extended atlas	primary tumor
4A-E	extended atlas	primary tumor
4F	TCGA LUAD/LUSC	
4G	extended atlas (CD8+ T cells)	primary tumor
5A-B	extended atlas (neutrophils)	all
5C	independent FACS cohort	
5D	extended atlas (neutrophils)	all
5E	extended atlas	primary tumor
5F	extended atlas (neutrophils)	all
5G	independent FACS cohort	
5H	representative imaging of UKIM-V patient	
6A-B	extended atlas (neutrophils)	all
6C	independent FACS cohort	
6D-E	UKIM-V (neutrophils)	all
6F	ligands: extended atlas (neutrophils); receptors: extended atlas	ligands: all; receptors: primary tumor
6G	extended atlas	all
6H	extended atlas (neutrophils)	all
6I-K	POPLAR/OAK cohort	
S1A	core atlas	all
S1B	extended atlas	all
S1C	extended atlas	primary tumor + adjacent normal
S1D	core atlas (cancer cells)	primary tumor
S1E	extended atlas	all
S1F	extended atlas	all
S1G	independent FACS cohort	
S1H	extended atlas	all
S1I	extended atlas	all
S1J	extended atlas (CD8+ T cells)	all
S1K	extended atlas	primary tumor
S2A	independent Lübeck cohort	

Figure	Dataset	Sample origin
S2B-C	independent FACS cohort	
S2D	extended atlas	primary tumor
S2E	extended atlas	primary tumor
S3A	extended atlas	primary tumor
S4A-B	extended atlas	primary tumor
S4C	independent IF cohort	
S4E-F	extended atlas	primary tumor
S4G-H	TCGA LUAD/LUSC	
S5A	extended atlas (neutrophils)	primary tumor
S5B	extended atlas (neutrophils)	all
S5C	representative imaging of UKIM-V patient	
S6A-E	extended atlas (neutrophils)	all
S6F	independent FACS cohort	
S6G-K	POPLAR/OAK cohort	
S7A	gating strategy	

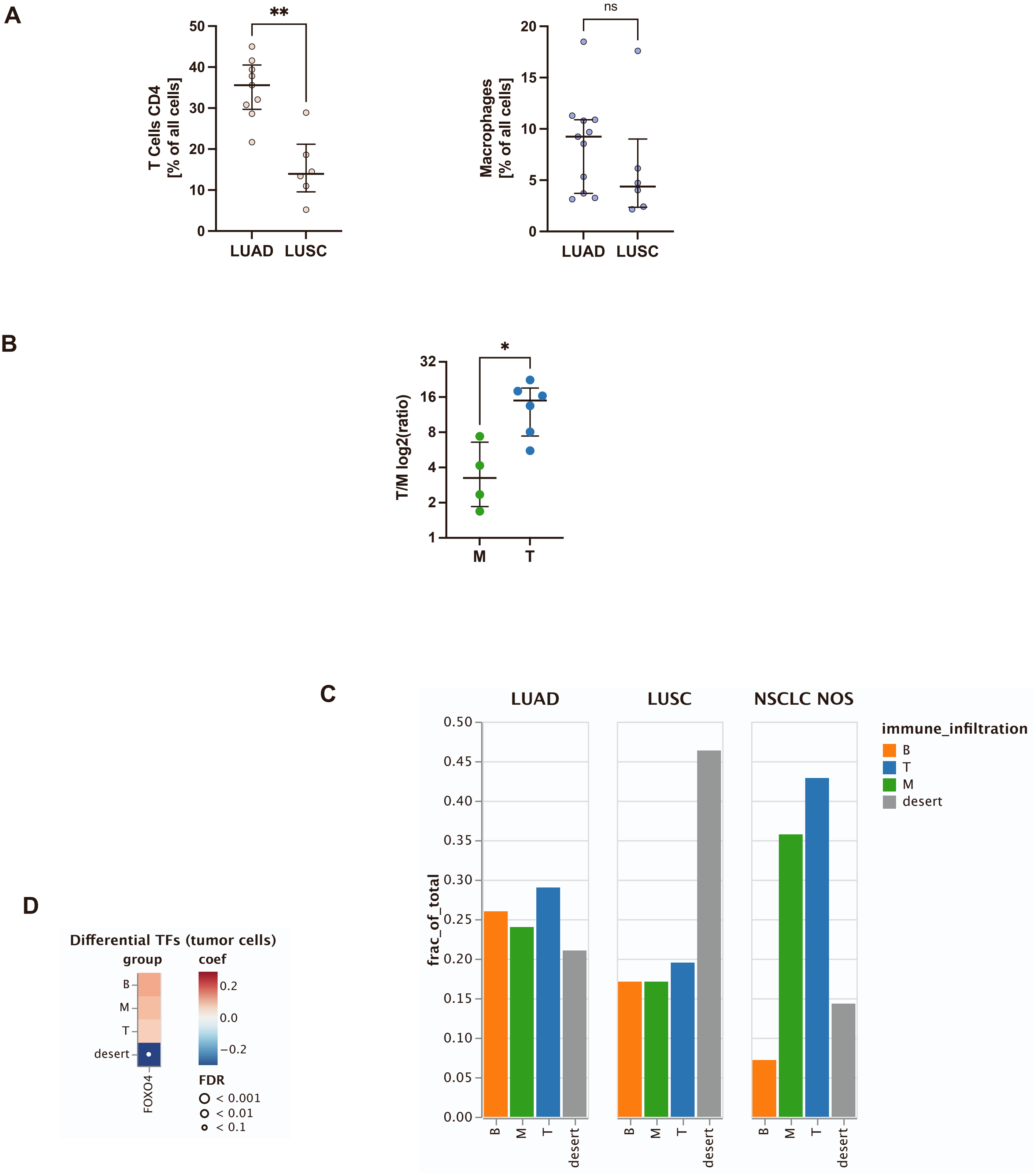
A
T Cells CD4
[% of all cells]
**
50
40
30
20
10
0
LUAD
LUSC
Macrophages
[% of all cells]
ns
20
15
10
5
0
LUAD
LUSC
B
T/M log2(ratio)
*
32
16
8
4
2
1
M
T
C
LUAD
LUSC
NSCLC NOS
frac_of_total
0.50
0.45
0.40
0.35
0.30
0.25
0.20
0.15
0.10
0.05
0.00
B
M
T
desert
immune_infiltration
B
T
M
desert
D
Differential TFs (tumor cells)
group
coef
B
M
T
desert
FOXO4
0.2
0.0
−0.2
FDR
< 0.001
< 0.01
< 0.1

Figure S2: related to Figure 2. Immune phenotypes in histological NSCLC subtypes.

(A) Flow cytometry analysis of $CD4^+$ lymphocytes and macrophages as percentage of all cells in LUAD (n=9) *versus* LUSC (n=6) tumor samples. The horizontal line represents the median, whiskers extend to the inter-quartile range (Wilcoxon test, **$p<0.01$).

(B) Flow cytometry analysis of the T cell-to myeloid cell ratio (T/M ratio) in tumor tissue (T subtype n=6, M subtype n=4). The horizontal line represents the median, whiskers extend to the inter-quartile range (Wilcoxon test, *$p<0.05$).

(C) Fractions of immune phenotypes in histological subtypes.

(D) Differential of DoRothEA transcription factor signatures in cancer cells between the four immune phenotypes. Heatmap colors indicate the deviation from the overall mean, independent of tumor histology and stage. White dots indicate significant interactions at different false-discovery-rate (FDR) thresholds. P-values have been calculated using a linear model f-test. Only transcription factors with an FDR < 0.1 in at least one patient group are shown.

A

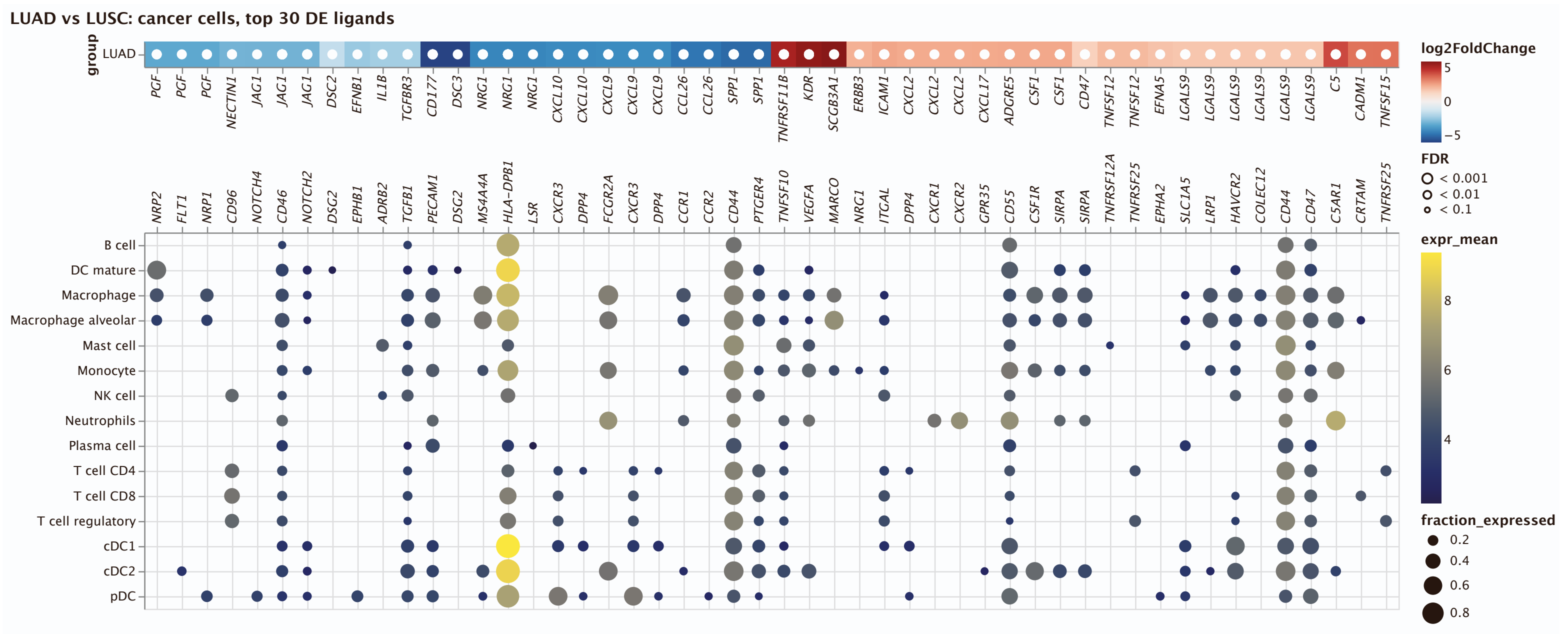

Figure S3: **related to Figure 3. Tumor-immune crosstalk in LUAD *vs.* LUSC,**

(A) Upper panel: top 30 differentially expressed ligands in LUAD *vs.* LUSC (DESeq2 on pseudo-bulk, FDR < 0.01). Heatmap colors indicate log2 fold changes clipped at ±5, where blue indicates upgregulation in LUSC and red indicates upregulation in LUAD. Bottom panel: Respective receptors and the expression by cell type. Dot sizes and colors refers to the fraction of cells expressing the receptor and gene expression, respectively, averaged over all patients. Dots are only shown for receptors that are expressed in at least 10% of the respective cell-types.

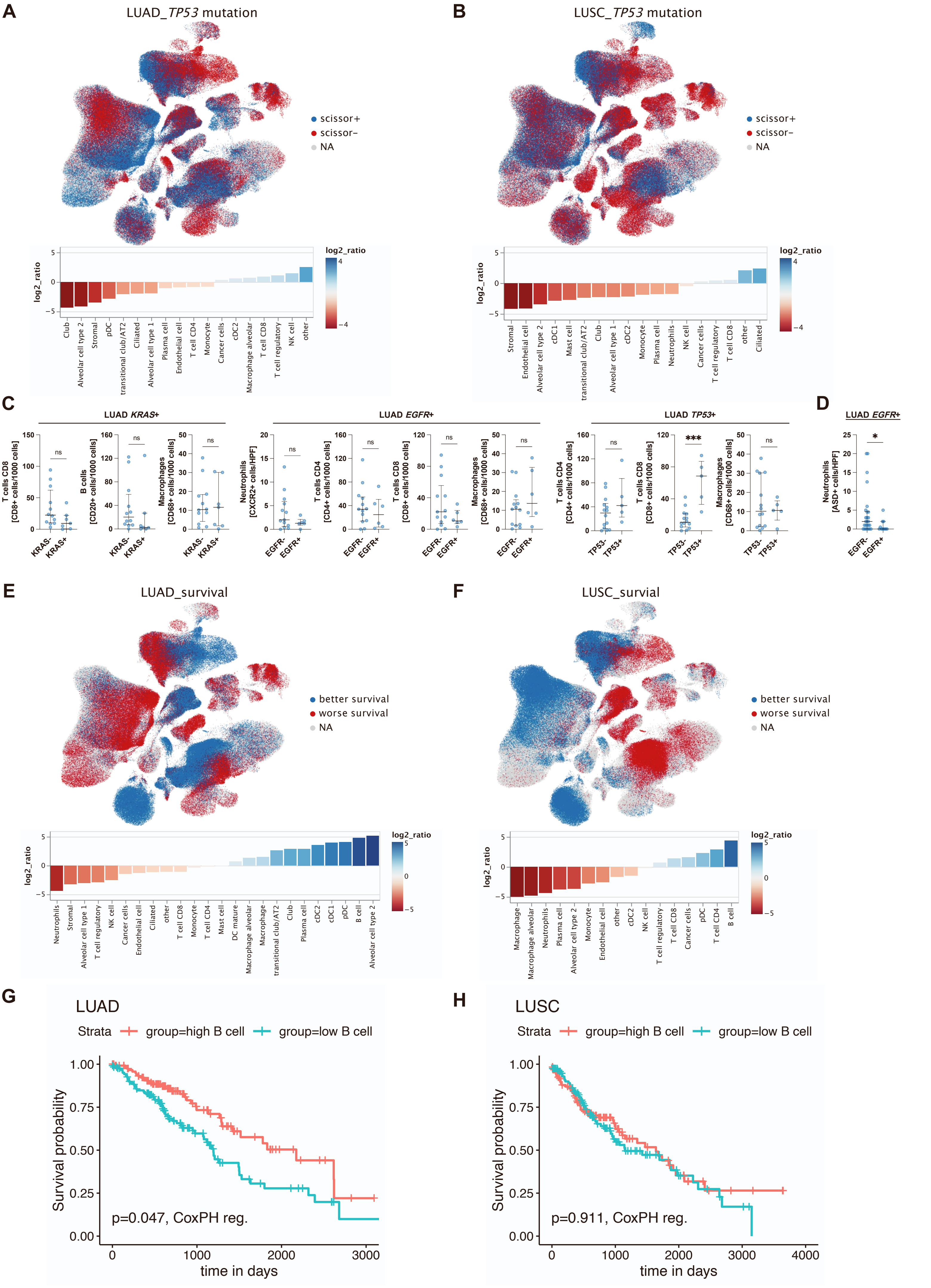
A
LUAD_TP53 mutation
scissor+
scissor−
NA
log2_ratio
Club
Alveolar cell type 2
Stromal
pDC
transitional club/AT2
Ciliated
Alveolar cell type 1
Plasma cell
Endothelial cell
T cell CD4
Monocyte
Cancer cells
cDC2
Macrophage alveolar
T cell CD8
T cell regulatory
NK cell
other
B
LUSC_TP53 mutation
scissor+
scissor−
NA
log2_ratio
Stromal
Endothelial cell
Alveolar cell type 2
cDC1
Mast cell
transitional club/AT2
Club
Alveolar cell type 1
cDC2
Monocyte
Plasma cell
Neutrophils
NK cell
Cancer cells
T cell regulatory
T cell CD8
other
Ciliated
C
LUAD KRAS+
LUAD EGFR+
LUAD TP53+
T cells CD8 [CD8+ cells/1000 cells]
B cells [CD20+ cells/1000 cells]
Macrophages [CD68+ cells/1000 cells]
Neutrophils [CXCR2+ cells/HPF]
T cells CD4 [CD4+ cells/1000 cells]
ns
***
KRAS-
KRAS+
EGFR-
EGFR+
TP53-
TP53+
D
LUAD EGFR+
Neutrophils [ASD+ cells/HPF]
*
EGFR-
EGFR+
E
LUAD_survival
better survival
worse survival
NA
log2_ratio
Neutrophils
Stromal
Alveolar cell type 1
T cell regulatory
NK cell
Cancer cells
Endothelial cell
Ciliated
other
T cell CD8
Monocyte
T cell CD4
Mast cell
DC mature
Macrophage alveolar
Macrophage
transitional club/AT2
Club
Plasma cell
cDC2
cDC1
pDC
B cell
Alveolar cell type 2
F
LUSC_survial
better survival
worse survival
NA
log2_ratio
Macrophage
Macrophage alveolar
Neutrophils
Plasma cell
Alveolar cell type 2
Monocyte
Endothelial cell
other
cDC2
NK cell
T cell regulatory
T cell CD8
Cancer cells
pDC
T cell CD4
B cell
G
LUAD
Strata
group=high B cell
group=low B cell
Survival probability
p=0.047, CoxPH reg.
time in days
H
LUSC
Strata
group=high B cell
group=low B cell
Survival probability
p=0.911, CoxPH reg.
time in days

Figure S4: related to Figure 4. Association of cellular composition and distinct genotypes and survival in the TCGA data,

(A-B) *SCISSOR* analysis showing the association of cellular composition and *TP53* mutation in LUAD and LUSC derived from the TCGA reference dataset.

(C) Staining of tumor tissue from LUAD patients with *KRAS* (n=7), *EGFR* (n=6) or *TP53* (n=5) mutation compared to LUAD tumor tissue negative for the respective mutation (n=12). Multiplex immunofluoresence was performed to detect $CD4^+$ T cells, $CD8^+$ T cells, $CD20^+$ B cells, or $CD68^+$ macrophages, respectively. Positive stained cells per 1000 cells are given. *CXCR2* expression was analyzed by immunohistochemistry and quantified per high power field in patients with *EGFR* mutation compared to EGFR wt patients. Analyses related to the corresponding *SCISSOR* analyses are shown. The horizontal line represents the median, whiskers extend to the inter-quartile range (Wilcoxon test, ***$p<0.001$).

(D) Immunohistochemistry staining of neutrophils (ASD^+ cells) quantified per high power field in patients with *EGFR* mutation (n=11) compared to *EGFR* wt patients (n=26). The horizontal line represents the median, whiskers extend to the inter-quartile range (Wilcoxon test, *$p<0.05$).

(E-F) Association of cellular composition with overall survival for LUSC and LUAD patients.

(G-H) Kaplan-Meyer plot of LUAD and LUSC patients with high (top 25%) and low (bottom 25%) B cell fractions of TCGA lung cancer patients as determined by deconvolution with EPIC. P-value has been determined using CoxPH-regression using tumor stage and age as covariates.

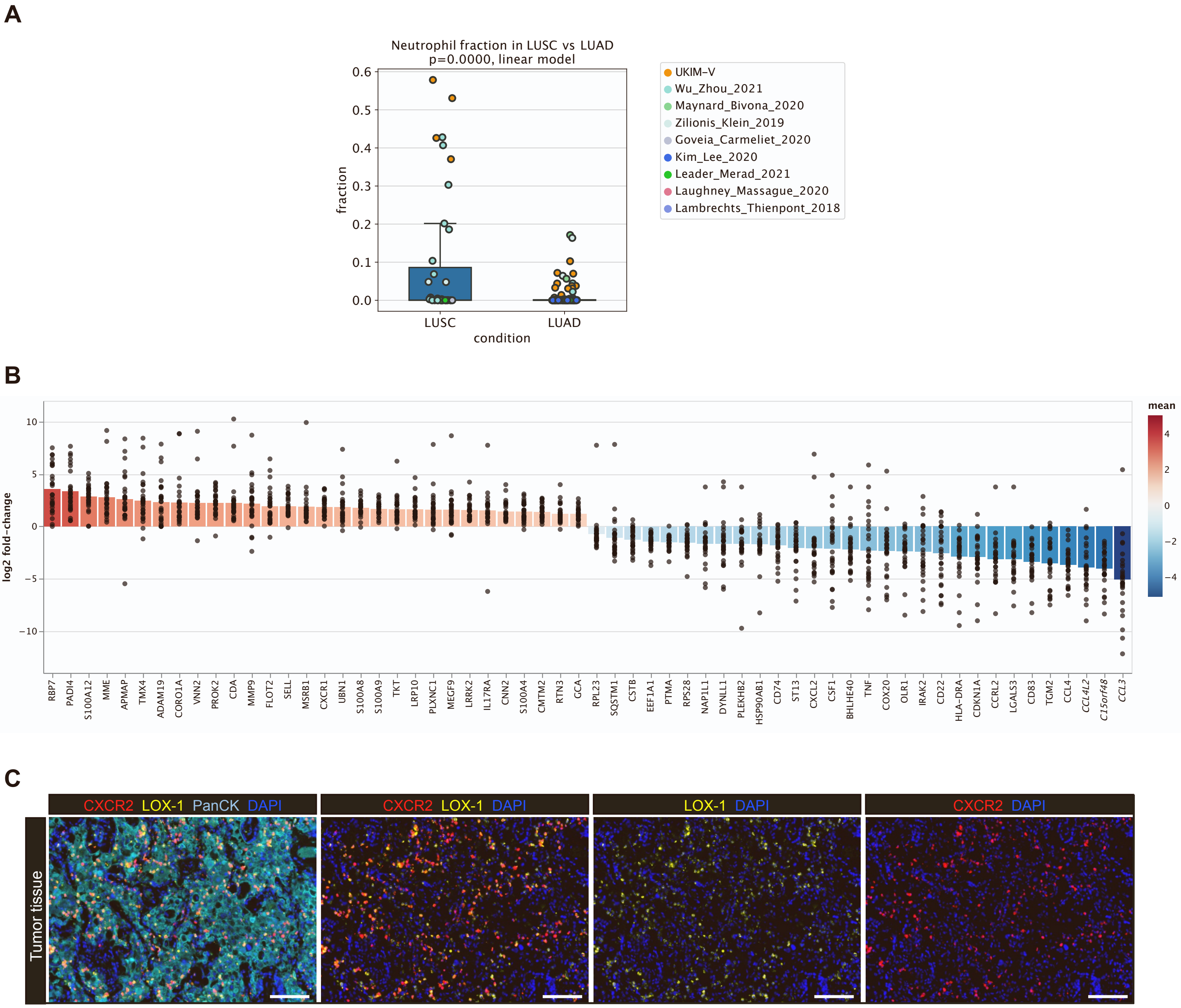

Figure S5: related to Figure 5. Characterization of tissue-resident neutrophils.

(A) Neutrophil fraction in LUSC *vs.* LUAD (extended atlas). P-value derived using linear model f-test including dataset as a covariate.

(B) Expression of top 30 marker genes (AUROC > 0.75) for NANs and TANs. Every dot is the log2 fold change on a single patient. Bars show the average log2-fold change.

(C) Multiplex immunofluorescence co-staining of CXCR2 (red), LOX-1 (yellow) and pan-cytokeratin (blue) in LUSC tumor tissue. Scale bar = 100 µm.

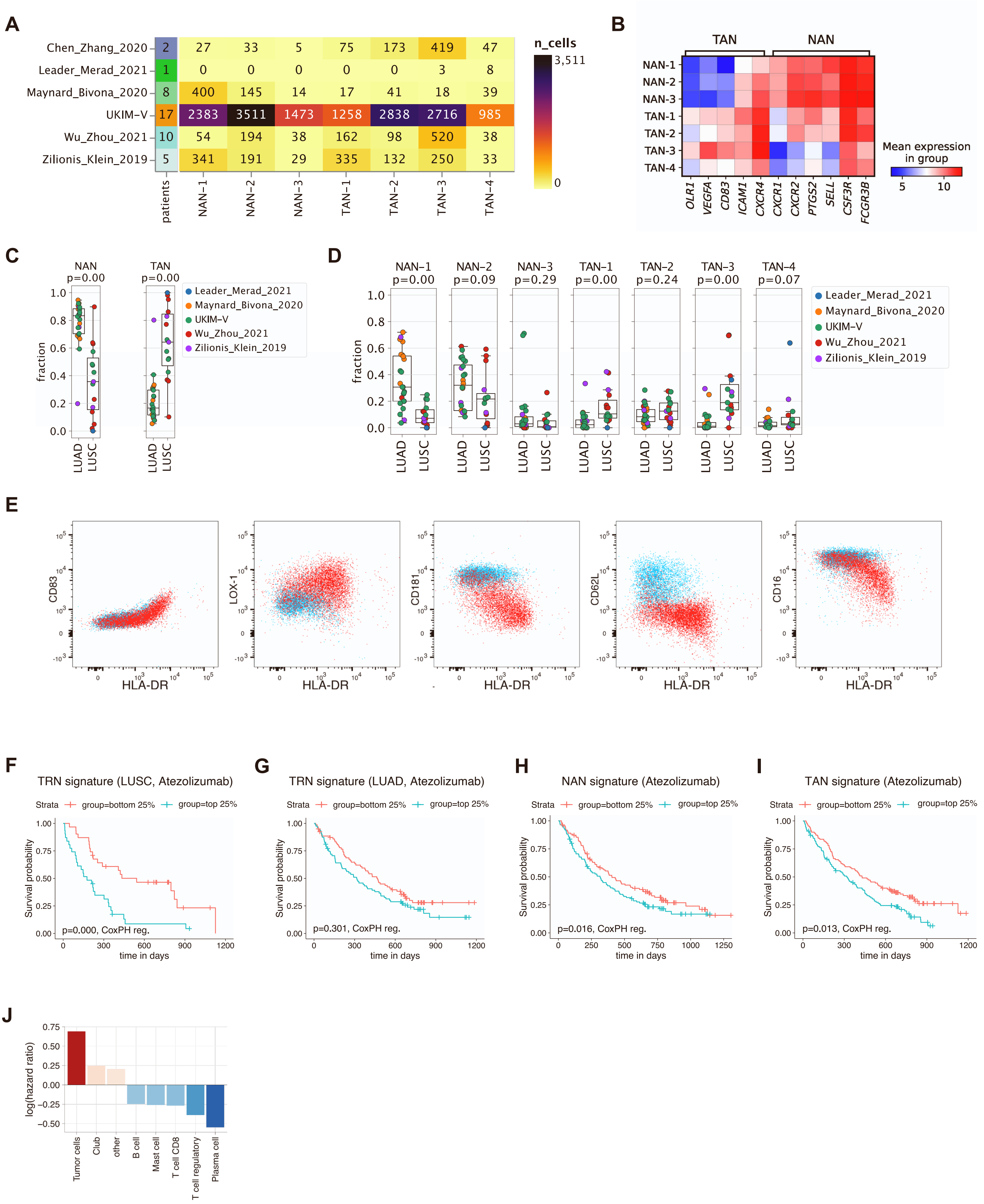
A
Chen_Zhang_2020
Leader_Merad_2021
Maynard_Bivona_2020
UKIM-V
Wu_Zhou_2021
Zilionis_Klein_2019
2 27 33 5 75 173 419 47
1 0 0 0 0 0 3 8
8 400 145 14 17 41 18 39
17 2383 3511 1473 1258 2838 2716 985
10 54 194 38 162 98 520 38
5 341 191 29 335 132 250 33
patients
NAN-1
NAN-2
NAN-3
TAN-1
TAN-2
TAN-3
TAN-4
n_cells
3,511
0
B
TAN
NAN
NAN-1
NAN-2
NAN-3
TAN-1
TAN-2
TAN-3
TAN-4
OLR1
VEGFA
CD83
ICAM1
CXCR4
CXCR1
CXCR2
PTGS2
SELL
CSF3R
FCGR3B
Mean expression in group
5
10
C
NAN p=0.00
TAN p=0.00
fraction
LUAD
LUSC
Leader_Merad_2021
Maynard_Bivona_2020
UKIM-V
Wu_Zhou_2021
Zilionis_Klein_2019
D
NAN-1 p=0.00
NAN-2 p=0.09
NAN-3 p=0.29
TAN-1 p=0.00
TAN-2 p=0.24
TAN-3 p=0.00
TAN-4 p=0.07
fraction
LUAD
LUSC
Leader_Merad_2021
Maynard_Bivona_2020
UKIM-V
Wu_Zhou_2021
Zilionis_Klein_2019
E
CD83
LOX-1
CD181
CD62L
CD16
HLA-DR
F
TRN signature (LUSC, Atezolizumab)
Strata group=bottom 25% group=top 25%
Survival probability
p=0.000, CoxPH reg.
time in days
G
TRN signature (LUAD, Atezolizumab)
Strata group=bottom 25% group=top 25%
Survival probability
p=0.301, CoxPH reg.
time in days
H
NAN signature (Atezolizumab)
Strata group=bottom 25% group=top 25%
Survival probability
p=0.016, CoxPH reg.
time in days
I
TAN signature (Atezolizumab)
Strata group=bottom 25% group=top 25%
Survival probability
p=0.013, CoxPH reg.
time in days
J
log(hazard ratio)
Tumor cells
Club
other
B cell
Mast cell
T cell CD8
T cell regulatory
Plasma cell

Figure S6: related to Figure 6. Tissue-resident neutrophil subtypes in NSCLC.

(A) TRN subclusters by the contributing datasets. Left column shows the number of patients with >10 neutrophils in the respective study, the heatmap depicts the number of cells per neutrophil cluster. Data of 43 patients with each > 10 neutrophils.

(B) TAN/NAN candidate marker gene expression by neutrophil subclusters. Expression values are the mean across pseudobulk samples by patient.

(C) NAN and TAN fractions in LUAD *versus* LUSC. Each dot refers to a patient with at least 10 neutrophils. P-values are derived from a t-test and not adjusted for multiple testing. In all boxplots, the central line denotes the median. Boxes represent the interquartile range (IQR) of the data, whiskers extend to the most extreme data points within 1.5 times the IQR.

(D) Neutrophils subcluster fractions in LUAD versus LUSC. Each dot refers to a patient with at least 10 neutrophils. P-values are derived from a t-test and not adjusted for multiple testing. In all boxplots, the central line denotes the median. Boxes represent the interquartile range (IQR) of the data, whiskers extend to the most extreme data points within 1.5 times the IQR.

(E) Flow cytometry analysis demonstrating the correlation between HLA-DR expression and CD83, LOX-1, CD181, CD62L or CD16 expression, respectively. Representative analysis of neutrophils derived from NSCLC normal-adjacent tissue (blue) and tumor tissue (red) are shown.

(F) Kaplan-Meyer plot of LUSC patients form the POPLAR (Fehrenbacher et al., 2016) and OAK (Rittmeyer et al., 2017) cohorts treated with atezolizumab with high (top 25%) and low (bottom 25%) TRN signature score. P-value has been determined using CoxPH-regression.

(G) Kaplan-Meyer plot of LUAD patients form the POPLAR (Fehrenbacher et al., 2016) and OAK (Rittmeyer et al., 2017) cohorts treated with atezolizumab with high (top 25%) and low (bottom 25%) TRN signature score. P-value has been determined using CoxPH-regression.

(H) Kaplan-Meyer plot comparing patients treated with atezolizumab with high (top 25%) and low (bottom 25%) NAN signature scores. P-value has been determined using CoxPH-regression including cohort and histology as covariates.

(I) Kaplan-Meyer plot comparing patients treated with atezolizumab with high (top 25%) and low (bottom 25%) TAN signature scores. P-value has been determined using CoxPH-regression including cohort and histology as covariates.

(J) Predictive value of cell-type signatures in bulk RNA-seq data from the OAK[80] and POPLAR[79] cohorts of NSCLC patients treated with atezolizumab (anti-PD-L1). The bar charts show cell-type signatures that are associated with worse (log hazard ratio > 0) or better (log hazard ratio < 0) survival at an FDR < 0.1. The hazard ratio and p-values have been determined using CoxPH regression including cohort and histology as covariates.

Table S4: related to Figure 6. Genes signatures.

TRN signature (n=38)	NAN signature (n=20)	TAN signature (n=18)
AGO4	AGO4	CCR3
ARG1	ARG1	CCRL2
CCR3	CYP4F3	DDIT3
CCRL2	ERGIC1	FLOT1
CYP4F3	FLOT2	HIF1A
DDIT3	FRAT2	IRAK2
ERGIC1	LRP10	MAFF
FLOT1	MGAM	MAP1LC3B2
FLOT2	MMP25	MCOLN1
FRAT2	MSRB1	NBN
HIF1A	NDEL1	NOD2
IRAK2	NFE2	PI3
LRP10	PADI4	PLAU
MAFF	PBX2	PPIF
MAP1LC3B2	PHOSPHO1	TGM3
MCOLN1	RASGRP4	TOM1
MGAM	REPS2	UBR5-AS1
MMP25	SULT1B1	ZNF267
MSRB1	TSEN34	
NBN	XKR8	
NDEL1		
NFE2		
NOD2		
PADI4		
PBX2		
PHOSPHO1		
PI3		
PLAU		
PPIF		
RASGRP4		
REPS2		
SULT1B1		
TGM3		
TOM1		
TSEN34		
UBR5-AS1		
XKR8		
ZNF267		

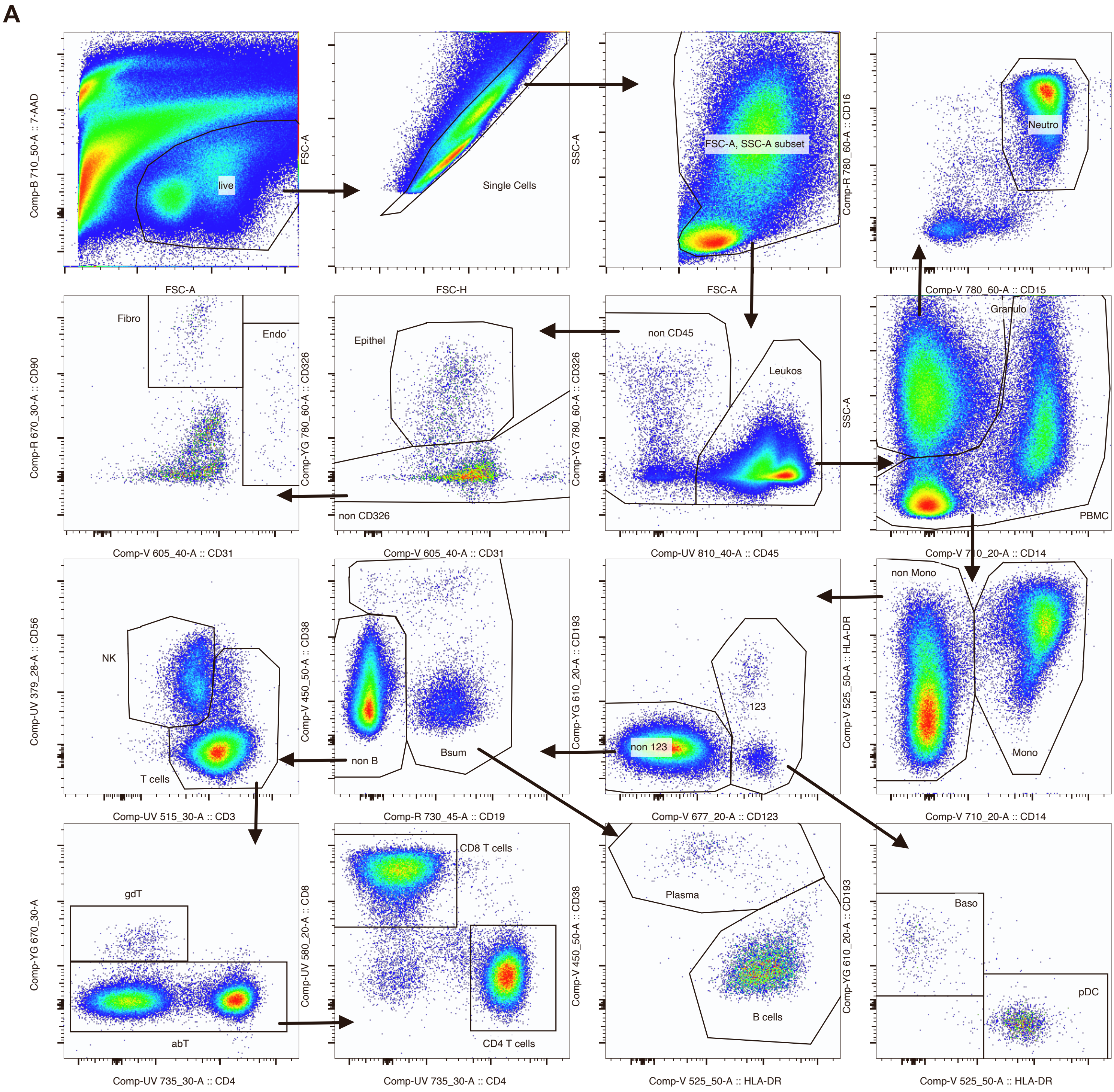

Figure S7 related to STAR Methods. Flow cytometry gating strategy to define cell populations from NSCLC tumor tissue and normal adjacent tissue.

(A) In initial cleaning steps dead cells, debris and doublets were removed using 7-AAD staining and scatter characteristics. Leukocytes were defined by CD45 staining and sequentially gated into subtypes including neutrophils, monocytes, T cells and B cells. Non-CD45⁺ cells were gated into epithelial cells, endothelial cells and fibroblasts.

Table S7: related to STAR Methods. Overview of antibody information, imultispectral imaging, and mmunohistochemistry.

Antibody cocktails used for flow cytometry:

Backbone		Cell definition		Neutrophil Characterization	
**Antigen**	**Fluorochrome**	**Antigen**	**Fluorochrome**	**Antigen**	**Fluorochrome**
CD56	BUV395	CD28	BUV615P	CD54	FITC
CD3	BUV496	CD38	BV421	CD83	FITC
CD8	BUV563	CD123	BV650	CD49b	PE
CD4	BUV737	CD34	FITC	CD62L	PE
CD45	BUV805	CD161	PE	LOX-1	PE
HLA-DR	BV480	CD193	PE-CF594	CD181	APC
CD31	BV605	TCRgd	PE-Cy5		
CD14	BV711	CD90	APC		
CD15	BV786				
CD326	PE-Cy7				
CD19	APC-R700				
CD16	APC-eF780				

Antibody-antigen retrieval and opal fluorophore pairing related to multispectral imaging:

Antibody	pH (AR)	Opal Pairing	Clone	Provider	Dilution
CXCR2	9	570	EPR22301-103	Abcam	1:500
LOX-1	9	540	polyclonal	Sigma-Aldrich	1:200
CD16	9	650	EPR22409-124	Abcam	1:600
CD8	9	570	C8\144B	Dako/Agilent	1:200
CD3	6	620	polyclonal	Dako/Agilent	1:250
CD68	9	650	PG-M1	Dako/Agilent	1:200
CD20	6	540	L26	Dako/Agilent	1:200
DAPI	7.4	450	-	Akoya Biosciences	1:15
Cytokeratin	9	450	AE1/AE3	Dako/Agilent	1:500
Cytokeratin	9	690	C-11	Abcam	1:1000

Overview of antibodies used for immunohistochemistry:

Antibody	Clone	Provider	Dilution
CD4	SP35	Ventana	pre-diluted
CD68	KP1	Ventana	pre-diluted
CXCR2	EPR22301-103	Abcam	1:500

Table S8: related to STAR methods. Quality control thresholds related to datasets integrated into the NSCLC single-cell atlas.

Dataset	min counts	max counts	min genes	max genes	max pct_mito
Adams_Kaminski_2020_COPD	1000	35000	500	10000	20
Chen_Zhang_2020_NSCLC	600	30000	250	10000	20

Goveia_Carmeliet_2020_NSCLC	600	30000	250	10000	20
Guo_Zhang_2018_NSCLC	20000	3000000	1000	20000	20
Habermann_Kropski_2020_pulmonary-fibrosis	600	30000	200	10000	20
Kim_Lee_2020_LUAD	1000	35000	300	10000	20
He_Fan_2021_LUAD	600	30000	250	10000	20
Lambrechts_2018_LUAD_6149v1	600	30000	200	10000	15
Lambrechts_2018_LUAD_6149v2	600	30000	250	10000	20
Lambrechts_2018_LUAD_6653	1200	40000	250	10000	20
Laughney_Massague_2020_NSCLC	1800	40000	500	10000	20
Madissoon_Meyer_2020_pulmonary-fibrosis	600	30000	300	10000	20
Maier_Merad_2020_NSCLC	1000	30000	400	10000	15
Maynard_Bivona_2020_NSCLC	20000	20000000	600	20000	30
Mayr_Schiller_2020_pulmonary-fibrosis	600	30000	250	10000	10
Reyfman_Misharin_2018_pulmonary-fibrosis	1000	30000	250	10000	20
Travaglini_Krasnow_2020_Lung_10x	1000	30000	500	10000	0
Travaglini_Krasnow_2020_Lung_SS2	20000	6000000	600	20000	30
UKIM-V	2000	100000	200	8000	30
Vieira_Teichmann_2019_asthma	600	30000	200	10000	20
Wu_Zhou_2021_NSCLC	600	30000	300	10000	30
Zilionis_Klein_2019_NSCLC	600	30000	200	10000	20
UKIM-V-2	1000	60000	200	8000	30
Leader_Merad_2021_10x_3p_v1_sort	600	30000	220	10000	10
Leader_Merad_2021_10x_3p_v2_beads_cite	600	30000	300	10000	25
Leader_Merad_2021_10x_3p_v2_beads	1000	30000	500	10000	20
Leader_Merad_2021_10x_3p_v2_digest-deadcell_cite	1000	30000	500	10000	20
Leader_Merad_2021_10x_3p_v2_sort	600	30000	250	10000	25
Leader_Merad_2021_10x_3p_v3_beads	600	30000	250	10000	30
Leader_Merad_2021_10x_5p_v1_beads	1100	30000	500	10000	25
Leader_Merad_2021_10x_5p_v1_CD2	1100	30000	500	10000	15
