# Supplementary material for: High-resolution single-cell atlas reveals diversity and plasticity of tissue-resident neutrophils in non-small cell lung cancer
Source: Cancer Cell. 2022 Dec 12;40(12):1503–1520.e8. doi: 10.1016/j.ccell.2022.10.008 (PMC9767679; doi:10.1016/j.ccell.2022.10.008)
Supplement: Document S2. Article plus supplemental information [file mmc6.pdf]

Cancer Cell

Article

High-resolution single-cell atlas reveals diversity and plasticity of tissue-resident neutrophils in non-small cell lung cancer

Graphical abstract

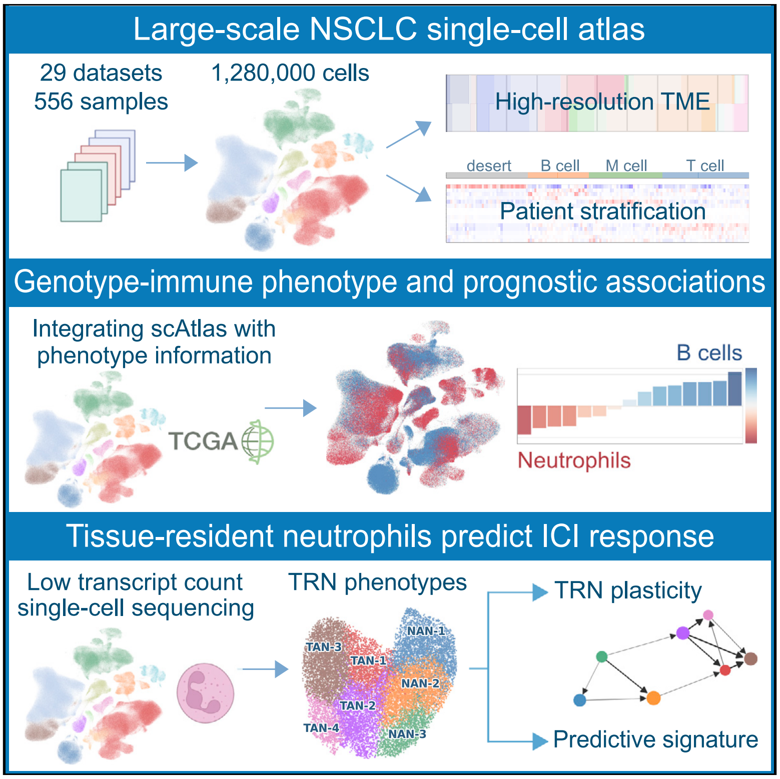

Highlights

- High-resolution single-cell atlas of the tumor microenvironment (TME) in NSCLC
- Histological tumor subtypes and driver genes imprint specific cellular TME patterns
- In-depth characterization of tissue-resident neutrophil (TRN) subpopulations
- TRN gene signature identifies patients refractory to treatment with PD-L1 inhibitor

Authors

Stefan Salcher, Gregor Sturm, Lena Horvath, ..., Dominik Wolf, Andreas Pircher, Zlatko Trajanoski

Correspondence

andreas.pircher@i-med.ac.at (A.P.),
zlatko.trajanoski@i-med.ac.at (Z.T.)

In brief

Salcher et al. integrate non-small cell lung cancer (NSCLC) single-cell datasets, refine patient stratification, and reveal histological and genotypic associations with tumor microenvironment (TME) composition. Single-cell sequencing of cells with low mRNA content identifies tissue-resident neutrophil (TRN) subpopulations with non-canonical functional properties. TRN gene signature is associated with immune-checkpoint inhibitor treatment failure.

Salcher et al., 2022, Cancer Cell *40*, 1503–1520
December 12, 2022 © 2022 The Authors. Published by Elsevier Inc.
https://doi.org/10.1016/j.ccell.2022.10.008

CellPress

Cancer Cell

CellPress
OPEN ACCESS

Article

High-resolution single-cell atlas reveals diversity and plasticity of tissue-resident neutrophils in non-small cell lung cancer

Stefan Salcher,[1,11] Gregor Sturm,[2,11] Lena Horvath,[1,11] Gerold Untergasser,[1] Christiane Kuempers,[3] Georgios Fotakis,[2] Elisa Panizzolo,[2] Agnieszka Martowicz,[1,4] Manuel Trebo,[1] Georg Pall,[1] Gabriele Gamerith,[1] Martina Sykora,[1] Florian Augustin,[5] Katja Schmitz,[4,6] Francesca Finotello,[7,8] Dietmar Rieder,[2] Sven Perner,[3,9,10] Sieghart Sopper,[1,12] Dominik Wolf,[1,12] Andreas Pircher,[1,12,*] and Zlatko Trajanoski[2,12,13,*]

[1]Department of Internal Medicine V, Haematology & Oncology, Comprehensive Cancer Center Innsbruck (CCCI) and Tyrolean Cancer Research Institute (TKFI), Medical University of Innsbruck, Innsbruck, Austria
[2]Biocenter, Institute of Bioinformatics, Medical University of Innsbruck, Innsbruck, Austria
[3]Institute of Pathology, University of Luebeck and University Hospital Schleswig-Holstein, Campus Luebeck, Luebeck, Germany
[4]Tyrolpath Obrist Brunhuber GmbH, Zams, Austria
[5]Department of Visceral, Transplant and Thoracic Surgery, Medical University Innsbruck, Innsbruck, Austria
[6]INNPATH GmbH, Institute of Pathology, Innsbruck, Austria
[7]Institute of Molecular Biology, University of Innsbruck, Innsbruck, Austria
[8]Digital Science Center, University of Innsbruck, Innsbruck, Austria
[9]Pathology, Research Center Borstel, Leibniz Lung Center, Borstel, Germany
[10]German Center for Lung Research (DZL), Luebeck and Borstel, Germany
[11]These authors contributed equally
[12]These authors contributed equally
[13]Lead contact
*Correspondence: andreas.pircher@i-med.ac.at (A.P.), zlatko.trajanoski@i-med.ac.at (Z.T.)
https://doi.org/10.1016/j.ccell.2022.10.008

SUMMARY

Non-small cell lung cancer (NSCLC) is characterized by molecular heterogeneity with diverse immune cell infiltration patterns, which has been linked to therapy sensitivity and resistance. However, full understanding of how immune cell phenotypes vary across different patient subgroups is lacking. Here, we dissect the NSCLC tumor microenvironment at high resolution by integrating 1,283,972 single cells from 556 samples and 318 patients across 29 datasets, including our dataset capturing cells with low mRNA content. We stratify patients into immune-deserted, B cell, T cell, and myeloid cell subtypes. Using bulk samples with genomic and clinical information, we identify cellular components associated with tumor histology and genotypes. We then focus on the analysis of tissue-resident neutrophils (TRNs) and uncover distinct subpopulations that acquire new functional properties in the tissue microenvironment, providing evidence for the plasticity of TRNs. Finally, we show that a TRN-derived gene signature is associated with anti-programmed cell death ligand 1 (PD-L1) treatment failure.

INTRODUCTION

Non-small cell lung cancer (NSCLC) is a highly aggressive and heterogenous disease with diverse histological subtypes and distinct mutational signatures,[1] which accounts for an annual global death rate of 1.8 million patients.[2] The technical advances in single-cell RNA sequencing (scRNA-seq) technologies enabled the dissection of the complex NSCLC tumor microenvironment (TME) in different stages, and numerous scRNA-seq NSCLC studies have identified a hitherto underestimated TME heterogeneity in early and advanced disease.[3–13] Furthermore, these studies highlighted the importance of small cell populations in governing essential biological pathways such as immune cell activation or trafficking by tumor endothelial cells.[6] However, a major limitation of these studies is the limited number of analyzed patient samples per study. Moreover, the lack of genomic data as well as long-term follow-up data prevents comprehensive dissection of the biological heterogeneity and its potential contribution to therapy resistance and survival outcome.

Technical and methodological variations between the different studies result in significant inconsistencies and knowledge gaps. As such, not all cell types (e.g., neutrophilic granulocytes) have been portrayed in the same depth and extension yet, posing an unmet need to characterize these populations as well. In NSCLC, it is well accepted that next to cancer cells, leukocytes

This is an open access article under the CC BY license (http://creativecommons.org/licenses/by/4.0/).

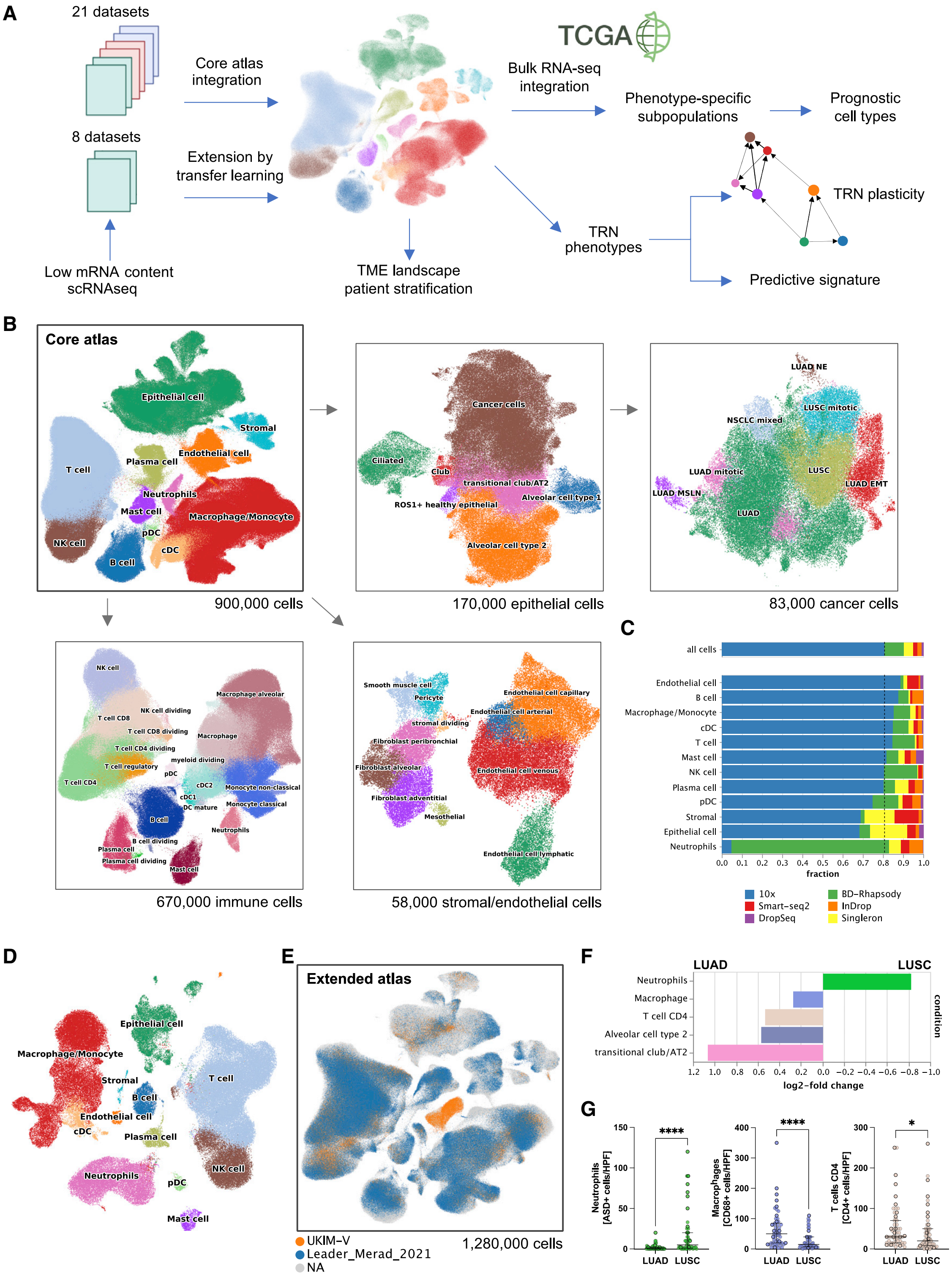

Figure 1. Schematic outline of the overall concept used in this study

(A) Summary of the data integration and analysis workflow.

(B) Overview of the core NSCLC atlas and the epithelial, immune, and stromal/endothelial components depicted as uniform manifold approximation and projection (UMAP) plots.

(C) Fractions of depicted cell types per scRNA-seq platform.

(D) UMAP of the UKIM-V dataset (n = 17) colored by cell type.

(E) Core atlas extended by Leader[11] and UKIM-V-2 datasets.

(legend continued on next page)

CellPress
OPEN ACCESS

compose the majority of cells within the TME.[12,14] Particularly since immunotherapy is routinely used in clinical practice, in-depth characterization of the cancer immune cell compartment has been intensively pushed forward, and diverse cellular subsets have been profiled.[5,9,15] Previous compositional analyses by flow cytometry as well as histological work ups suggested that neutrophils compose a significant proportion of all tumor-resident leukocytes, with an estimated abundance ranging from 8% to 20%.[14,16,17] Intriguingly, when looking at the scRNA-seq studies in NSCLC published over the last years, neutrophils are clearly underrepresented. This discrepancy is most likely based on technical issues rather than on biological phenomena, but its clarification is of immense importance for our fundamental immunological understanding of NSCLC and for potential translational clinical investigations. This notion is underscored by pre-clinical data suggesting that neutrophils are essential mediators of both pro- and anti-tumor inflammatory pathways (reviewed in Shaul and Fridlender[18]), including the potential of neutrophils to limit lymphocyte trafficking into malignant tumors, thereby limiting efficacy of programmed cell death 1 (PD-1) inhibition.[19] Correlative studies in patients with NSCLC have linked the neutrophil:lymphocyte ratio with clinical outcome and response to immunotherapy.[19–21] Additionally, pre-clinical evidence strongly supports the use of neutrophil-depleting agents (e.g., CXCR2 antagonists) as an adjunction to immune-checkpoint inhibitors.[19]

To overcome the above-mentioned hurdles, we compiled major publicly available datasets into a comprehensive NSCLC scRNA-seq atlas covering 232 patients with NSCLC and 86 non-cancer controls. Additionally, given the scarcity of neutrophil single-cell data, we complemented the atlas by analyzing samples from 17 patients with NSCLC using a platform that captures cells with very low transcript count and carried out deep characterization of tissue-resident neutrophils (TRNs) including both tumor-associated neutrophils (TANs) and normal adjacent tissue-associated neutrophils (NANs).

RESULTS

Generation of a core large-scale NSCLC single-cell atlas

We first developed a core NSCLC atlas by compiling scRNA-seq data from 19 studies and 21 datasets comprising 505 samples from 298 patients (Figure 1A). This comprehensive NSCLC single-cell atlas integrates expert-curated, quality-assured, and pre-analyzed transcriptomic data from publicly available studies as well as our own dataset (UKIM-V) in early and advanced stage NSCLC of any histology (see STAR methods; Figures S1A–S1K). Important study characteristics are summarized in Table S1. In total, the core atlas includes transcriptomic data from 212 patients with NSCLC and 86 control individuals, comprising 196 tumor samples and 168 non-tumor control samples. Of the 212 patients with NSCLC, 156 were histopathologically annotated as lung adenocarcinoma (LUAD), 41 as lung squamous cell carcinoma (LUSC), and 15 were not otherwise specified (NSCLC NOS). NSCLC samples include tissue of the primary tumor (n = 176) or metastasis (n = 45) that were obtained either by surgical resection or by computed tomography- and bronchoscopy-guided biopsies. We clustered the disease stages of the patients with NSCLC as early (UICC stage I–II) versus advanced (UICC III–IV) diseases, as not all studies provided sufficient information on tumor stages. Among the control samples, 89 were derived from distant non-malignant tissue of patients with lung tumors (annotated as normal_adjacent), of which 65 have a patient-matched tumor sample. Further, 10 samples were derived from non-tumor-affected lymph nodes of patients with NSCLC (annotated as normal) and 79 samples from patients without evident lung cancer history (annotated as normal). Of the control patients, 18 had a history of chronic obstructive pulmonary disease (COPD). Overall, the core atlas integrates 898,422 single cells, which are annotated to 12 coarse cell-type identities and 44 major cell subtypes or cell states (e.g., dividing cells) based on previously established canonical single-cell signatures (Figure S1A) including 169,223 epithelial cells, 670,409 immune cells, and 58,790 stromal and endothelial cells (Figure 1B). We also annotated important CD8$^+$ T cell subclusters (terminally exhausted, activated, effector memory, naive, natural killer [NK]-like, dividing) using previously reported marker genes[22] (Figure S1J). The cell-type composition for each dataset, the tissue of origin, and the patients within the core atlas are shown in Figures S1B and S1C.

Previous scRNA-seq studies discriminated the clinically relevant types of LUSC and LUAD. The UKIM-V dataset was histopathologically classified by routine pathologists followed by independent review by expert lung pathologists (S.P. and C.K.). Cancer cells (in total, 83,439 cells, from primary tumor and metastatic tissue) in the atlas showed high heterogeneity of their transcriptomic profiles (Figure 1B). Due to the large patient cohort, we were able to apply high-resolution lung cancer cell classification based on their specific marker gene expression signatures (Figure S1D). We divided the following main clusters LUSC (*KRT5*, *KRT6A*, *KRT17*, *SOX2*, *NTRK2*, *TP63*); LUAD (*CD24*, *MUC1*, *KRT7*, *NAPSA*, *NKX2-1*, *MSLN*); LUAD with signs of epithelial-to-mesenchymal transition (LUAD EMT) (*VIM*, *SERPINE1*, *CDH1*, *MIF*); LUAD with neuroendocrine features (LUAD NE) (*CHGA*, *SYP*, *NCAM1*, *TUBA1A*); LUAD with high expression of mesothelin (*MSLN*) associated with EMT and metastasis (LUAD MSLN);[23] and NSCLC expressing both LUAD and LUSC markers (NSCLC mixed) (*MUC1*, *KRT7*, *KRT6A*, *SOX2*) (Figures 1B and S1D). There are highly mitotic/proliferative clusters (*TOP2A*, *MKI67*) of both LUAD and LUSC that may resemble highly aggressive and invasive cancer cells. The LUAD EMT cells likely resemble an invasive, pro-metastatic cluster characterized by the plasminogen activator PAI1 (*SERPINE1*) or the mesenchymal protein vimentin (*VIM*) that are both involved in cell adhesion, invasion, and angiogenesis.[24,25] Besides, all subclusters showed a high expression of the conserved non-coding RNAs

(F) Cell-type composition by histopathological tumor type (LUAD, LUSC). FDR = 0.1.
(G) Immunohistochemistry staining of neutrophils (ASD$^+$ cells), macrophages (CD68$^+$ cells), and T cells CD4 (CD4$^+$ cells) per high-power field (HPF) in LUAD (n = 55) versus LUSC (n = 55). Evaluation was performed by two separate expert lung pathologists (C.K. and S.P.). The horizontal line represents the median, and whiskers extend to the interquartile range; Wilcoxon test, *p < 0.05, ****p < 0.0001.
See also Figures S1 and Tables S1, S2, and S3.

MALAT1 and *NEAT1* (Figure S1D) that were previously linked to metastasis formation in NSCLC.[26,27] For all subsequent analyses, we used the histopathological annotation LUAD or LUSC.

Neutrophils are underrepresented in most scRNA-seq studies

In both tumor and normal lung tissue of all core atlas samples, the neutrophil cluster (*FCGR3B*, *CSF3R*, *CXCR2*, and *G0S2*) comprised 8,468 cells with overt low mRNA counts. Overall, neutrophils account for only 1.5% of all atlas cells (Figure S1E). Remarkably, 78% of all atlas neutrophils derive from the UKIM-V dataset (Figures 1C and S1B), in which neutrophils compose 12% of all cells and 18% of all leucocytes, respectively. The remaining neutrophil data originate mainly from four other datasets,[4,5,10,12] in all of which neutrophils comprise less than 6% of all leucocytes. Particularly in those studies using the droplet-based 10x Chromium platform, neutrophils are completely absent or only rarely depicted (Figure S1F). Our comparative flow cytometry analysis demonstrated that neutrophils account for 10%–20% of all leucocytes in NSCLC tumor and normal adjacent tissues (n = 63) (Figure S1G), which is in accordance with previous flow cytometry and histology data.[14,16,17] Thus, low neutrophil abundance seen in previous scRNA-seq datasets suggests an underrepresentation, most likely due to technical issues. Neutrophils are fragile, short-lived cells (circulatory half-life of 7–10 h in humans[18]), are particularly sensitive to handling procedures, and express an exceptionally low amount of mRNA molecules.[4] Comparative analysis of scRNA-seq platforms indicated that the BD Rhapsody workflow captures a notably high number of mRNA molecules per cell and may thus be especially suitable to depict low-mRNA-content cells (Figure S1H). As a consequence, neutrophils represented a major cell cluster in the UKIM-V dataset generated with BD Rhapsody, whereas the low-mRNA-content neutrophil cluster could not be appropriately detected in the datasets generated with 10x Chromium, as also described recently,[28] and only to a very limited extend when applying other platforms (Figure 1C).

scRNA-seq of low-mRNA-content cells

Due to high clinical relevance of neutrophils and the need for their better in-depth transcriptomic characterization, we used the advantage of the BD Rhapsody platform in depicting cells with low mRNA content for further analysis. As our initial dataset included only three patients, we next performed scRNA-seq of an additional 14 patients with NSCLC to increase the statistical weight of our cohort (UKIM-V cohort). In total, our dataset contains tumor and adjacent normal lung tissue from 17 patients (6 male, 11 female) undergoing lobectomy for treatment-naive NSCLC (12 LUAD, 5 LUSC). Cells were freshly isolated, processed, and sequenced as described in detail in the STAR methods. The UKIM-V dataset (1 and 2) comprises 122,902 cells that cluster into all main lung cell types defined by the expression of specific marker genes (Figure 1D). Neutrophils are characterized as a cell cluster with exceptionally low mRNA content and thus a relatively low number of detected transcripts, but due to the relatively high number of mRNA molecules captured per cell (unique molecular identifier [UMI] counts in epithelial cells: 8,938), we could readily depict these cells. The 15,190 neutrophils identified in the UKIM-V dataset were derived from control lung (n = 6,378) and corresponding tumor tissue (n = 8,812).

Extension of the core single-cell atlas by transfer learning

To combine the strength of the core atlas with our own data including neutrophils, we used the recently developed transfer-learning method scArches,[29] which enables the extension of the core atlas using additional current, as well as future, datasets. We mapped our second UKIM-V dataset as well as one recently published dataset comprising 288,157 cells[11] onto the atlas (Figure 1E). The cell-type annotations were transferred from the core atlas to the two new datasets on the basis of transcriptomic similarity in the batch-corrected joint embedding (see STAR methods). This extended atlas now integrates 29 datasets from 19 studies and comprises 1,283,972 cells, 44 cell types, 556 samples, and 318 patients, resulting in 1.75 billion expression values. The overall cell-type composition of the extended atlas is shown in Figure S1I, and the patient numbers per cell type are shown in Table S2. All subsequent analyses were carried out on the extended atlas dataset unless otherwise specified (Table S3).

Identification of changes in cell-type compositions in distinct histological or genetic tumor types and tumor stages is of utmost importance as it can highlight hetero-cellular interactions and possibly enable association(s) with therapy response. However, detecting shifts in cell-type composition using scRNA-seq data is challenging due to the inherent bias present in cell-type compositions and low sample sizes. We therefore adopted a Bayesian model for identifying changes in cell-type compositions while controlling for the false discovery rate (scCODA tool[30]). scCODA requires setting a reference cell type that is assumed to be constant between conditions. When comparing cellular composition in LUSC and LUAD in primary tumor tissue with cancer cells as reference cell type, we found a significantly higher proportion of neutrophils in LUSC, whereas macrophages, $CD4^+$ T cells, alveolar cells type 2, and transitional club/AT2 were more abundant in LUAD (Figure 1F). The analysis of the cell-type compositions in early-stage compared with advanced-stage NSCLC tumors showed a higher abundance of cDC2 in the early stage (Figure S1K). To validate the findings, we carried out orthogonal validation using an external cohort of 110 patients with NSCLC (55 LUAD and 55 LUSC) and immunohistochemistry staining for neutrophils, $CD4^+$ T cells, and macrophages. The validation results confirmed the findings (Figure 1G) and further support histology-specific TME characteristics.

Single-cell composition of the TME reveals distinct NSCLC tumor immune phenotypes

Next, we stratified patients with NSCLC based on infiltration patterns of their TME using the extended atlas. Unsupervised clustering on batch-corrected cell-type fractions revealed four distinct tumor immune phenotypes (Figure 2A): (1) immune-deserted (ID) tumors (i.e., no significant immune cell infiltration but a high cancer cell fraction); (2) the subtype of tumors with B cell dominance (B; B cell, plasma cell, mast cells); (3) the subtype of tumors with myeloid dominance (M; macrophage/monocyte); and (4) the subtype of tumors with T cell dominance (T; $CD8^+$, $CD4^+$, T regulatory cells). The affiliation of UKIM-V

CellPress
OPEN ACCESS

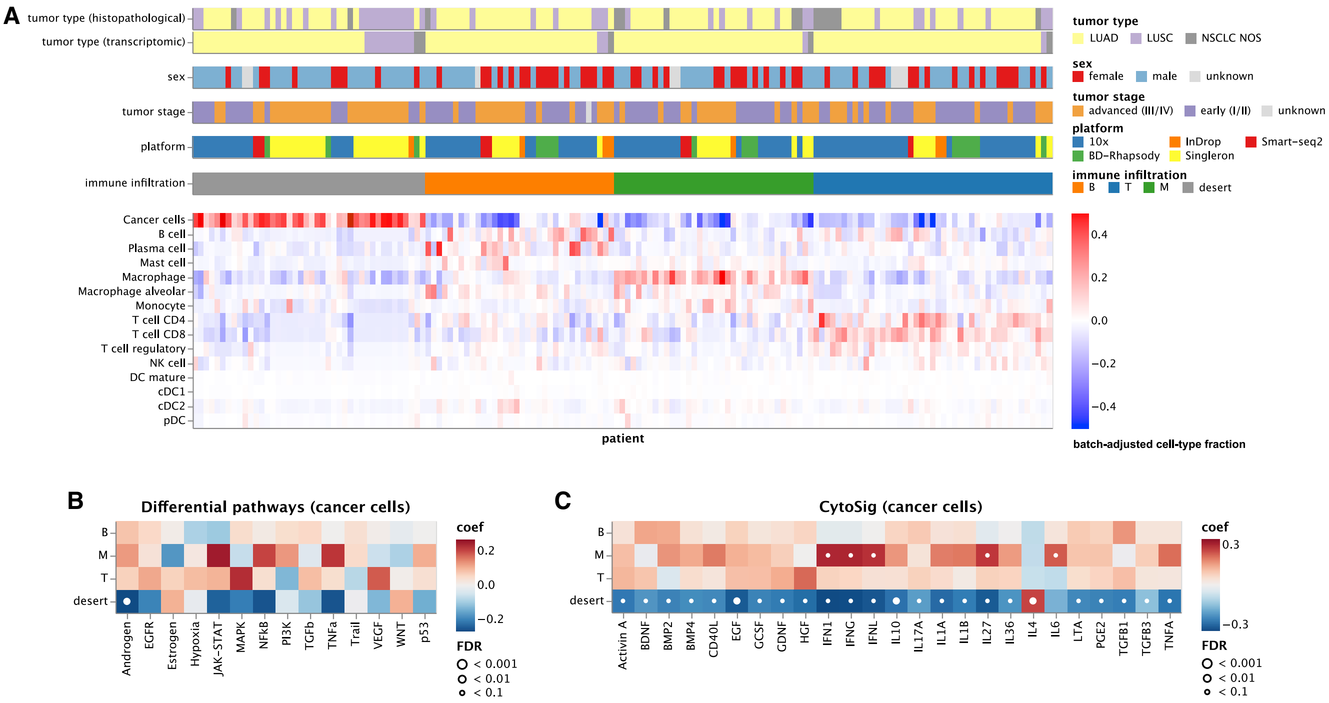

Figure 2. Tumor immune phenotypes in NSCLC

(A) Patient characteristics and stratification of the tumor immune phenotypes. Tumor type (histopathological) refers to the histological subtypes as provided by the original datasets based on pathological assessment; tumor type (transcriptomic) is based on the most abundant transcriptomically annotated cancer-cell subtype in the scRNA-seq atlas.

(B and C) Differential activation of (B) PROGENy cancer pathways and (C) CytoSig cytokine signaling signatures in cancer cells between the four tumor immune phenotypes. Heatmap colors indicate the deviation from the overall mean, independent of tumor histology and stage. White dots indicate significant interactions at different FDR thresholds. Only cytokine signatures with an FDR <0.1 in at least one patient group are shown.

See also Figure S2.

patients to myeloid cell and T cell subtypes was validated using flow cytometry (Figures S2A and S2B). Across the strata, most patients of the B cell, myeloid cell, and T cell subtypes showed LUAD histology, while half of the patients with LUSC were over-represented in the ID subtype (Figure S2C). Neutrophils were excluded from patient stratification since they are under-represented in most datasets. Using logistic regression, we did not find any association of the different patient strata to tumor stages (early versus advanced) or sex.

To identify tumor-cell-based TME imprinting characteristics, we next analyzed differentially enriched pathways[31] in the cancer cells of each of the four immune phenotypes. The ID subtype showed significant downregulation (false discovery rate [FDR] <0.1) of the androgen pathway (Figure 2B). Previously, androgen receptor signaling has been shown to suppress programmed cell death ligand 1 (PD-L1) transcription in hepatocellular carcinoma (HCC) cells and may thus exert immune stimulatory effects.[32] Analysis of differentially expressed transcription factors[33,34] in the cancer cells of each subtype showed a significant downregulation of *FOXO4* in the ID subtype (Figure S2D). As previously reported, FOXO transcription factors are essential mediators of immune cell homeostasis,[35] and *FOXO4* downregulation could thus promote an ID phenotype.

We then applied the tool CytoSig[36] to define enriched cytokine signaling signatures in cancer cells of each immune phenotype (Figure 2C). CytoSig analyzes defined cytokine signatures that are differentially expressed when a cell is exposed to a specific cytokine (that is name giving for the respective cytokine signature). As expected, most signatures are downregulated in the ID group; solely, the signature of the tolerogenic cytokine interleukin-4 (IL-4), a modulator of T regulatory cell-mediated immune suppression,[37] was significantly elevated (Figure 2C). We found a significant upregulation of interferon type I–III signatures in the myeloid subgroup, suggesting a particularly important role of macrophages/monocytes in interferon signaling in the TME (Figure 2C).

Analysis of cell-cell communication reveals hetero-cellular crosstalk in the TME

Using the CellPhoneDB ligand-receptor complexes database, we next assessed differences in the hetero-cellular crosstalk of cancer cells toward diverse immune cells among the two major histotypes LUAD and LUSC by analyzing the top 10 differentially expressed cancer cell ligands (Figure 3A; the top 30 ligands are shown in Figure S3A). Overall, in both histologies, cancer cellular interactions were directed to diverse immune cell subtypes with different targets. In LUAD, we found a prominent upregulation of the KDR-VEGFA axis from cancer cells toward neutrophils, macrophages/monocytes, mast cells, and classical dendritic cells (cDCs), potentially implicating immunosuppressive signaling by

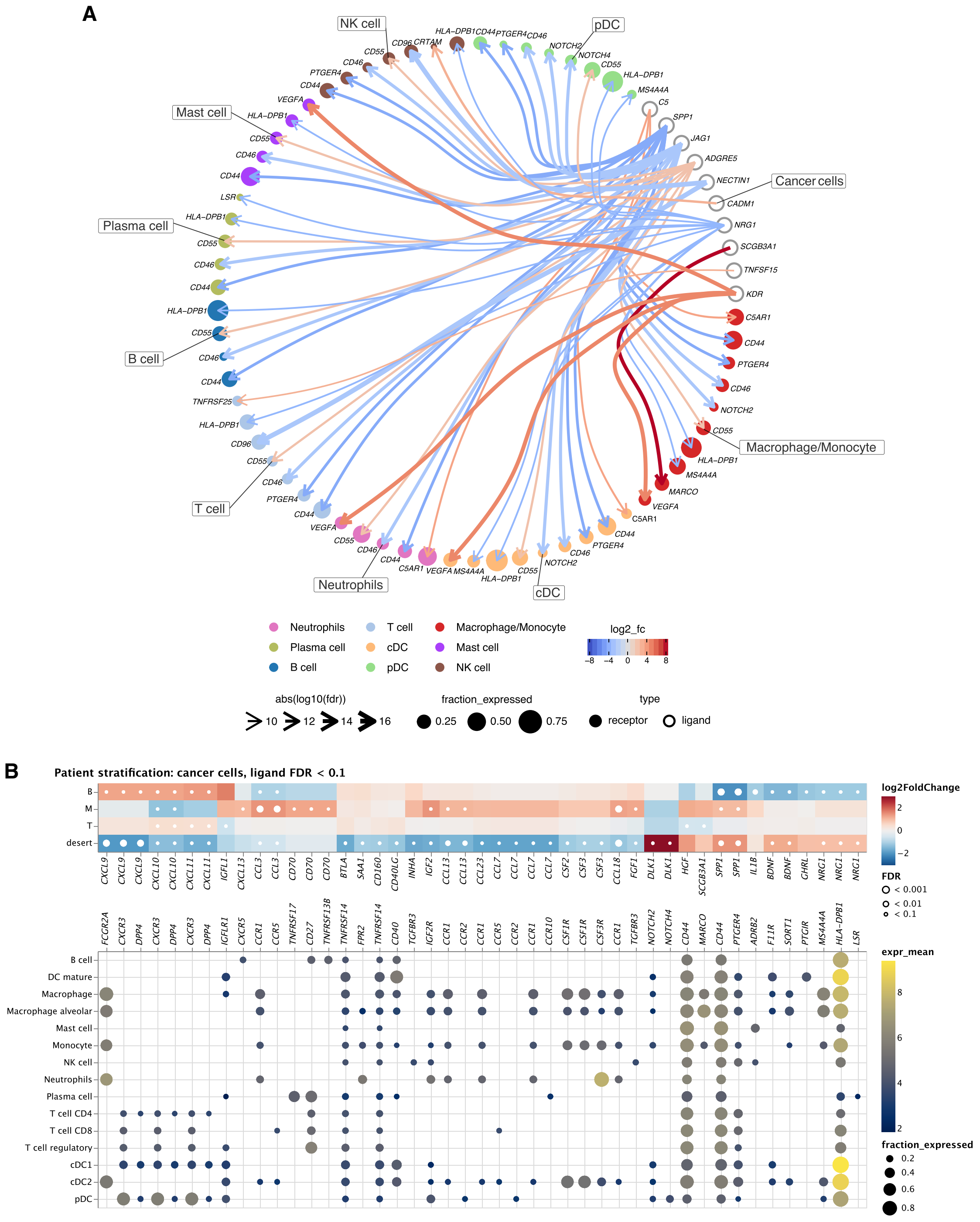

Figure 3. Cellular crosstalk analysis

(A) Circos plot of the cellular crosstalk of cancer cells toward the major immune cells in LUAD versus LUSC. Shown are the top 10 differentially expressed cancer cell ligands. Red interactions are upregulated in LUAD, and blue interactions are upregulated in LUSC.

(B) Cancer-immune cell crosstalk in each patient subtype. Top panel: differentially expressed ligands of cancer cells in each subtype (B, M, T, ID) (DESeq2 on pseudo-bulk, FDR < 0.1). Bottom panel: respective receptors and the expression by cell type. Dot sizes and colors refer to the fraction of cells expressing the receptor and gene expression, respectively, averaged over all patients. Dots are only shown for receptors that are expressed in at least 10% of the cells of the respective cell types.

See also Figure S3.

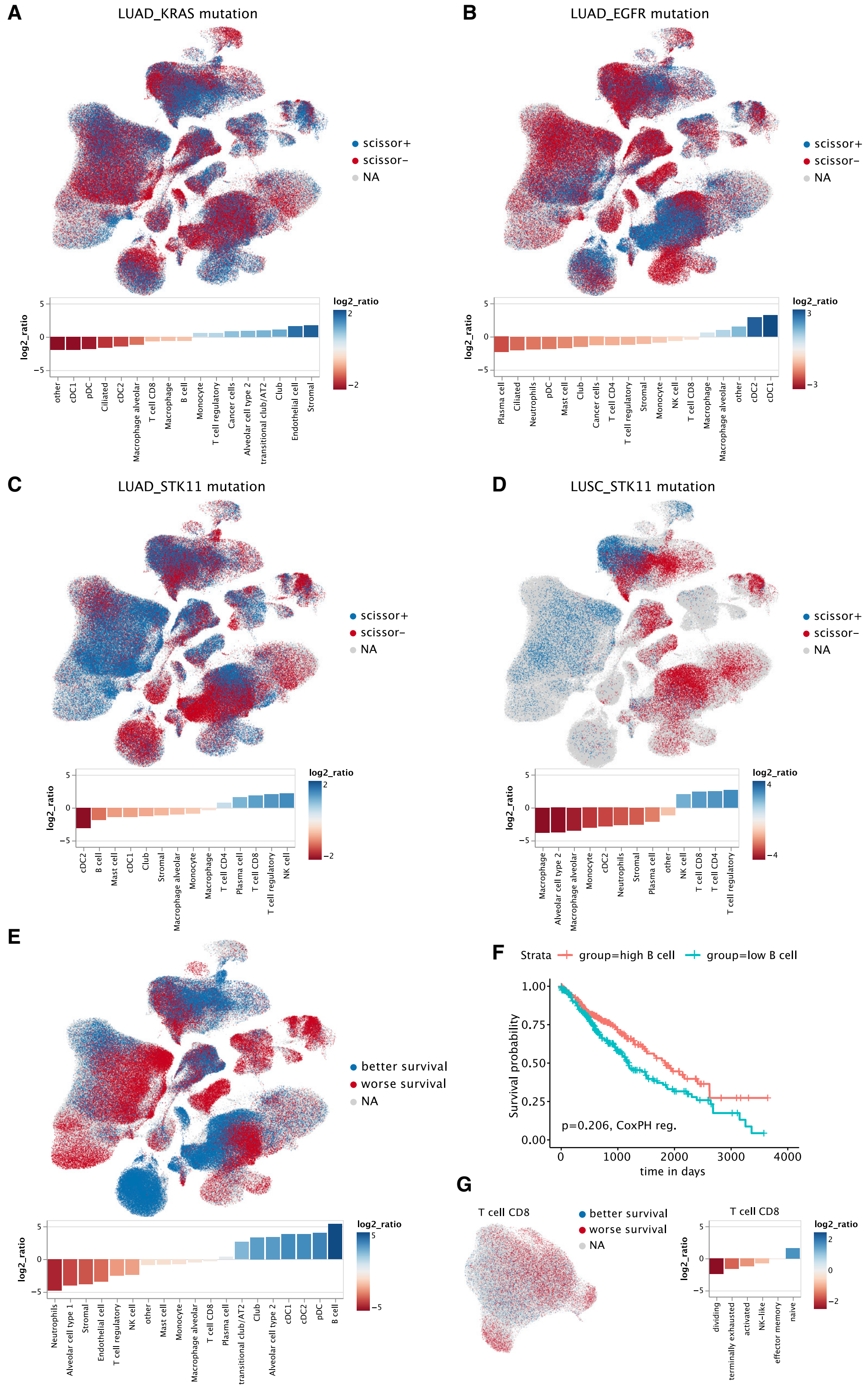

(legend on next page)

cancer cells in this histotype.[38] Other major LUAD pathways involve the immunosuppressive macrophage scavenger receptor MARCO[39] as well as ADGRE5-CD55 signaling, associated with migration and invasion.[40] Conversely, in LUSC cancer cells, there is upregulation of pro-migratory SPP1 signaling[41] that has previously been reported as upregulated in lung cancer tissue particularly of squamous histology,[42] as well as an upregulation of Jagged1 (JAG1), which induces NOTCH, thereby promoting tumor progression and regulating the tumor immune microenvironment via, e.g., neutrophil recruitment.[43]

Next, we investigated the crosstalk of cancer cells to immune cells within the patient immune subtypes T cell, B cell, myeloid cell, and ID by analyzing differentially expressed cancer cell ligands (Figure 3B). While downregulated in the ID subgroup, B, M, and lesser T cell subgroups showed upregulated signaling of several chemokines (CXCL9/10/11, CCL3/13/18) to their cognate receptors on T and myeloid cell subsets, suggesting that cancer-cell-secreted chemokine gradients contribute to immune infiltration.[44]

Integration of bulk RNA-seq data reveals genotype-immune phenotype associations

scRNA-seq provides an unprecedented view on the cellular heterogeneity in the TME. However, the majority of the scRNA-seq studies lack both cancer genotype information and survival data. The TCGA reference dataset includes this information together with bulk RNA-seq data. Using the recently published computational method SCISSOR,[45] we evaluated the association of atlas-derived cell-type transcriptomic signatures with genotype and survival data from the TCGA reference dataset including 1,026 patients (UICC I-IV, LUAD, and LUSC). In a previous pan-cancer study using bulk RNA-seq data, we have shown that genomic features including mutational load, tumor heterogeneity, and specific driver genes determine immune phenotypes.[46] Here, the high resolution of the single-cell NSCLC atlas enabled an in-depth analysis of these determinants. *EGFR*, *TP53*, *KRAS*, and *STK11* mutations showed distinct immune infiltrates (Figures 4A–4D, S4A, and S4B). For example, cDC1 and cDC2 showed opposite infiltration patterns in patients with LUAD with mutations of either *EGRF* (high cDC infiltration, as reported previously[47]) or *KRAS* and *STK11* (low cDC infiltration) (Figures 4A–4D). Conversely, *TP53*- and *STK11*-mutated genotypes were associated with $CD8^+$ T infiltration, which is not seen in *EGFR*- or *KRAS*-mutated tumors (Figures 4C, 4D, S4A, and S4B). High $CD8^+$ T cell infiltration in *TP53*-mutated LUAD has also been described previously.[47] Hence, our single-cell view of the TME provides further evidence for the link between the genetic makeup of the tumor, the histology, and the respective immune contexture.[48] Given the importance of the driver genes in terms of treatment decisions, we confirmed our findings by orthogonal validation of the genotype-immunophenotype associations using two external cohorts (n = 19 and n = 37) (Figures S4C and S4D).

We next analyzed the cell-type transcriptomic signatures and their association with survival in 1,026 patients from the TCGA cohort. Overall, patients with NSCLC with B cell-rich tumors showed a prominent association with improved survival, whereas neutrophils were the strongest negative survival predictor (Figure 4E) and were, together with monocytes, the only immune cell types that were negative predictors in both LUAD and LUSC (Figures 4E, S4E, and S4F). To support our finding, we used an independent method based on deconvolution using bulk RNA-seq data and confirmed that B cells are indeed associated with better prognosis, albeit significantly only for LUAD (Figures 4F, S4G, and S4H), which has also been proposed in multiple previous studies (reviewed in Patel et al.[49]). Finally, the analysis of the $CD8^+$ T cell subtypes showed that naive $CD8^+$ T cells were the strongest predictor for improved survival (Figure 4G).

TRNs acquire new properties in the TME

One unique feature of the large-scale NSCLC atlas we assembled is the enrichment with single-cell expression profiles from neutrophils generated using samples from 17 patients with NSCLC, so we focused on the deep characterization of these cells. TANs are known as a very heterogenous cell population with both anti- and pro-tumorigenic properties.[50] Likely due to technical reasons, the characterization of NANs lags even further behind that of TANs. To overcome this insufficient TRN characterization, we here characterized the transcriptomic signatures of TANs and NANs in NSCLC using the extended atlas (Figures 5A and 5B). Neutrophils were more abundant in patients with LUSC compared with those with LUAD (Figure S5A), as described previously,[12] which we confirmed in two external cohorts by flow cytometry analysis (47 LUAD and 16 LUSC) (Figure 5C) and immunohistochemistry (55 LUAD and 55 LUSC) (Figure 1G). The overall TAN phenotype was characterized by high expression of *OLR1* (LOX-1), *VEGFA*, *CD83*, *ICAM1*, and *CXCR4* and low expression of *CXCR1*, *CXCR2*, *PTGS2*, *SELL* (CD62L), *CSF3R*, and *FCGR3B* (CD16B) (Figure 5D), confirming previously reported signature genes.[18] The TAN characteristic gene set included expression patterns of both established

Figure 4. Association of cellular composition and distinct genotypes and survival in the TCGA data

(A–E and G) SCISSOR analysis relating phenotypic information from bulk RNA-seq data from TCGA with single cells. UMAP plots indicate the position of cells positively (blue) or negatively (red) associated with mutation or better survival. A log2 ratio >0 indicates a positive association with mutation or better survival, respectively. Shown are cell types with a log2 ratio significantly different from 0 at an FDR <0.01 (paired Wilcoxon test).

(A) Association of cellular composition with *KRAS* mutation in patients with LUAD (n = 156).

(B) Association of cellular composition with *EGFR* mutation in patients with LUAD (n = 98).

(C) Association of cellular composition with *STK11* mutation in patients with LUAD (n = 141).

(D) Association of cellular composition with *STK11* mutation in patients with LUSC (n = 83).

(E) Association of cellular composition with overall survival.

(F) Kaplan-Meyer plot of patients with high (top 25%) and low (bottom 25%) B cell fractions of TCGA patients with lung cancer as determined by deconvolution with EPIC. p value has been determined using CoxPH regression using tumor stage and age as covariates.

(G) Association of cellular composition with overall focusing on $CD8^+$ T cell subclusters. $CD8^+$ T cell subclusters were annotated based on gene sets from Oliveira et al.[22]

See also Figure S4.

CellPress
OPEN ACCESS

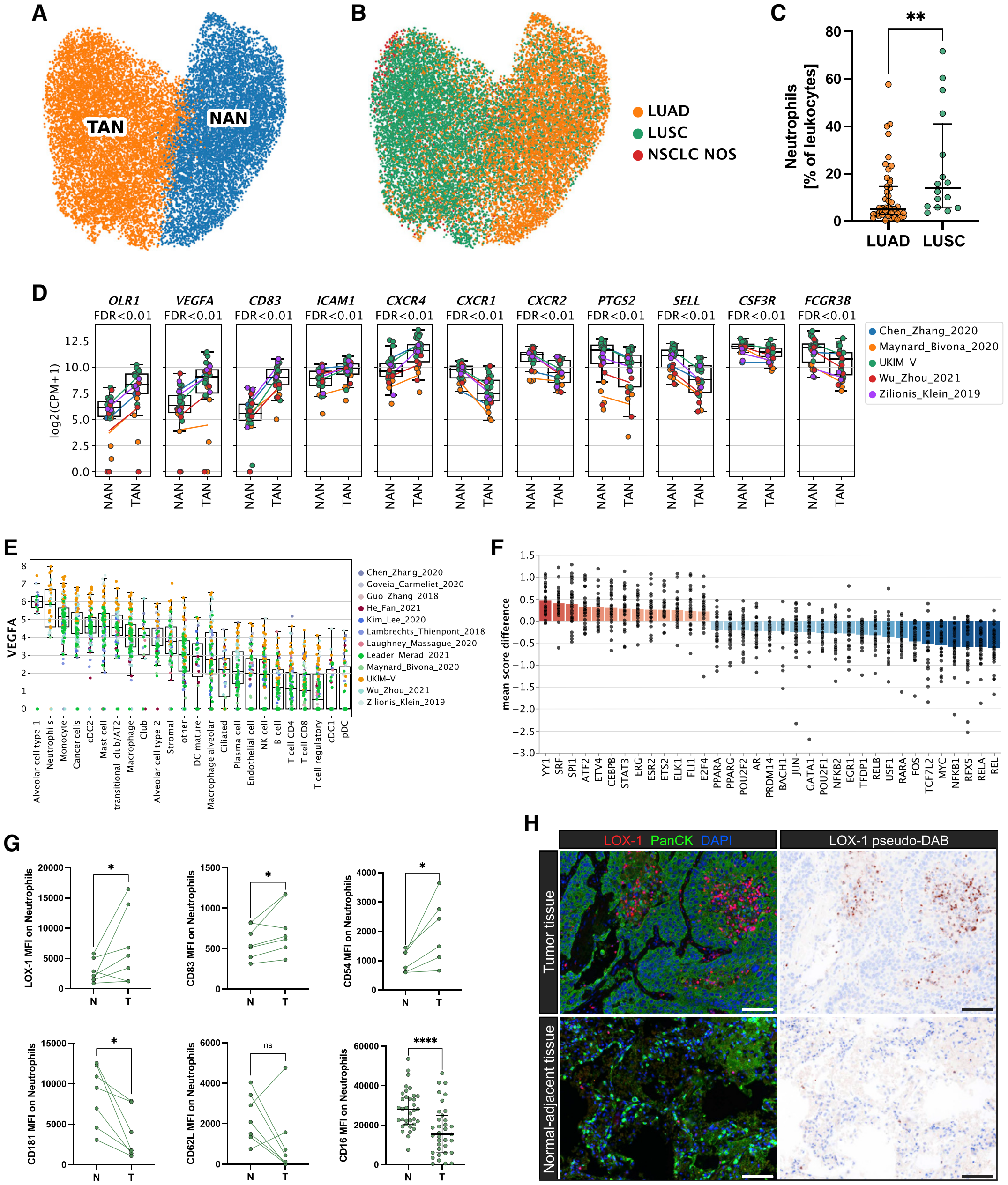

Figure 5. Characterization of tissue-resident neutrophils using scRNA-seq

(A and B) UMAP of tissue-resident neutrophils (TRNs) from the extended atlas, (A) classified into tumor-associated neutrophils (TANs) and normal-adjacent associated neutrophils (NANs) and (B) colored by histology (as defined by histopathological assessment).

(C) Neutrophil fractions (as percentage of leucocytes) by flow cytometry of LUAD and LUSC tumor tissue (LUAD n = 47, LUSC n = 16; Wilcoxon test, **$p < 0.01$). The horizontal line represents the median, and whiskers extend to the interquartile range.

(D) Candidate TAN genes. Each dot refers to a patient with at least 10 neutrophils in both NAN and TAN groups. Lines indicate the mean per study. p values are derived from a paired t test and adjusted for FDR.

(E) Expression levels of *VEGFA* in various cell types in primary tumor samples. Each dot represents a patient with at least 10 cells (median values, boxes represent the interquartile range [IQR], whisker data points within 1.5 times the IQR).

(F) Transcription factor analysis of TAN versus NAN using DoRothEA. Each dot represents a single patient, and bars are the mean of all patients. p values are derived using a paired t test and are FDR adjusted. Shown are transcription factors with a mean score difference >0.2 and an FDR <0.1.

(G) Comparison between tumor and normal-adjacent samples for selected candidate genes using flow cytometry. Each dot represents a patient that was not part of the scRNA-seq dataset. Paired Wilcoxon test, *$p < 0.05$, ****$p < 0.0001$. CD16: the horizontal line represents the median, and whiskers extend to the IQR.

(H) Selected multiplex immunofluorescence (M-IF) staining of LOX-1 (red) and pancytokeratin (green) in tumor tissue and matched normal-adjacent lung tissue of a patient with LUSC. Scale bar: 100 μm.

See also Figure S5.

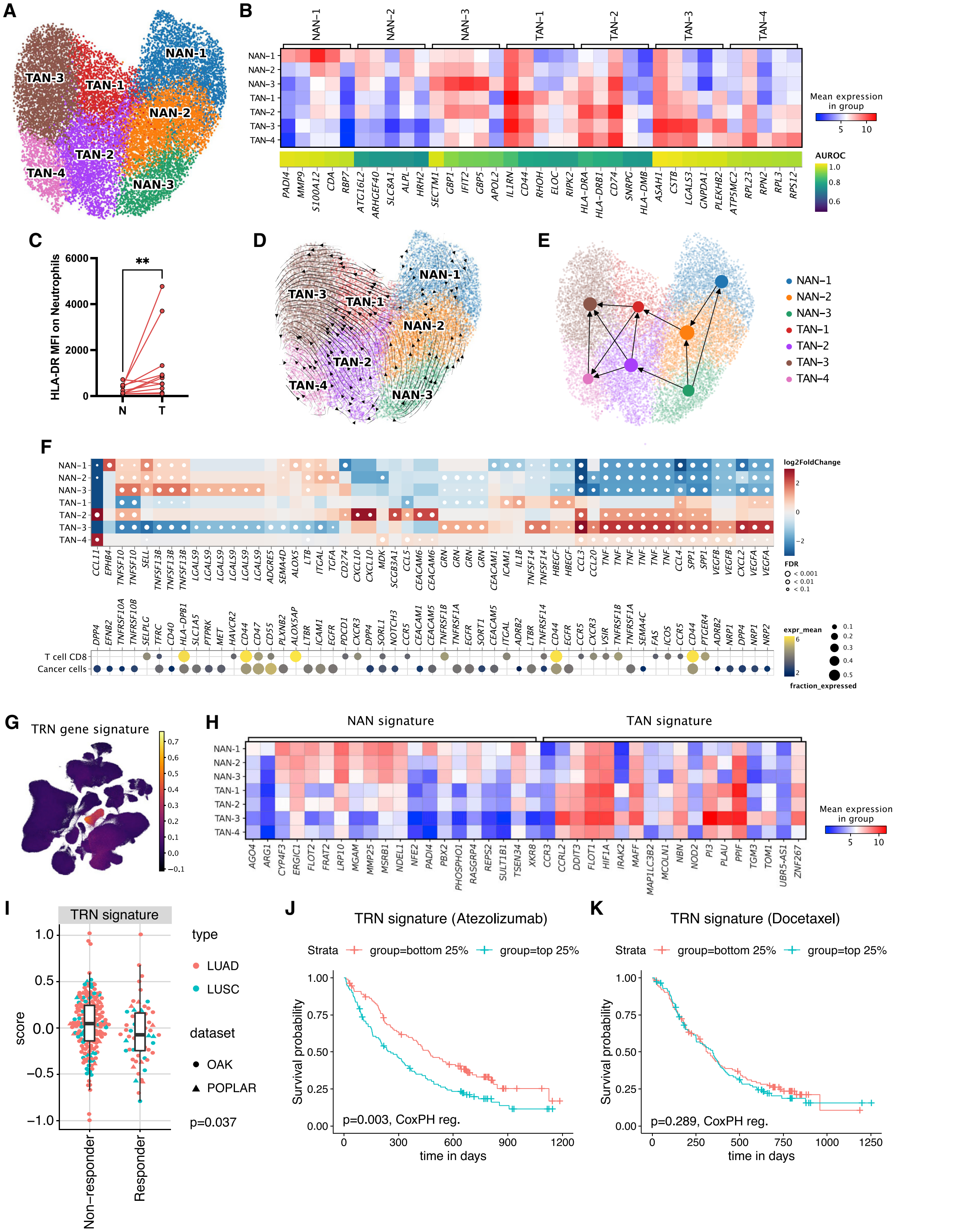

Figure 6. Tissue-resident neutrophil subtypes in NSCLC

(A) UMAP of all TRNs colored by TAN and NAN subclusters. The neutrophil clusters derive from 85 patients, 42 of whom have >10 neutrophils.

(B) Top 5 markers for each TAN and NAN cluster. The marker gene quality is reflected by the area under the receiver operator characteristics curve (AUROC; 1 = marker gene perfectly distinguished the respective cluster from other clusters in all patients; AUROC 0.5 = no better than random).

(legend continued on next page)

neutrophil markers (*CXCR1*, *CXCR2*, *CXCR4*, *PTGS2*) as well as novel candidates (*OLR1*, *VEGFA*, *CD83*), as discussed below.

In a recent study, remarkable neutrophil adaptability to different tissue environments was shown,[51] suggesting that while transient, TRNs acquire new properties and function within tissue. The neutrophil phenotype differs in dependence on spatial-, temporal-, and disease-specific clues[52] as well as during the evolution from bone-marrow-resident immature (CXCR4^{high}, CXCR2^{low}, CD16^{low}, CD62L^{low}, MME^{low}), to circulating/mature (CXCR2^{high}, CD16^{high}, CD62L^{high}), to aged/senescent neutrophils (CXCR4^{high}).[53–55] However, none of these markers are specific for a certain maturation state. Relative to TANs, matched NANs in our dataset showed high expression of established neutrophil maturity markers (*SELL*, *PTSG2*, *CXCR2*, *CXCR1*, *FCGR3B*, *MME*) as well as canonical neutrophil markers (*S100A8*, *S100A9*, *S100A12*) (Figures 5D and S5B). While downregulation of these markers in TANs suggests immaturity, we could not identify a clear expression pattern of previously suggested immaturity signatures.[55,56] Notably, *CXCR1* and *CXCR2* have previously been reported as TAN markers in NSCLC;[4,12] however, our analysis revealed elevated *CXCR1*/*CXCR2* expression in NANs (Figure 5D). Conversely, low expression of *SELL* (CD62L) and *CXCR2* (both downregulated in aged neutrophils[57]) (Figure 5D) and high expression of the known neutrophil activation markers *CD83* (an inhibitory immune checkpoint),[58,59] the atypical chemokine receptor *CCRL2*,[60] *ICAM1* (CD54),[57] *and C15orf48* (a mitochondrial transcript upregulated during inflammation)[61] as well as several cytokines (*CCL3*, *CCL4L2*, *CCL4*, *CXCL2)* (Figures 5D and S5B) support an aged/chronically activated/exhausted TAN phenotype.[62]

Neutrophils support the pro-angiogenic switch in cancer via release of VEGF and other pro-angiogenic factors (reviewed in Ozel et al.[63]). Our atlas provided evidence that neutrophils represent a major source for *VEGFA* expression within the NSCLC TME (Figure 5E). A highly differentially expressed TAN marker of major interest is lectin-type oxidized LDL receptor 1 (LOX-1) encoded by the *OLR1* gene that is known as main receptor for oxidized low-density lipoprotein (LDL)[64] (Figures 5D and S5B). *OLR1* has been described as a putative marker to distinguish normal peripheral blood neutrophils (LOX-1^{-}) from polymorphonuclear myeloid-derived suppressor cells (PMN-MDSCs),[65] respectively. However, concordant to our results, *OLR1* expression by TANs has been previously described,[12] and our comparative analysis to matched NANs underlined the tumor specificity of this marker. Moreover, peroxisome proliferator-activated receptor gamma (*PPARG*), a nuclear receptor and direct transcriptional regulator of *OLR1*,[66] was elevated in TANs (Figure 5F). Concordantly, flow cytometry analysis of tissue from patients with NSCLC (n = 7) confirmed elevated LOX-1 (*OLR1*) expression in TANs (Figure 5G). We could further validate the transcriptomic TAN signature at the protein level by flow cytometry, including elevated expression of CD83 (n = 7) and CD54 (*ICAM1*) (n = 6) and lower expression of CD181 (*CXCR1*), CD62L (*SELL*) (n = 8), and CD16 (n = 35) (Figure 5G).

We additionally performed multiplex immunofluorescence staining of paraffin-embedded NSCLC tumor tissue and patient-matched normal adjacent lung tissue. Co-staining of LOX-1 and CXCR2 suggested LOX-1 as neutrophil marker (Figure S5C) (of note, CXCR2 also marks MDSCs[67]). We found infiltration of LOX-1^{+} cells in tumor tissue but not in adjacent normal-lung tissue (Figure 5H), underlining the cancer-tissue specificity of this marker.

Plasticity and non-canonical functional properties of TRNs

Previous studies have proposed transcriptomic subclusters of neutrophils in NSCLC.[4] However, a distinct subclassification and in-depth characterization of TRN including TANs and NANs in NSCLC has not been described so far. We applied unsupervised Leiden clustering on all atlas neutrophils (n = 19,166), separating four TAN subsets (TAN-1 to TAN-4) and three NAN subsets (NAN-1 to NAN-3) (Figure 6A) that are backed by multiple datasets and multiple patients (Figure S6A). Marker gene selection revealed an extensive phenotypic heterogeneity among the clusters and allowed identification of marker genes for each subcluster, of which the top 5 are given in Figure 6B. The TAN signature genes described above (*OLR1*, *VEGFA*, *CXCR4*, *CD83*) showed relative homogenous expression among all TAN subclusters (Figure S6B). Overall, NAN clusters showed a predominance in LUAD and TAN clusters in LUSC tumors (Figures S6C and S6D).

Specific NAN-1 genes included the alarmin *S100A12*, a known marker of activated proinflammatory neutrophils,[68] and the

(C) Quantification of HLA-DR expression by flow cytometry of tumor and normal-adjacent tissue. Each dot represents the mean of each patient. Paired Wilcoxon test, **p < 0.01.

(D) UMAP of TRNs from the UKIM-V dataset with RNA velocity vectors projected on top.

(E) Partition-based graph abstraction (PAGA) based on RNA velocities, projected on the UMAP plot. Edges represent cell-type transitions called by PAGA.

(F) Outgoing interactions of TRN subclusters with cancer cells and CD8^{+} T cells. Top panel: differentially expressed ligands in each subcluster (FDR <0.01, abs. log2 fold change >1). Heatmap colors clipped at ±3. Bottom panel: respective receptors and the expression by cell type. Dots are only shown for receptors that are expressed in at least 10% of the respective cell types.

(G) UMAP of the extended atlas colored by the score of the TRN gene signature (38 genes with high specificity for TRNs)

(H) Heatmap of the TAN and NAN gene signatures across the TRN subclusters. Colors indicate the mean gene expression across patients in the respective clusters.

(I–K) Predictive value of the TRN signature in bulk RNA-seq data from the OAK[80] and POPLAR[79] cohorts of patients with NSCLC treated with atezolizumab (anti-PD-L1) or docetaxel (chemotherapy).

(I) Comparison of non-responders (progressive disease) with responders (complete response, partial response) treated with atezolizumab, shown for each histotype.

(J) Kaplan-Meyer plot comparing patients treated with atezolizumab with high (top 25%) and low (bottom 25%) TRN signature scores. p value has been determined using CoxPH regression including cohort and histology as covariates.

(K) Kaplan-Meyer plot comparing patients treated with docetaxel with high (top 25%) and low (bottom 25%) TRN signature scores.

See also Figure S6 and Tables S4 and S5.

NETosis co-factor *PADI4*,[69] as well as pro-angiogenic markers (*PROK2*, *MMP9*). The NAN-2 cluster is pretty similar to the NAN-1 cluster but showed reduced expression of some NAN-1-specific genes (e.g., *S100A12*, *PADI4*, *MMP9)*. NAN-3 shows strong expression of interferon-stimulated genes (*GBP1*, *GBP5*, *IFIT2*) (Figure 6B). TAN-1 shows high expression of interleukin 1 receptor antagonist (*IL1RN*), a known marker of activated neutrophils that negatively regulates IL-8 secretion to control excessive neutrophil inflammatory activity,[70] as well as of the potent NF-κB activator *RIPK2*[71] and *CD44*, regulating cell recruitment and adhesion[72] (Figure 6B). These finding support the concept that the plasticity of neutrophils is profoundly shaped by the NSCLC TME that attracts and activates neutrophils.

The TAN-2 subcluster was characterized by the expression of the major histocompatibility complex (MHC) class II genes *HLA-DRA*, *CD74*, *HLA-DMB*, and *HLA-DRB1*, indicating a phenotype with an immunogenic antigen-presenting feature. Both *CD74* and *HLA-DRA* are also expressed in the other TAN clusters, albeit at lower levels (Figure 6B). To validate the scRNA-seq findings, we analyzed samples from an additional 11 patients using flow cytometry, confirming the antigen-presenting phenotype as seen by an upregulation of HLA-DR on TANs in NSCLC compared with neutrophils from normal adjacent lung tissue (Figure 6C). Of note, the transition to HLA-DR$^+$ neutrophils was accompanied by a shift toward the identified TAN signature (elevated expression of CD83 and LOX-1 and lower expression of CD181 [*CXCR1*], CD62L [*SELL*], and CD16) in neutrophils derived from NSCLC tumor tissue (Figure S6E).

The TAN-3 subcluster was characterized by a high expression of proinflammatory cytokines (*C15orf48*, *CCL3*, *CCL4*, *CSTB*) as well as galectin 3 (*LGALS3*), which is associated with neutrophil activation and emigration (Figure 6B). Finally, TAN-4 showed high expression of ribosomal genes (such as *RPS12*, *RPL3*, *RPN2*, *RPL23*) (Figure 6B) similar to a neutrophil cluster identified in patients with severe COVID-19.[56] This may suggest the highly plastic phenotype of TAN-4 eventually transitioning to other cell phenotypes, as described previously for tumor endothelial cells.[6]

The transcriptional profiles of the neutrophil subsets indicate their remarkable phenotypic plasticity. We therefore performed RNA velocity analysis (see STAR methods) using only the UKIM-V dataset (which includes treatment-naive patients with NSCLC only) since the method requires raw sequencing data. The analysis indicates a transition from NAN-3 to both NAN-2 and NAN-1 (whose transcriptomic signatures are similar). NAN-3 and NAN-2 transition to TAN-2 and TAN-1, respectively, with TAN-1 and TAN-2 transitioning into all TAN subtypes (Figures 6D and partition-based graph abstraction[73] in 6E). Interestingly, this TRN evolution seems to follow a one-directory path with TAN-3 as final transition, although at this point we do not know whether TAN-3 could further transit to other cell types. These observations support the hypothesis that TAN phenotypes are substantially modulated by local cues encountered in the TME.[18]

We next investigated the cellular interactions of TRN subsets with CD8$^+$ T cells and cancer cells by analyzing differentially expressed TRN ligands (FDR <0.01, abs(log2FC) >1) of each subset, revealing distinct signaling of NANs versus TANs (Figure 6F). In all TAN subsets, we found *VEGFA* signaling toward cancer cells, again underlining their important proangiogenic role, as well as *SPP1* signaling, which has been associated with an immunosuppressive TME[74] and pro-migratory[75] effects. *CD274* (PD-L1) to *PDCD1* (PD-1) signaling is significantly upregulated in TAN-2 while being downregulated in NAN-1, proposing CD8$^+$ T cell-inhibitory effects of TAN that accord well with previous observations of impeded immunotherapy responses in neutrophil-rich tumors.[19] Conversely, NANs showed prominent interactions involving genes of the tumor-necrosis family (*TNFSF13B*, *TNFSF10*, *LTB*) that have been previously associated with neutrophil activation.[76,77]

TRN gene signature is associated with immune-checkpoint inhibitor treatment failure

Our deconvolution of the diversity of TRNs at the single-cell level prompted us to relate this information to patient prognosis and response to both chemotherapy and therapy with immune-checkpoint inhibitors (ICIs). Using a previously proposed approach for finding specific marker genes for cell-type estimation from bulk RNA-seq samples[78] (see STAR methods), we derived a signature of genes that are highly specific for TANs (n = 18) or NANs (n = 20) and are expressed only at a very low level in other cells (Figure 6G). We additionally defined a TRN signature as the union of TAN and NAN signature genes (n = 38) (Table S4). The expression of the TRN signature genes was heterogeneous between the different TRN subsets (Figure 6H). In order to analyze the prognostic and predictive value of the TRN signature, we used bulk RNA-seq data from pre-treatment tumors from POPLAR[79] and OAK,[80] two randomized clinical trials of anti-PD-L1 antibody (atezolizumab) versus chemotherapy (docetaxel) in patients with NSCLC, representing the largest transcriptional collection in these settings.[81] In total, there were 891 patients, of which 439 were treated with atezolizumab (316 LUAD and 123 LUSC) and 452 with docetaxel (313 LUAD and 139 LUSC). The TRN gene signature was associated with anti-PD-L1 therapy failure in these NSCLC cohorts (Figure 6I). Analysis of the survival data from these cohorts showed that the prognostic benefit of the TRN signature was significant for the anti-PD-L1 arm (Figure 6J) but not for the docetaxel arm (Figure 6K). The prognostic value for the anti-PD-L1 arm was stronger for LUSC (Figure S6F) compared with LUAD (Figure S6G). The signatures for the different subsets TRN, namely NANs and TANs, were both predictive for the anti-PD-L1 arm of the NSCLC cohorts (Figures S6H and S6I). The results for the signatures for other cell types (Table S5) are shown in Figure S6J.

DISCUSSION

We built a large-scale atlas of single-cell transcriptomes of NSCLC through integration of 29 datasets spanning over 1,280,000 cells from 556 samples and 318 individuals representing 1.75 billion expression values. Reduction of dataset-specific batch effects due to variation in experimental design and used platforms while retaining biological information resulted in a high-quality reference atlas for NSCLC, offering superior coverage of histological and clinical variables and thereby providing a unique resource for dissecting the cellular diversity in the TME and generating hypotheses. We leveraged the information content of the NSCLC atlas by sequencing additional patient samples using a platform suitable

to depict low-mRNA-content cells, which enabled us to comprehensively characterize the diversity and plasticity of TRNs.

First, we provide a high-resolution view of the TME in NSCLC with 44 major cell types/states and show different cell-type composition patterns in LUAD and LUSC, including more precise functional transcriptomic classification of malignant epithelial cells in both histotypes. The single-cell composition of the TME in NSCLC enabled refined tumor classification and patient stratification into four immune phenotypes: ID, myeloid, B cell, and T cell subtypes. These findings may have important implications for improving cancer immunotherapy in NSCLC. For example, combination therapies that target both myeloid cells and lymphoid cells could represent an immunotherapeutic strategy to treat myeloid subtypes, as shown recently in a melanoma mouse model.[82] Similarly, given the heterogeneity of the intratumoral B cells and the importance of tertiary lymphoid structures,[83] further analysis of the B cell subtype might open promising therapeutic avenues by additional refined B cell targeting.

Second, integration of bulk RNA-seq data from the TCGA NSCLC cohort uncovered cell subsets associated with alterations in major driver genes, such as *EGFR*, *KRAS*, *STK11*, and *TP53*, in both LUAD and LUSC subtypes, providing further evidence that genetic aberrations in cancer cells dictate the immune contexture of tumors.[48] We validated the findings from the computational analysis using two independent cohorts and immunofluorescence/immunohistochemistry assays, confirming previously published reports on genotype/immunophenotype dependencies.[47,84,85] This knowledge could be exploited to derive rationale for personalized therapeutic combination strategies based on the underlying genetic tumor profile.

Third, we provide in-depth characterization of TRNs including both TANs and NANs in human NSCLC. Our dissection of the diversity of TANs suggest that the conflicting reports can be attributed to the different TRN subsets. Of particular interest for cancer immunotherapy is the TAN phenotype with an immunogenic antigen-presenting feature. This observation implies acquisition of antigen-presenting-like properties by neutrophils at the tumor site, as previously reported.[86] Such conversion of neutrophils to antigen-presenting cells may elicit anti-tumor immunity and has recently been shown in a murine model.[87] Identification of targets that can block the transition of the antigen-presenting TAN-2 subset into TAN-1 and TAN-3 or reverse the final phenotypes into TAN-2 phenotype is an important goal for future studies.

Finally, we report that the TRN-derived gene signature has a predictive and prognostic effect of the TRN signals for immunotherapy-treated patients with NSCLC. Using transcriptomic data for patients with NSCLC (n = 439) from two randomized clinical trial cohorts treated with a single anti-PD-L1 antibody (atezolizumab), we provide evidence for the association of TRNs with therapy failure. Although not statistically significant for the chemotherapy arm, the similarity of the graphs for both drugs fits the general paradigm that neutrophil infiltration is associated with worse anti-tumor outcomes.

Beyond these biological insights, the results from this study have also important implications. Specifically, the diversity and plasticity of TRNs shown here further underscore the necessity to reevaluate the rationale for targeting neutrophils to overcome ICI therapy resistance in combination therapies using CXCR1 and CXCR2 antagonists and other inhibitors.[88] As shown here, TANs can acquire antigen-presenting properties, and such conversion of abundant neutrophils to antigen-presenting cells could overcome the limitations of the low abundance of cross-presenting DCs.[87] We advocate that rigorous approaches are required to analyze the impact of the TRN diversity and plasticity on tumor immunity in NSCLC and possibly in other cancers.

Our study, however, has several limitations. First, NSCLCs show a great intratumor heterogeneity, and the sampling location (e.g., tumor core versus tumor margin) may affect the cellular composition, particularly the in case of biopsies being compared with tissue pieces from lobe resections. With the exception of one study,[3] all studies incorporated in our atlas applied single regional sampling without annotation of the exact sampling area. Thus, our analyses do not take into account this variable, and we therefore advocate that future studies should include information about the sampling location. Second, in many cases, mRNA is not a definite proof of the extent to which a protein is expressed, and information on both RNA and protein expression is necessary for getting a complete picture of gene regulation and single-cell heterogeneity. In neutrophils, this particularly regards granule protein expression that varies throughout granulopoiesis, not always strictly correlating with mRNA expression.[89] This could partly explain the above-mentioned conflicting literature results of TAN phenotypes. And third, albeit the association of the TRN signature with anti-PD-L1 treatment failure was analyzed using transcriptomic data for patients with NSCLC, prospective studies are required to show that the TRN signature indeed represents a bona fide anti-PD-L1 therapy outcome-predicting marker rather than being a negative prognostic marker.

In conclusion, we provide a NSCLC atlas with single-cell resolution as well as a web portal that enables interactive exploration of the dataset through cell-x-gene (https://luca.icbi.at) that allows visualization of metadata and gene expression. The biological insights we present here and future discoveries arising from the exploitation of the high-resolution NSCLC atlas could provide the basis for developing combination therapies for patients with NSCLC who are not sufficiently responding to immune-checkpoint blockers.

STAR★METHODS

Detailed methods are provided in the online version of this paper and include the following:

- KEY RESOURCES TABLE
- RESOURCE AVAILABILITY
  - ○ Lead contact
  - ○ Materials availability
  - ○ Data and code availability
- EXPERIMENTAL MODEL AND SUBJECT DETAILS
  - ○ Human subjects
- METHOD DETAILS
  - ○ Preparation of NSCLC tissue and normal lung tissue
  - ○ BD Rhapsody library preparation and sequencing
  - ○ Flow cytometry
  - ○ Multiplex immunofluorescence
  - ○ Immunohistochemistry
  - ○ Generation of the core atlas
  - ○ Preprocessing and quality control of scRNA-seq data

- Integration of scRNA-seq datasets
- Doublet-detection
- Unsupervised clustering and cell-type annotation
- Integrating additional datasets
- Comparing cell-type abundances
- Patient stratification
- RNA velocity analysis
- Differential gene expression testing
- Pathway, TF and cytokine signaling signatures
- Cellphonedb analysis
- SCISSOR analysis
- TRN clusters
- TRN signatures
- Signature scoring in bulk RNA-seq samples
- Survival analysis

- QUANTIFICATION AND STATISTICAL ANALYSIS
- ADDITIONAL RESOURCES

SUPPLEMENTAL INFORMATION

Supplemental information can be found online at https://doi.org/10.1016/j.ccell.2022.10.008.

ACKNOWLEDGMENTS

The authors would like to thank Marcus Kalb, Sophia Daum, Annabella Pittl, and Elisabeth Hoflehner for technical support as well as Herbert Mair and Matthias Haas for surgical support and patient recruitment. This work was supported by the European Research Council (grant agreement no. 786295 to Z.T.), by the Austrian Science Fund (FWF) (projects I3978 to Z.T., TAI-687 to D.W., and T 974-B30 to F.F.), and by the Oesterreichische Nationalbank (OeNB) (18496 to F.F.). G.S. was supported by a DOC fellowship from the Austrian Academy of Sciences. Z.T. is a member of the German Research Foundation (DFG) project TRR 241(INF). D.W.'s work was supported by the "Deutsche Krebshilfe" (grant no. DKH 70112994). D.W., S. Sopper, and A.P. were supported by the In Memoriam Gabriel Salzner Stiftung. S. Sopper was supported by the FFG grant Austrian Research Promotion Agency (858057 HD FACS) and L.H. by the OEGHO Förderpreis Onkologie 2021. The results published here are in part based upon data generated by the TCGA Research Network: https://www.cancer.gov/tcga.

AUTHOR CONTRIBUTIONS

Conceptualization, G.S., S. Salcher, L.H., D.W., A.P., and Z.T.; data analysis, G.S., G.F., E.P., F.F., L.H., S. Salcher, S. Sopper, and D.R.; human samples, L.H., G.G., K.S., F.A., G.P., S. Salcher, A.P., and D.W.; single-cell sequencing, S. Salcher, G.U., M.T., and M.S.; sample analysis, S. Salcher, S. Sopper, M.S., and G.U.; orthogonal validation, A.M., C.K., S.P., and K.S.; writing – original draft, L.H., S. Salcher, and Z.T.; writing – review & editing, all authors; funding acquisition, D.W., S. Sopper, A.P., and Z.T.

DECLARATION OF INTERESTS

The authors declare no competing interests.

Received: May 9, 2022
Revised: August 26, 2022
Accepted: October 6, 2022
Published: November 10, 2022

REFERENCES

1. Chen, Z., Fillmore, C.M., Hammerman, P.S., Kim, C.F., and Wong, K.K. (2014). Non-small-cell lung cancers: a heterogeneous set of diseases. Nat. Rev. Cancer *14*, 535–546. https://doi.org/10.1038/nrc3775.
2. Sung, H., Ferlay, J., Siegel, R.L., Laversanne, M., Soerjomataram, I., Jemal, A., and Bray, F. (2021). Global cancer statistics 2020: GLOBOCAN estimates of incidence and mortality worldwide for 36 cancers in 185 countries. CA. Cancer J. Clin. *71*, 209–249. https://doi.org/10.3322/caac.21660.
3. Lambrechts, D., Wauters, E., Boeckx, B., Aibar, S., Nittner, D., Burton, O., Bassez, A., Decaluwé, H., Pircher, A., Van den Eynde, K., et al. (2018). Phenotype molding of stromal cells in the lung tumor microenvironment. Nat. Med. *24*, 1277–1289. https://doi.org/10.1038/s41591-018-0096-5.
4. Zilionis, R., Engblom, C., Pfirschke, C., Savova, V., Zemmour, D., Saatcioglu, H.D., Krishnan, I., Maroni, G., Meyerovitz, C.V., Kerwin, C.M., et al. (2019). Single-cell transcriptomics of human and mouse lung cancers reveals conserved myeloid populations across individuals and species. Immunity *50*, 1317–1334.e10. https://doi.org/10.1016/j.immuni.2019.03.009.
5. Chen, J., Tan, Y., Sun, F., Hou, L., Zhang, C., Ge, T., Yu, H., Wu, C., Zhu, Y., Duan, L., et al. (2020). Single-cell transcriptome and antigen-immunoglobin analysis reveals the diversity of B cells in non-small cell lung cancer. Genome Biol. *21*, 152. https://doi.org/10.1186/s13059-020-02064-6.
6. Goveia, J., Rohlenova, K., Taverna, F., Treps, L., Conradi, L.C., Pircher, A., Geldhof, V., de Rooij, L.P.M.H., Kalucka, J., Sokol, L., et al. (2020). An integrated gene expression landscape profiling approach to identify lung tumor endothelial cell heterogeneity and angiogenic candidates. Cancer Cell *37*, 21–36.e13. https://doi.org/10.1016/j.ccell.2019.12.001.
7. Kim, N., Kim, H.K., Lee, K., Hong, Y., Cho, J.H., Choi, J.W., Lee, J.I., Suh, Y.L., Ku, B.M., Eum, H.H., et al. (2020). Single-cell RNA sequencing demonstrates the molecular and cellular reprogramming of metastatic lung adenocarcinoma. Nat. Commun. *11*, 2285. https://doi.org/10.1038/s41467-020-16164-1.
8. Laughney, A.M., Hu, J., Campbell, N.R., Bakhoum, S.F., Setty, M., Lavallée, V.P., Xie, Y., Masilionis, I., Carr, A.J., Kottapalli, S., et al. (2020). Regenerative lineages and immune-mediated pruning in lung cancer metastasis. Nat. Med. *26*, 259–269. https://doi.org/10.1038/s41591-019-0750-6.
9. Maier, B., Leader, A.M., Chen, S.T., Tung, N., Chang, C., LeBerichel, J., Chudnovskiy, A., Maskey, S., Walker, L., Finnigan, J.P., et al. (2020). A conserved dendritic-cell regulatory program limits antitumour immunity. Nature *580*, 257–262. https://doi.org/10.1038/s41586-020-2134-y.
10. Maynard, A., McCoach, C.E., Rotow, J.K., Harris, L., Haderk, F., Kerr, D.L., Yu, E.A., Schenk, E.L., Tan, W., Zee, A., et al. (2020). Therapy-induced evolution of human lung cancer revealed by single-cell RNA sequencing. Cell *182*, 1232–1251.e22. https://doi.org/10.1016/j.cell.2020.07.017.
11. Leader, A.M., Grout, J.A., Maier, B.B., Nabet, B.Y., Park, M.D., Tabachnikova, A., Chang, C., Walker, L., Lansky, A., Le Berichel, J., et al. (2021). Single-cell analysis of human non-small cell lung cancer lesions refines tumor classification and patient stratification. Cancer Cell *39*, 1594–1609.e12. https://doi.org/10.1016/j.ccell.2021.10.009.
12. Wu, F., Fan, J., He, Y., Xiong, A., Yu, J., Li, Y., Zhang, Y., Zhao, W., Zhou, F., Li, W., et al. (2021). Single-cell profiling of tumor heterogeneity and the microenvironment in advanced non-small cell lung cancer. Nat. Commun. *12*, 2540. https://doi.org/10.1038/s41467-021-22801-0.
13. Xing, X., Yang, F., Huang, Q., Guo, H., Li, J., Qiu, M., Bai, F., and Wang, J. (2021). Decoding the multicellular ecosystem of lung adenocarcinoma manifested as pulmonary subsolid nodules by single-cell RNA sequencing. Sci. Adv. *7*, eabd9738. https://doi.org/10.1126/sciadv.abd9738.
14. Stankovic, B., Bjørhovde, H.A.K., Skarshaug, R., Aamodt, H., Frafjord, A., Müller, E., Hammarström, C., Beraki, K., Bækkevold, E.S., Woldbæk, P.R., et al. (2018). Immune cell composition in human non-small cell lung cancer. Front. Immunol. *9*, 3101. https://doi.org/10.3389/fimmu.2018.03101.
15. Guo, X., Zhang, Y., Zheng, L., Zheng, C., Song, J., Zhang, Q., Kang, B., Liu, Z., Jin, L., Xing, R., et al. (2018). Global characterization of T cells in

CellPress
OPEN ACCESS

non-small-cell lung cancer by single-cell sequencing. Nat. Med. *24*, 978–985. https://doi.org/10.1038/s41591-018-0045-3.

16. Eruslanov, E.B., Bhojnagarwala, P.S., Quatromoni, J.G., Stephen, T.L., Ranganathan, A., Deshpande, C., Akimova, T., Vachani, A., Litzky, L., Hancock, W.W., et al. (2014). Tumor-associated neutrophils stimulate T cell responses in early-stage human lung cancer. J. Clin. Invest. *124*, 5466–5480. https://doi.org/10.1172/JCI77053.
17. Kargl, J., Busch, S.E., Yang, G.H.Y., Kim, K.H., Hanke, M.L., Metz, H.E., Hubbard, J.J., Lee, S.M., Madtes, D.K., McIntosh, M.W., and Houghton, A.M. (2017). Neutrophils dominate the immune cell composition in non-small cell lung cancer. Nat. Commun. *8*, 14381. https://doi.org/10.1038/ncomms14381.
18. Shaul, M.E., and Fridlender, Z.G. (2019). Tumour-associated neutrophils in patients with cancer. Nat. Rev. Clin. Oncol. *16*, 601–620. https://doi.org/10.1038/s41571-019-0222-4.
19. Kargl, J., Zhu, X., Zhang, H., Yang, G.H.Y., Friesen, T.J., Shipley, M., Maeda, D.Y., Zebala, J.A., McKay-Fleisch, J., Meredith, G., et al. (2019). Neutrophil content predicts lymphocyte depletion and anti-PD1 treatment failure in NSCLC. JCI Insight *4*, 130850. https://doi.org/10.1172/jci.insight.130850.
20. Templeton, A.J., McNamara, M.G., Šeruga, B., Vera-Badillo, F.E., Aneja, P., Ocaña, A., Leibowitz-Amit, R., Sonpavde, G., Knox, J.J., Tran, B., et al. (2014). Prognostic role of neutrophil-to-lymphocyte ratio in solid tumors: a systematic review and meta-analysis. J. Natl. Cancer Inst. *106*, dju124. https://doi.org/10.1093/jnci/dju124.
21. Peng, B., Wang, Y.H., Liu, Y.M., and Ma, L.X. (2015). Prognostic significance of the neutrophil to lymphocyte ratio in patients with non-small cell lung cancer: a systemic review and meta-analysis. Int. J. Clin. Exp. Med. 8, 3098–3106.
22. Oliveira, G., Stromhaug, K., Klaeger, S., Kula, T., Frederick, D.T., Le, P.M., Forman, J., Huang, T., Li, S., Zhang, W., et al. (2021). Phenotype, specificity and avidity of antitumour CD8(+) T cells in melanoma. Nature *596*, 119–125. https://doi.org/10.1038/s41586-021-03704-y.
23. He, X., Wang, L., Riedel, H., Wang, K., Yang, Y., Dinu, C.Z., and Rojanasakul, Y. (2017). Mesothelin promotes epithelial-to-mesenchymal transition and tumorigenicity of human lung cancer and mesothelioma cells. Mol. Cancer *16*, 63. https://doi.org/10.1186/s12943-017-0633-8.
24. Francart, M.E., Vanwynsberghe, A.M., Lambert, J., Bourcy, M., Genna, A., Ancel, J., Perez-Boza, J., Noël, A., Birembaut, P., Struman, I., et al. (2020). Vimentin prevents a miR-dependent negative regulation of tissue factor mRNA during epithelial-mesenchymal transitions and facilitates early metastasis. Oncogene *39*, 3680–3692. https://doi.org/10.1038/s41388-020-1244-1.
25. Kubala, M.H., Punj, V., Placencio-Hickok, V.R., Fang, H., Fernandez, G.E., Sposto, R., and DeClerck, Y.A. (2018). Plasminogen activator inhibitor-1 promotes the recruitment and polarization of macrophages in cancer. Cell Rep. *25*, 2177–2191.e7. https://doi.org/10.1016/j.celrep.2018.10.082.
26. Ji, P., Diederichs, S., Wang, W., Böing, S., Metzger, R., Schneider, P.M., Tidow, N., Brandt, B., Buerger, H., Bulk, E., et al. (2003). MALAT-1, a novel noncoding RNA, and thymosin beta4 predict metastasis and survival in early-stage non-small cell lung cancer. Oncogene *22*, 8031–8041. https://doi.org/10.1038/sj.onc.1206928.
27. Qi, L., Liu, F., Zhang, F., Zhang, S., Lv, L., Bi, Y., and Yu, Y. (2018). lncRNA NEAT1 competes against let-7a to contribute to non-small cell lung cancer proliferation and metastasis. Biomed. Pharmacother. *103*, 1507–1515. https://doi.org/10.1016/j.biopha.2018.04.053.
28. Wang, L., Liu, Y., Dai, Y., Tang, X., Yin, T., Wang, C., Wang, T., Dong, L., Shi, M., Qin, J., et al. (2022). Single-cell RNA-seq analysis reveals BHLHE40-driven pro-tumour neutrophils with hyperactivated glycolysis in pancreatic tumour microenvironment. Gut. https://doi.org/10.1136/gutjnl-2021-326070.
29. Lotfollahi, M., Naghipourfar, M., Luecken, M.D., Khajavi, M., Büttner, M., Wagenstetter, M., Avsec, Ž., Gayoso, A., Yosef, N., Interlandi, M., et al. (2022). Mapping single-cell data to reference atlases by transfer learning. Nat. Biotechnol. *40*, 121–130. https://doi.org/10.1038/s41587-021-01001-7.
30. Büttner, M., Ostner, J., Müller, C.L., Theis, F.J., and Schubert, B. (2021). scCODA is a Bayesian model for compositional single-cell data analysis. Nat. Commun. *12*, 6876. https://doi.org/10.1038/s41467-021-27150-6.
31. Schubert, M., Klinger, B., Klünemann, M., Sieber, A., Uhlitz, F., Sauer, S., Garnett, M.J., Blüthgen, N., and Saez-Rodriguez, J. (2018). Perturbation-response genes reveal signaling footprints in cancer gene expression. Nat. Commun. *9*, 20. https://doi.org/10.1038/s41467-017-02391-6.
32. Jiang, G., Shi, L., Zheng, X., Zhang, X., Wu, K., Liu, B., Yan, P., Liang, X., Yu, T., Wang, Y., and Cai, X. (2020). Androgen receptor affects the response to immune checkpoint therapy by suppressing PD-L1 in hepatocellular carcinoma. Aging *12*, 11466–11484. https://doi.org/10.18632/aging.103231.
33. Garcia-Alonso, L., Holland, C.H., Ibrahim, M.M., Turei, D., and Saez-Rodriguez, J. (2019). Benchmark and integration of resources for the estimation of human transcription factor activities. Genome Res. *29*, 1363–1375. https://doi.org/10.1101/gr.240663.118.
34. Holland, C.H., Tanevski, J., Perales-Patón, J., Gleixner, J., Kumar, M.P., Mereu, E., Joughin, B.A., Stegle, O., Lauffenburger, D.A., Heyn, H., et al. (2020). Robustness and applicability of transcription factor and pathway analysis tools on single-cell RNA-seq data. Genome Biol. *21*, 36. https://doi.org/10.1186/s13059-020-1949-z.
35. Peng, S.L. (2008). Foxo in the immune system. Oncogene *27*, 2337–2344. https://doi.org/10.1038/onc.2008.26.
36. Jiang, P., Zhang, Y., Ru, B., Yang, Y., Vu, T., Paul, R., Mirza, A., Altan-Bonnet, G., Liu, L., Ruppin, E., et al. (2021). Systematic investigation of cytokine signaling activity at the tissue and single-cell levels. Nat. Methods *18*, 1181–1191. https://doi.org/10.1038/s41592-021-01274-5.
37. Yang, W.C., Hwang, Y.S., Chen, Y.Y., Liu, C.L., Shen, C.N., Hong, W.H., Lo, S.M., and Shen, C.R. (2017). Interleukin-4 supports the suppressive immune responses elicited by regulatory T cells. Front. Immunol. *8*, 1508. https://doi.org/10.3389/fimmu.2017.01508.
38. Motz, G.T., and Coukos, G. (2011). The parallel lives of angiogenesis and immunosuppression: cancer and other tales. Nat. Rev. Immunol. *11*, 702–711. https://doi.org/10.1038/nri3064.
39. La Fleur, L., Botling, J., He, F., Pelicano, C., Zhou, C., He, C., Palano, G., Mezheyeuski, A., Micke, P., Ravetch, J.V., et al. (2021). Targeting MARCO and IL37R on immunosuppressive macrophages in lung cancer blocks regulatory T cells and supports cytotoxic lymphocyte function. Cancer Res. *81*, 956–967. https://doi.org/10.1158/0008-5472.CAN-20-1885.
40. Yin, Y., Xu, X., Tang, J., Zhang, W., Zhangyuan, G., Ji, J., Deng, L., Lu, S., Zhuo, H., and Sun, B. (2018). CD97 promotes tumor aggressiveness through the traditional G protein-coupled receptor-mediated signaling in hepatocellular carcinoma. Hepatology *68*, 1865–1878. https://doi.org/10.1002/hep.30068.
41. Shojaei, F., Scott, N., Kang, X., Lappin, P.B., Fitzgerald, A.A., Karlicek, S., Simmons, B.H., Wu, A., Lee, J.H., Bergqvist, S., and Kraynov, E. (2012). Osteopontin induces growth of metastatic tumors in a preclinical model of non-small lung cancer. J. Exp. Clin. Cancer Res. *31*, 26. https://doi.org/10.1186/1756-9966-31-26.
42. Zhang, J., Takahashi, K., Takahashi, F., Shimizu, K., Ohshita, F., Kameda, Y., Maeda, K., Nishio, K., and Fukuchi, Y. (2001). Differential osteopontin expression in lung cancer. Cancer Lett. *171*, 215–222. https://doi.org/10.1016/s0304-3835(01)00607-3.
43. Meng, J., Jiang, Y.Z., Zhao, S., Tao, Y., Zhang, T., Wang, X., Zhang, Y., Sun, K., Yuan, M., Chen, J., et al. (2022). Tumor-derived Jagged1 promotes cancer progression through immune evasion. Cell Rep. *38*, 110492. https://doi.org/10.1016/j.celrep.2022.110492.
44. Mikucki, M.E., Fisher, D.T., Matsuzaki, J., Skitzki, J.J., Gaulin, N.B., Muhitch, J.B., Ku, A.W., Frelinger, J.G., Odunsi, K., Gajewski, T.F., et al. (2015). Non-redundant requirement for CXCR3 signalling during tumoricidal T-cell trafficking across tumour vascular checkpoints. Nat. Commun. *6*, 7458. https://doi.org/10.1038/ncomms8458.

45. Choi, H.Y., Jo, H., Zhao, X., Hoadley, K.A., Newman, S., Holt, J., Hayward, M.C., Love, M.I., Marron, J.S., and Hayes, D.N. (2021). SCISSOR: a framework for identifying structural changes in RNA transcripts. Nat. Commun. *12*, 286. https://doi.org/10.1038/s41467-020-20593-3.

46. Charoentong, P., Finotello, F., Angelova, M., Mayer, C., Efremova, M., Rieder, D., Hackl, H., and Trajanoski, Z. (2017). Pan-cancer immunogenomic analyses reveal genotype-immunophenotype relationships and predictors of response to checkpoint blockade. Cell Rep. *18*, 248–262. https://doi.org/10.1016/j.celrep.2016.12.019.

47. Biton, J., Mansuet-Lupo, A., Pécuchet, N., Alifano, M., Ouakrim, H., Arrondeau, J., Boudou-Rouquette, P., Goldwasser, F., Leroy, K., Goc, J., et al. (2018). TP53, STK11, and EGFR mutations predict tumor immune profile and the response to anti-PD-1 in lung adenocarcinoma. Clin. Cancer Res. *24*, 5710–5723. https://doi.org/10.1158/1078-0432.CCR-18-0163.

48. Wellenstein, M.D., and de Visser, K.E. (2018). Cancer-cell-intrinsic mechanisms shaping the tumor immune landscape. Immunity *48*, 399–416. https://doi.org/10.1016/j.immuni.2018.03.004.

49. Patel, A.J., Richter, A., Drayson, M.T., and Middleton, G.W. (2020). The role of B lymphocytes in the immuno-biology of non-small-cell lung cancer. Cancer Immunol. Immunother. *69*, 325–342. https://doi.org/10.1007/s00262-019-02461-2.

50. Jaillon, S., Ponzetta, A., Di Mitri, D., Santoni, A., Bonecchi, R., and Mantovani, A. (2020). Neutrophil diversity and plasticity in tumour progression and therapy. Nat. Rev. Cancer *20*, 485–503. https://doi.org/10.1038/s41568-020-0281-y.

51. Ballesteros, I., Rubio-Ponce, A., Genua, M., Lusito, E., Kwok, I., Fernández-Calvo, G., Khoyratty, T.E., van Grinsven, E., González-Hernández, S., Nicolás-Ávila, J.Á., et al. (2020). Co-Option of neutrophil fates by tissue environments. Cell *183*, 1282–1297.e18. https://doi.org/10.1016/j.cell.2020.10.003.

52. Hedrick, C.C., and Malanchi, I. (2022). Neutrophils in cancer: heterogeneous and multifaceted. Nat. Rev. Immunol. *22*, 173–187. https://doi.org/10.1038/s41577-021-00571-6.

53. Martin, C., Burdon, P.C.E., Bridger, G., Gutierrez-Ramos, J.C., Williams, T.J., and Rankin, S.M. (2003). Chemokines acting via CXCR2 and CXCR4 control the release of neutrophils from the bone marrow and their return following senescence. Immunity *19*, 583–593. https://doi.org/10.1016/s1074-7613(03)00263-2.

54. Coffelt, S.B., Wellenstein, M.D., and de Visser, K.E. (2016). Neutrophils in cancer: neutral no more. Nat. Rev. Cancer *16*, 431–446. https://doi.org/10.1038/nrc.2016.52.

55. Evrard, M., Kwok, I.W.H., Chong, S.Z., Teng, K.W.W., Becht, E., Chen, J., Sieow, J.L., Penny, H.L., Ching, G.C., Devi, S., et al. (2018). Developmental analysis of bone marrow neutrophils reveals populations specialized in expansion, trafficking, and effector functions. Immunity *48*, 364–379.e8. https://doi.org/10.1016/j.immuni.2018.02.002.

56. Schulte-Schrepping, J., Reusch, N., Paclik, D., Baßler, K., Schlickeiser, S., Zhang, B., Krämer, B., Krammer, T., Brumhard, S., Bonaguro, L., et al. (2020). Severe COVID-19 is marked by a dysregulated myeloid cell compartment. Cell *182*, 1419–1440.e23. https://doi.org/10.1016/j.cell.2020.08.001.

57. Lin, R., Zhang, Y., Pradhan, K., and Li, L. (2020). TICAM2-related pathway mediates neutrophil exhaustion. Sci. Rep. *10*, 14397. https://doi.org/10.1038/s41598-020-71379-y.

58. Yamashiro, S., Wang, J.M., Yang, D., Gong, W.H., Kamohara, H., and Yoshimura, T. (2000). Expression of CCR6 and CD83 by cytokine-activated human neutrophils. Blood *96*, 3958–3963.

59. Li, Z., Ju, X., Silveira, P.A., Abadir, E., Hsu, W.H., Hart, D.N.J., and Clark, G.J. (2019). CD83: activation marker for antigen presenting cells and its therapeutic potential. Front. Immunol. *10*, 1312. https://doi.org/10.3389/fimmu.2019.01312.

60. Del Prete, A., Martínez-Muñoz, L., Mazzon, C., Toffali, L., Sozio, F., Za, L., Bosisio, D., Gazzurelli, L., Salvi, V., Tiberio, L., et al. (2017). The atypical receptor CCRL2 is required for CXCR2-dependent neutrophil recruitment and tissue damage. Blood *130*, 1223–1234. https://doi.org/10.1182/blood-2017-04-777680.

61. Clayton, S.A., Daley, K.K., MacDonald, L., Fernandez-Vizarra, E., Bottegoni, G., O'Neil, J.D., Major, T., Griffin, D., Zhuang, Q., Adewoye, A.B., et al. (2021). Inflammation causes remodeling of mitochondrial cytochrome c oxidase mediated by the bifunctional gene C15orf48. Sci. Adv. *7*, eabl5182. https://doi.org/10.1126/sciadv.abl5182.

62. Zhang, D., Chen, G., Manwani, D., Mortha, A., Xu, C., Faith, J.J., Burk, R.D., Kunisaki, Y., Jang, J.E., Scheiermann, C., et al. (2015). Neutrophil ageing is regulated by the microbiome. Nature *525*, 528–532. https://doi.org/10.1038/nature15367.

63. Ozel, I., Duerig, I., Domnich, M., Lang, S., Pylaeva, E., and Jablonska, J. (2022). The good, the bad, and the ugly: neutrophils, angiogenesis, and cancer. Cancers *14*, 536. https://doi.org/10.3390/cancers14030536.

64. González-Chavarría, I., Cerro, R.P., Parra, N.P., Sandoval, F.A., Zuñiga, F.A., Omazábal, V.A., Lamperti, L.I., Jiménez, S.P., Fernandez, E.A., Gutiérrez, N.A., et al. (2014). Lectin-like oxidized LDL receptor-1 is an enhancer of tumor angiogenesis in human prostate cancer cells. PLoS One *9*, e106219. https://doi.org/10.1371/journal.pone.0106219.

65. Condamine, T., Dominguez, G.A., Youn, J.I., Kossenkov, A.V., Mony, S., Alicea-Torres, K., Tcyganov, E., Hashimoto, A., Nefedova, Y., Lin, C., et al. (2016). Lectin-type oxidized LDL receptor-1 distinguishes population of human polymorphonuclear myeloid-derived suppressor cells in cancer patients. Sci. Immunol. *1*, aaf8943. https://doi.org/10.1126/sciimmunol.aaf8943.

66. Chui, P.C., Guan, H.P., Lehrke, M., and Lazar, M.A. (2005). PPARgamma regulates adipocyte cholesterol metabolism via oxidized LDL receptor 1. J. Clin. Invest. *115*, 2244–2256. https://doi.org/10.1172/JCI24130.

67. Steele, C.W., Karim, S.A., Leach, J.D.G., Bailey, P., Upstill-Goddard, R., Rishi, L., Foth, M., Bryson, S., McDaid, K., Wilson, Z., et al. (2016). CXCR2 inhibition profoundly suppresses metastases and augments immunotherapy in pancreatic ductal adenocarcinoma. Cancer Cell *29*, 832–845. https://doi.org/10.1016/j.ccell.2016.04.014.

68. Meijer, B., Gearry, R.B., and Day, A.S. (2012). The role of S100A12 as a systemic marker of inflammation. Int. J. Inflam. *2012*, 907078. https://doi.org/10.1155/2012/907078.

69. Leshner, M., Wang, S., Lewis, C., Zheng, H., Chen, X.A., Santy, L., and Wang, Y. (2012). PAD4 mediated histone hypercitrullination induces heterochromatin decondensation and chromatin unfolding to form neutrophil extracellular trap-like structures. Front. Immunol. *3*, 307. https://doi.org/10.3389/fimmu.2012.00307.

70. Hattar, K., Fink, L., Fietzner, K., Himmel, B., Grimminger, F., Seeger, W., and Sibelius, U. (2001). Cell density regulates neutrophil IL-8 synthesis: role of IL-1 receptor antagonist and soluble TNF receptors. J. Immunol. *166*, 6287–6293. https://doi.org/10.4049/jimmunol.166.10.6287.

71. Yang, Q., Tian, S., Liu, Z., and Dong, W. (2021). Knockdown of RIPK2 inhibits proliferation and migration, and induces apoptosis via the NF-kappaB signaling pathway in gastric cancer. Front. Genet. *12*, 627464. https://doi.org/10.3389/fgene.2021.627464.

72. Katayama, Y., Hidalgo, A., Chang, J., Peired, A., and Frenette, P.S. (2005). CD44 is a physiological E-selectin ligand on neutrophils. J. Exp. Med. *201*, 1183–1189. https://doi.org/10.1084/jem.20042014.

73. Wolf, F.A., Hamey, F.K., Plass, M., Solana, J., Dahlin, J.S., Göttgens, B., Rajewsky, N., Simon, L., and Theis, F.J. (2019). PAGA: graph abstraction reconciles clustering with trajectory inference through a topology preserving map of single cells. Genome Biol. *20*, 59. https://doi.org/10.1186/s13059-019-1663-x.

74. Zheng, Y., Hao, S., Xiang, C., Han, Y., Shang, Y., Zhen, Q., Zhao, Y., Zhang, M., and Zhang, Y. (2021). The correlation between SPP1 and immune escape of EGFR mutant lung adenocarcinoma was explored by bioinformatics analysis. Front. Oncol. *11*, 592854. https://doi.org/10.3389/fonc.2021.592854.

75. Shojaei, F., Scott, N., Kang, X., Lappin, P.B., Fitzgerald, A.A., Karlicek, S., Simmons, B.H., Wu, A., Lee, J.H., Bergqvist, S., and Kraynov, E. (2012).

Osteopontin induces growth of metastatic tumors in a preclinical model of non-small lung cancer. J. Exp. Clin. Cancer Res. *31*, 26. https://doi.org/10.1186/1756-9966-31-26.

76. Afonso, P.V., Janka-Junttila, M., Lee, Y.J., McCann, C.P., Oliver, C.M., Aamer, K.A., Losert, W., Cicerone, M.T., and Parent, C.A. (2012). LTB4 is a signal-relay molecule during neutrophil chemotaxis. Dev. Cell *22*, 1079–1091. https://doi.org/10.1016/j.devcel.2012.02.003.

77. Besteman, S.B., Callaghan, A., Langedijk, A.C., Hennus, M.P., Meyaard, L., Mokry, M., Bont, L.J., and Calis, J.J.A. (2020). Transcriptome of airway neutrophils reveals an interferon response in life-threatening respiratory syncytial virus infection. Clin. Immunol. *220*, 108593. https://doi.org/10.1016/j.clim.2020.108593.

78. Becht, E., Giraldo, N.A., Lacroix, L., Buttard, B., Elarouci, N., Petitprez, F., Selves, J., Laurent-Puig, P., Sautès-Fridman, C., Fridman, W.H., and de Reyniès, A. (2016). Estimating the population abundance of tissue-infiltrating immune and stromal cell populations using gene expression. Genome Biol. *17*, 218. https://doi.org/10.1186/s13059-016-1070-5.

79. Fehrenbacher, L., Spira, A., Ballinger, M., Kowanetz, M., Vansteenkiste, J., Mazieres, J., Park, K., Smith, D., Artal-Cortes, A., Lewanski, C., et al. (2016). Atezolizumab versus docetaxel for patients with previously treated non-small-cell lung cancer (POPLAR): a multicentre, open-label, phase 2 randomised controlled trial. Lancet *387*, 1837–1846. https://doi.org/10.1016/S0140-6736(16)00587-0.

80. Rittmeyer, A., Barlesi, F., Waterkamp, D., Park, K., Ciardiello, F., von Pawel, J., Gadgeel, S.M., Hida, T., Kowalski, D.M., Dols, M.C., et al. (2017). Atezolizumab versus docetaxel in patients with previously treated non-small-cell lung cancer (OAK): a phase 3, open-label, multicentre randomised controlled trial. Lancet *389*, 255–265. https://doi.org/10.1016/S0140-6736(16)32517-X.

81. Patil, N.S., Nabet, B.Y., Müller, S., Koeppen, H., Zou, W., Giltnane, J., Au-Yeung, A., Srivats, S., Cheng, J.H., Takahashi, C., et al. (2022). Intratumoral plasma cells predict outcomes to PD-L1 blockade in non-small cell lung cancer. Cancer Cell *40*, 289–300.e4. https://doi.org/10.1016/j.ccell.2022.02.002.

82. Li, T., Li, X., Zamani, A., Wang, W., Lee, C.N., Li, M., Luo, G., Eiler, E., Sun, H., Ghosh, S., et al. (2020). c-Rel is a myeloid checkpoint for cancer immunotherapy. Nat. Cancer *1*, 507–517. https://doi.org/10.1038/s43018-020-0061-3.

83. Fridman, W.H., Meylan, M., Petitprez, F., Sun, C.M., Italiano, A., and Sautès-Fridman, C. (2022). B cells and tertiary lymphoid structures as determinants of tumour immune contexture and clinical outcome. Nat. Rev. Clin. Oncol. *19*, 441–457. https://doi.org/10.1038/s41571-022-00619-z.

84. Dong, Z.Y., Zhang, J.T., Liu, S.Y., Su, J., Zhang, C., Xie, Z., Zhou, Q., Tu, H.Y., Xu, C.R., Yan, L.X., et al. (2017). EGFR mutation correlates with uninflamed phenotype and weak immunogenicity, causing impaired response to PD-1 blockade in non-small cell lung cancer. OncoImmunology *6*, e1356145. https://doi.org/10.1080/2162402x.2017.1356145.

85. Pinto, R., Petriella, D., Lacalamita, R., Montrone, M., Catino, A., Pizzutilo, P., Botticella, M.A., Zito, F.A., Del Bene, G., Zonno, A., et al. (2019). KRAS-driven lung adenocarcinoma and B cell infiltration: novel insights for immunotherapy. Cancers *11*, E1145. https://doi.org/10.3390/cancers11081145.

86. Saha, S., and Biswas, S.K. (2016). Tumor-associated neutrophils show phenotypic and functional divergence in human lung cancer. Cancer Cell *30*, 11–13. https://doi.org/10.1016/j.ccell.2016.06.016.

87. Mysore, V., Cullere, X., Mears, J., Rosetti, F., Okubo, K., Liew, P.X., Zhang, F., Madera-Salcedo, I., Rosenbauer, F., Stone, R.M., et al. (2021). FcgammaR engagement reprograms neutrophils into antigen cross-presenting cells that elicit acquired anti-tumor immunity. Nat. Commun. *12*, 4791. https://doi.org/10.1038/s41467-021-24591-x.

88. Zhang, H., and Houghton, A.M. (2021). Good cops turn bad: the contribution of neutrophils to immune-checkpoint inhibitor treatment failures in cancer. Pharmacol. Ther. *217*, 107662. https://doi.org/10.1016/j.pharmthera.2020.107662.

89. Rørvig, S., Østergaard, O., Heegaard, N.H.H., and Borregaard, N. (2013). Proteome profiling of human neutrophil granule subsets, secretory vesicles, and cell membrane: correlation with transcriptome profiling of neutrophil precursors. J. Leukoc. Biol. *94*, 711–721. https://doi.org/10.1189/jlb.1212619.

90. Mayr, C.H., Simon, L.M., Leuschner, G., Ansari, M., Schniering, J., Geyer, P.E., Angelidis, I., Strunz, M., Singh, P., Kneidinger, N., et al. (2021). Integrative analysis of cell state changes in lung fibrosis with peripheral protein biomarkers. EMBO Mol. Med. *13*, e12871. https://doi.org/10.15252/emmm.202012871.

91. He, D., Wang, D., Lu, P., Yang, N., Xue, Z., Zhu, X., Zhang, P., and Fan, G. (2021). Single-cell RNA sequencing reveals heterogeneous tumor and immune cell populations in early-stage lung adenocarcinomas harboring EGFR mutations. Oncogene *40*, 355–368. https://doi.org/10.1038/s41388-020-01528-0.

92. Madissoon, E., Wilbrey-Clark, A., Miragaia, R.J., Saeb-Parsy, K., Mahbubani, K.T., Georgakopoulos, N., Harding, P., Polanski, K., Huang, N., Nowicki-Osuch, K., et al. (2019). scRNA-seq assessment of the human lung, spleen, and esophagus tissue stability after cold preservation. Genome Biol. *21*, 1. https://doi.org/10.1186/s13059-019-1906-x.

93. Reyfman, P.A., Walter, J.M., Joshi, N., Anekalla, K.R., McQuattie-Pimentel, A.C., Chiu, S., Fernandez, R., Akbarpour, M., Chen, C.I., Ren, Z., et al. (2019). Single-cell transcriptomic analysis of human lung provides insights into the pathobiology of pulmonary fibrosis. Am. J. Respir. Crit. Care Med. *199*, 1517–1536. https://doi.org/10.1164/rccm.201712-2410OC.

94. Vieira Braga, F.A., Kar, G., Berg, M., Carpaij, O.A., Polanski, K., Simon, L.M., Brouwer, S., Gomes, T., Hesse, L., Jiang, J., et al. (2019). A cellular census of human lungs identifies novel cell states in health and in asthma. Nat. Med. *25*, 1153–1163. https://doi.org/10.1038/s41591-019-0468-5.

95. Adams, T.S., Schupp, J.C., Poli, S., Ayaub, E.A., Neumark, N., Ahangari, F., Chu, S.G., Raby, B.A., DeIuliis, G., Januszyk, M., et al. (2020). Single-cell RNA-seq reveals ectopic and aberrant lung-resident cell populations in idiopathic pulmonary fibrosis. Sci. Adv. *6*, eaba1983. https://doi.org/10.1126/sciadv.aba1983.

96. Habermann, A.C., Gutierrez, A.J., Bui, L.T., Yahn, S.L., Winters, N.I., Calvi, C.L., Peter, L., Chung, M.I., Taylor, C.J., Jetter, C., et al. (2020). Single-cell RNA sequencing reveals profibrotic roles of distinct epithelial and mesenchymal lineages in pulmonary fibrosis. Sci. Adv. *6*, eaba1972. https://doi.org/10.1126/sciadv.aba1972.

97. Travaglini, K.J., Nabhan, A.N., Penland, L., Sinha, R., Gillich, A., Sit, R.V., Chang, S., Conley, S.D., Mori, Y., Seita, J., et al. (2020). A molecular cell atlas of the human lung from single-cell RNA sequencing. Nature *587*, 619–625. https://doi.org/10.1038/s41586-020-2922-4.

98. Picelli, S., Björklund, Å.K., Faridani, O.R., Sagasser, S., Winberg, G., and Sandberg, R. (2013). Smart-seq2 for sensitive full-length transcriptome profiling in single cells. Nat. Methods *10*, 1096–1098. https://doi.org/10.1038/nmeth.2639.

99. Klein, A.M., Mazutis, L., Akartuna, I., Tallapragada, N., Veres, A., Li, V., Peshkin, L., Weitz, D.A., and Kirschner, M.W. (2015). Droplet barcoding for single-cell transcriptomics applied to embryonic stem cells. Cell *161*, 1187–1201. https://doi.org/10.1016/j.cell.2015.04.044.

100. Macosko, E.Z., Basu, A., Satija, R., Nemesh, J., Shekhar, K., Goldman, M., Tirosh, I., Bialas, A.R., Kamitaki, N., Martersteck, E.M., et al. (2015). Highly parallel genome-wide expression profiling of individual cells using nanoliter droplets. Cell *161*, 1202–1214. https://doi.org/10.1016/j.cell.2015.05.002.

101. Ewels, P.A., Peltzer, A., Fillinger, S., Patel, H., Alneberg, J., Wilm, A., Garcia, M.U., Di Tommaso, P., and Nahnsen, S. (2020). The nf-core framework for community-curated bioinformatics pipelines. Nat. Biotechnol. *38*, 276–278. https://doi.org/10.1038/s41587-020-0439-x.

102. Patel, H., Ewels, P., Peltzer, A., Hammarén, R., Botvinnik, O., Sturm, G., Moreno, D., Vemuri, P., silviamorins, P.L., et al. (2022). Nf-Core/Rnaseq: Nf-Core/rnaseq v3.6 - Platinum Platypus (Zenodo).

103. Virshup, I., Rybakov, S., Theis, F.J., Angerer, P., and Wolf, F.A. (2021). anndata: annotated data. Preprint at bioRxiv. https://doi.org/10.1101/2021.12.16.473007.

104. Wolf, F.A., Angerer, P., and Theis, F.J. (2018). SCANPY: large-scale single-cell gene expression data analysis. Genome Biol. *19*, 15. https://doi.org/10.1186/s13059-017-1382-0.

105. Xin, J., Mark, A., Afrasiabi, C., Tsueng, G., Juchler, M., Gopal, N., Stupp, G.S., Putman, T.E., Ainscough, B.J., Griffith, O.L., et al. (2016). High-performance web services for querying gene and variant annotation. Genome Biol. *17*, 91. https://doi.org/10.1186/s13059-016-0953-9.

106. Xu, C., Lopez, R., Mehlman, E., Regier, J., Jordan, M.I., and Yosef, N. (2021). Probabilistic harmonization and annotation of single-cell transcriptomics data with deep generative models. Mol. Syst. Biol. *17*, e9620. https://doi.org/10.15252/msb.20209620.

107. Gayoso, A., Lopez, R., Xing, G., Boyeau, P., Valiollah Pour Amiri, V., Hong, J., Wu, K., Jayasuriya, M., Mehlman, E., Langevin, M., et al. (2022). A Python library for probabilistic analysis of single-cell omics data. Nat. Biotechnol. *40*, 163–166. https://doi.org/10.1038/s41587-021-01206-w.

108. Luecken, M.D., Büttner, M., Chaichoompu, K., Danese, A., Interlandi, M., Mueller, M.F., Strobl, D.C., Zappia, L., Dugas, M., Colomé-Tatché, M., and Theis, F.J. (2022). Benchmarking atlas-level data integration in single-cell genomics. Nat. Methods *19*, 41–50. https://doi.org/10.1038/s41592-021-01336-8.

109. Lopez, R., Regier, J., Cole, M.B., Jordan, M.I., and Yosef, N. (2018). Deep generative modeling for single-cell transcriptomics. Nat. Methods *15*, 1053–1058. https://doi.org/10.1038/s41592-018-0229-2.

110. Bernstein, N.J., Fong, N.L., Lam, I., Roy, M.A., Hendrickson, D.G., and Kelley, D.R. (2020). Solo: doublet identification in single-cell RNA-seq via semi-supervised deep learning. Cell Syst. *11*, 95–101.e5. https://doi.org/10.1016/j.cels.2020.05.010.

111. Xi, N.M., and Li, J.J. (2021). Benchmarking computational doublet-detection methods for single-cell RNA sequencing data. Cell Syst. *12*, 176–194.e6. https://doi.org/10.1016/j.cels.2020.11.008.

112. Becht, E., McInnes, L., Healy, J., Dutertre, C.A., Kwok, I.W.H., Ng, L.G., Ginhoux, F., and Newell, E.W. (2018). Dimensionality reduction for visualizing single-cell data using UMAP. Nat. Biotechnol. *37*, 38–44. https://doi.org/10.1038/nbt.4314.

113. Traag, V.A., Waltman, L., and van Eck, N.J. (2019). From Louvain to Leiden: guaranteeing well-connected communities. Sci. Rep. *9*, 5233. https://doi.org/10.1038/s41598-019-41695-z.

114. Schupp, J.C., Adams, T.S., Cosme, C., Jr., Raredon, M.S.B., Yuan, Y., Omote, N., Poli, S., Chioccioli, M., Rose, K.A., Manning, E.P., et al. (2021). Integrated single-cell atlas of endothelial cells of the human lung. Circulation *144*, 286–302. https://doi.org/10.1161/CIRCULATIONAHA.120.052318.

115. Sikkema, L., Strobl, D., Zappia, L., Madissoon, E., Markov, N.S., Zaragosi, L., Ansari, M., Arguel, M., Apperloo, L., Bécavin, C., et al. (2022). An integrated cell atlas of the human lung in health and disease. Preprint at bioRxiv. https://doi.org/10.1101/2022.03.10.483747.

116. La Manno, G., Soldatov, R., Zeisel, A., Braun, E., Hochgerner, H., Petukhov, V., Lidschreiber, K., Kastriti, M.E., Lönnerberg, P., Furlan, A., et al. (2018). RNA velocity of single cells. Nature *560*, 494–498. https://doi.org/10.1038/s41586-018-0414-6.

117. Bergen, V., Lange, M., Peidli, S., Wolf, F.A., and Theis, F.J. (2020). Generalizing RNA velocity to transient cell states through dynamical modeling. Nat. Biotechnol. *38*, 1408–1414. https://doi.org/10.1038/s41587-020-0591-3.

118. Danecek, P., Bonfield, J.K., Liddle, J., Marshall, J., Ohan, V., Pollard, M.O., Whitwham, A., Keane, T., McCarthy, S.A., Davies, R.M., and Li, H. (2021). Twelve years of SAMtools and BCFtools. GigaScience *10*, giab008. https://doi.org/10.1093/gigascience/giab008.

119. Love, M.I., Huber, W., and Anders, S. (2014). Moderated estimation of fold change and dispersion for RNA-seq data with DESeq2. Genome Biol. *15*, 550. https://doi.org/10.1186/s13059-014-0550-8.

120. Squair, J.W., Gautier, M., Kathe, C., Anderson, M.A., James, N.D., Hutson, T.H., Hudelle, R., Qaiser, T., Matson, K.J.E., Barraud, Q., et al. (2021). Confronting false discoveries in single-cell differential expression. Nat. Commun. *12*, 5692. https://doi.org/10.1038/s41467-021-25960-2.

121. Ignatiadis, N., Klaus, B., Zaugg, J.B., and Huber, W. (2016). Data-driven hypothesis weighting increases detection power in genome-scale multiple testing. Nat. Methods *13*, 577–580. https://doi.org/10.1038/nmeth.3885.

122. Seabold, S., and Perktold, J. (2010). Statsmodels: econometric and statistical modeling with Python. In Proceedings of the Python in Science Conference, *57*, p. 10-25080. https://doi.org/10.25080/majora-92bf1922-011.

123. Efremova, M., Vento-Tormo, M., Teichmann, S.A., and Vento-Tormo, R. (2020). CellPhoneDB: inferring cell-cell communication from combined expression of multi-subunit ligand-receptor complexes. Nat. Protoc. *15*, 1484–1506. https://doi.org/10.1038/s41596-020-0292-x.

124. Türei, D., Valdeolivas, A., Gul, L., Palacio-Escat, N., Klein, M., Ivanova, O., Ölbei, M., Gábor, A., Theis, F., Módos, D., et al. (2021). Integrated intra- and intercellular signaling knowledge for multicellular omics analysis. Mol. Syst. Biol. *17*, e9923. https://doi.org/10.15252/msb.20209923.

125. Garcia-Alonso, L., Handfield, L.F., Roberts, K., Nikolakopoulou, K., Fernando, R.C., Gardner, L., Woodhams, B., Arutyunyan, A., Polanski, K., Hoo, R., et al. (2021). Mapping the temporal and spatial dynamics of the human endometrium in vivo and in vitro. Nat. Genet. *53*, 1698–1711. https://doi.org/10.1038/s41588-021-00972-2.

126. Sun, D., Guan, X., Moran, A.E., Wu, L.Y., Qian, D.Z., Schedin, P., Dai, M.S., Danilov, A.V., Alumkal, J.J., Adey, A.C., et al. (2022). Identifying phenotype-associated subpopulations by integrating bulk and single-cell sequencing data. Nat. Biotechnol. *40*, 527–538. https://doi.org/10.1038/s41587-021-01091-3.

127. Liu, J., Lichtenberg, T., Hoadley, K.A., Poisson, L.M., Lazar, A.J., Cherniack, A.D., Kovatich, A.J., Benz, C.C., Levine, D.A., Lee, A.V., et al. (2018). An integrated TCGA pan-cancer clinical data resource to drive high-quality survival outcome analytics. Cell *173*, 400–416.e11. https://doi.org/10.1016/j.cell.2018.02.052.

128. Sturm, G., Finotello, F., Petitprez, F., Zhang, J.D., Baumbach, J., Fridman, W.H., List, M., and Aneichyk, T. (2019). Comprehensive evaluation of transcriptome-based cell-type quantification methods for immuno-oncology. Bioinformatics *35*, i436–i445. https://doi.org/10.1093/bioinformatics/btz363.

129. Racle, J., de Jonge, K., Baumgaertner, P., Speiser, D.E., and Gfeller, D. (2017). Simultaneous enumeration of cancer and immune cell types from bulk tumor gene expression data. Elife *6*, e26476. https://doi.org/10.7554/eLife.26476.

130. Türei, D., Korcsmáros, T., and Saez-Rodriguez, J. (2016). OmniPath: guidelines and gateway for literature-curated signaling pathway resources. Nat Methods *13*, 966–967.

131. Philip, E., Alexander, P., Sven, F., Harshil, P., Johannes, A., Andreas, W., Maxime, U.G., Paolo, Di T., and Sven, N. (2022). The nf-core framework for community-curated bioinformatics pipelines (Zenodo).

132. Di Tommaso, P., Chatzou, M., Floden, E.W., Barja, P.P., Palumbo, E., and Notredame, C. (2017). Nextflow enables reproducible computational workflows. Nat Biotechnol *35*, 316–319. https://doi.org/10.1038/nbt.3820.

133. Kurtzer, G.M., Sochat, V., and Bauer, M.W. (2017). Singularity: Scientific containers for mobility of compute. PLoS One *12*, e0177459.

CellPress
OPEN ACCESS

STAR★METHODS

KEY RESOURCES TABLE

REAGENT or RESOURCE	SOURCE	IDENTIFIER
Antibodies		
Antibodies list		See Table S9
Biological samples		
Fresh resections of tumor tissue and adjacent normal lung tissue from NSCLC patients	This paper	N/A
Critical commercial assays		
BD TuDoR™ dissociation reagent	BD Biosciences	Cat#: 661563
BD Pharm Lyse™	BD Biosciences	Cat#: 555899
BD Rhapsody™ Cartridge Kit	BD Biosciences	Cat#: 633733
BD Rhapsody™ Cartridge Reagent Kit	BD Biosciences	Cat#: 633731
BD™ Human Single-Cell Multiplexing Kit	BD Biosciences	Cat#: 633781
BD Rhapsody™ WTA Amplification Kit	BD Biosciences	Cat#: 633801
BD Rhapsody™ cDNA Kit	BD Biosciences	Cat#: 633773
AMPure XP	Beckman Coulter	Cat#: A63880
Qubit™ dsDNA HS Assay Kit	Invitrogen	Cat#: Q32854
High Sensitivity D1000 Reagents	Agilent	Cat#: 5067–5585
High Sensitivity D5000 Reagents	Agilent	Cat#: 5067–5593
High Sensitivity D1000 ScreenTape	Agilent	Cat#: 5067–5584
High Sensitivity D5000 ScreenTape	Agilent	Cat#: 5067–5588
BD Pharmingen™ 7-AAD	BD Biosciences	Cat#: 559925
Calcein AM	Invitrogen	Cat#: C1430
Draq7	BD Biosciences	Cat#: 564904
Opal 7-Color Automated Immunohistochemistry Kit	Akoya Biosciences	Cat#: NEL821001KT
BOND Epitope Retrival 1	Leica Biosystems	Cat#: AR9961
BOND Epitope Retrival 2	Leica Biosystems	Cat#: AR9640
BOND Dewax Solution	Leica Biosystems	Cat#: AR9222
BOND Wash Solution 10x	Leica Biosystems	Cat#: AR9590
Spectral DAPI	Akoya Biosciences	Cat#: FP1490
Prolong Diamond Antifade	Thermo Fisher	Cat#: P36961
BOND Research Detection System	Leica Biosystems	Cat#: DS9455
BOND Titration Kit	Leica Biosystems	Cat#: OPT9049
BD TuDoR™ dissociation reagent	BD Biosciences	Cat#: 661563
Spectral DAPI	Akoya Biosciences	Cat#: FP1490
Prolong Diamond Antifade	Thermo Fisher	Cat#: P36961
BOND Research Detection System	Leica Biosystems	Cat#: DS9455
BOND Titration Kit	Leica Biosystems	Cat#: OPT9049
Deposited data		
BD Rhapsody dataset (demultiplexed UMI counts)	This study	https://doi.org/10.5281/zenodo.6411867
Processed input data	This study	https://doi.org/10.5281/zenodo.6411867
Results, including intermediate results, core and extended atlas in h5ad format, and scArches model	This study	https://doi.org/10.5281/zenodo.6411867
Core and extended atlas as cell-x-gene instance and h5ad/Seurat v3 files with standardized metadata according to the cell-x-gene schema	This study	https://cellxgene.cziscience.com/collections/edb893ee-4066-4128-9aec-5eb2b03f8287

(*Continued on next page*)

Continued		
REAGENT or RESOURCE	SOURCE	IDENTIFIER
Adams_Kaminski_2020 scRNA-seq dataset (processed)	GEO	GSE136831
Chen_Zhang2020 scRNA-seq dataset (fastq)	SRA	PRJNA634159
Goveia_Carmeliet_2020 (processed)	(Goveia *et al*., 2020)[6]	https://endotheliomics.shinyapps.io/lung_ectax/
Guo_Zhang_2018 (fastq)	EGA	EGAS00001002430
Habermann_Kropski_2020 (processed)	GEO	GSE135893
He_Fan_2021 (fastq)	NGDC GSA	CRA001963
Kim_Lee_2020 (processed)	GEO	GSE131907
Lambrechts_Thienpont_2018 (fastq)	ArrayExpress	E-MTAB-6149, E-MTAB-6653
Laughney_Massague_2020 (processed)	GEO	GSE123904
Madissoon_Meyer_2020 (processed)	(Madissoon et al., 2019)[92]	https://www.tissuestabilitycellatlas.org/
Maier_Merad_2020 (processed)	(Maier *et al*., 2020)[9]	https://github.com/effiken/Maier_et_al_nature_2020
Maynard_Bivona_2020 (fastq)	SRA	PRJNA591860
Mayr_Schiller_2020 (processed)	(Mayr et al., 2021)[90]	https://github.com/theislab/2020_Mayr
Reyfman_Misharin_2018 (processed)	GEO	GSE122960
Travaglini_Krasnow_2020 (processed)	(Travaglini et al., 2020)[97]	https://www.synapse.org/#!Synapse:syn21560406
Vieira_Teichmann_2019 (processed)	GEO	GSE130148
Wu_Zhou_2021 (processed)	GEO	GSE148071
Zillionis_Klein_2019 (processed)	GEO	GSE127465
Leader_Merad_2021 (processed)	GEO	GSE154826
TCGA data RNA-seq and mutation data	GDC	https://portal.gdc.cancer.gov/
TCGA survival data	(Liu et al., 2018)[127]	
Cytosig signatures	(Jiang *et al*., 2021)[36]	https://github.com/data2intelligence/CytoSig/
CellPhoneDB	(Efremova et al., 2020[123]; Türei et al., 2016)[130]	Downloaded from https://omnipathdb.org/interactions/?fields=sources,references&genesymbols=1&databases=CellPhoneDB on 2022-04-06
Software and algorithms		
Seven Bridges - BD Rhapsody™ WTA Analysis Pipeline	Seven Bridges Genomics	v1.7.1
FlowJo™	BD Biosciences	v10.7
Mantra Snap	Akoya Biosciences	v1.0.4
inForm Tissue Analysis	Akoya Biosciences	v2.4.10
GraphPad Prism	Graphpad	v9
Cellranger v5.0.0	10x Genomics	https://support.10xgenomics.com/single-cell-gene-expression/software/pipelines/latest/what-is-cell-ranger
nf-core RNA-seq pipeline v3.0	(Philip et al., 2022)[131]	https://github.com/nf-core/rnaseq
Nextflow v22.04.5	(Di Tommaso et al., 2017)[132]	https://nextflow.io
Singularity/Apptainer v3.7.0–1.el7	(Kurtzer et al., 2017)[133]	https://apptainer.org/
Nextflow workflow to reproduce this study	This study	https://github.com/icbi-lab/luca (https://doi.org/10.5281/zenodo.7104045)
Software packages used for scRNA-seq analysis are packaged as singularity containers and available on zenodo	This study	https://doi.org/10.5281/zenodo.6411867
Seven Bridges - BD Rhapsody™ WTA Analysis Pipeline	Seven Bridges Genomics	v1.7.1
FlowJo™	BD Biosciences	v10.7
Mantra Snap	Akoya Biosciences	v1.0.4
inForm Tissue Analysis	Akoya Biosciences	v2.4.10

(*Continued on next page*)

Continued

REAGENT or RESOURCE	SOURCE	IDENTIFIER
GraphPad Prism	Graphpad	v9
Cellranger v5.0.0	10x Genomics	https://support.10xgenomics.com/single-cell-gene-expression/software/pipelines/latest/what-is-cell-ranger
Other		
Compute node CPU	Intel	Xeon(R) CPU E5-2699A v4 (2x)
GPU node GPU	Nvidia	Quadro RTX 8000
GPU node CPU	AMD	EPYC 7352 24-Core (2x)
Atlas web resource	This study	https://luca.icbi.at

RESOURCE AVAILABILITY

Lead contact

Further information and requests for resources and reagents should be directed to and will be fulfilled by the lead contact, Zlatko Trajanoski (zlatko.trajanoski@i-med.ac.at)

Materials availability

The study did not generate new unique reagents.

Data and code availability

- Processed scRNA-seq data from this study has been deposited on Zenodo as listed in the key resources table. Raw data is not made available due to privacy concerns.
- Processed scRNA-seq data from other studies has been deposited on Zenodo. The original study identifiers are listed in the key resources table.
- Final and intermediate results of the computational analysis are made available on Zenodo.
- All code to reproduce this study is wrapped into a nextflow workflow and publicly available on Github. All software dependencies are made available as singularity containers. Some of the algorithms employed (scVI, scANVI, UMAP) involve stochastic processes that require specific hardware for exact reproducibility (see key resources table).
- Microscopy data reported in this paper will be shared by the lead contact upon request. Any additional information required to reanalyze the data reported in this paper is available from the lead contact upon request.

EXPERIMENTAL MODEL AND SUBJECT DETAILS

Human subjects

Samples of NSCLC tumor tissues and matched adjacent normal lung tissues (more than 5 cm distance to the tumor) were obtained from surgical specimens of patients undergoing resection at the Department of Visceral, Transplant and Thoracic Surgery (VTT), Medical University Innsbruck, Austria, and in collaboration with the INNPATH GmbH, Innsbruck, Austria, after obtaining informed consent in accordance with a protocol reviewed and approved by the Institutional Review Board at the Medical University Innsbruck, Austria (study code: AN214-0293 342/4.5). Demographic details are provided in Table S6.

METHOD DETAILS

Preparation of NSCLC tissue and normal lung tissue

Surgically resected NSCLC tumor tissues and adjacent normal tissues were minced into small pieces (<1 mm) on ice and enzymatically digested with agitation for 30 min at 37°C using the BD TuDoR™ dissociation reagent (BD Biosciences). The obtained single-cell solution was sieved through a 70 μM cell strainer (Corning) and red blood cells were removed using the BD Pharm Lyse™ lysing solution (BD Biosciences). Cells were counted and viability assessed with the BD Rhapsody scRNA-seq platform (BD Biosciences) using Calcein-AM (Invitrogen) and Draq7 (BD Biosciences).

BD Rhapsody library preparation and sequencing

Freshly isolated single-cells were immediately processed with the BD Rhapsody scRNA-seq platform (BD Biosciences). The BD Single-Cell Multiplexing Kit (BD Biosciences) was used to combine and load two samples (tumor tissue and normal adjacent tissue) onto a single BD Rhapsody™ cartridge (BD Biosciences). Sample-tag staining was performed according to the manufacturer's

protocol (sample-tag staining at room temperature for 20 min and washing by centrifugation at 400 g for 5 min). Single-cell isolation in microwells (cell load: 20 min incubation at room temperature) with subsequent cell-lysis and capturing of poly-adenylated mRNA molecules with barcoded, magnetic capture-beads was performed according to the manufacturer's instructions. Beads were magnetically retrieved from the microwells, pooled into a single tube before reverse transcription. Unique molecular identifiers (UMIs) were added to the cDNA molecules during cDNA synthesis. Whole transcriptome amplification (WTA) and sample-tag sequencing libraries were generated according to the BD Rhapsody single-cell whole-transcriptome amplification workflow. The quantity and quality of the sequencing libraries was analyzed with the Qubit dsDNA HS (High Sensitivity) assay kit (Invitrogen) and the 4200 TapeStation (Agilent) system. Libraries were sequenced on the Novaseq 6000 system (Illumina) targeting a sequencing depth of 50.000 reads/cell.

Flow cytometry

Cells isolated from surgically resected NSCLC tumor tissues and adjacent normal tissues were stained with a backbone cocktail of 12 antibodies (CD56, CD3, CD8, CD4, CD45, HLA-DR, CD31, CD14, CD15, CD326, CD19, CD16) which, was complemented either with an additional 8 antibodies (CD28, CD38, CD123, CD34, CD161, CD193, TCRgd, CD90) to define all cell populations, or several mixtures of up to three antibodies (CD54, CD83, CD49b, CD62L, LOX-1, CD181) for a detailed characterization of neutrophils, at pre-titrated concentrations. All source data of the aforementioned antibodies is provided in the key resources table, applied flourochromes are listed in Table S7. After washing and addition of 5 μL 7-AAD, the cells were measured on a FACSymphony A5 flow cytometer (BD Biosciences). Data were analyzed using FlowJo v10.7 software. For details of the gating strategy see Figure S7.

Multiplex immunofluorescence

NSCLC tumor and tumor-adjacent tissue samples were fixed in 4% formalin for 6–72 h and embedded in paraffin. Four-micrometer sections were used for the immunofluorescence staining. Immunofluorescence staining on formalin-fixed paraffin-embedded (FFPE) tissue was performed using the Opal 7-Color Automated Immunohistochemistry Kit (cat: NEL821001KT, Akoya Biosciences, Menlo Park, USA). A multiplex panel of immune markers was developed with antibodies against: CD16 (clone EPR22409-124, Abcam), CD8 (clone C8/144B, Dako), CD68 (clone PG-M1, Dako), CD3 (polyclonal, Dako), CD20 (clone L26, Dako), cytokeratin (clone AE1/AE3, Dako; clone C-11, Abcam). In additional settings antibodies were used against: CD16 (clone EPR22409-124, Abcam), CXCR2 (clone EPR22301-103, Abcam), OLR1 (polyclonal, Sigma Life Science) (further information given in Table S7). The staining procedure was performed using an automated staining system (BOND-RX, Leica Biosystems). All markers were sequentially applied and paired with respective Opal fluorophores (Table S7). To visualize cell nuclei, the tissue was stained with 4‘,6-diamidino-2-phenylindole (spectral DAPI, Akoya Biosciences). Stained slides were scanned using Mantra 2 Quantitative Pathology Workstation (Akoya Biosciences) and representative images from each tissue were acquired with the Mantra Snap software v1.0.4. Spectral unmixing, multispectral image analysis and cell phenotyping was carried out using the inForm Tissue Analysis Software v2.4.10 (Akoya Biosciences). In short, DAPI staining was used to segment cells. The perinuclear area (the defined 4-pixel area around nuclei) was therefore defined to be cell cytoplasm. Thereafter, the total cell area was evaluated for nucleic/cytoplasmic/membrane marker expression. The inForm build-in algorithm for cell phenotyping was used to define the intensity threshold for the positivity of each marker individually and each cell was characterized/phenotyped by presence/absence of the marker.

Immunohistochemistry

Staining of the validation cohort was approved by the Internal Review Board of the University of Luebeck (file number 16–277). Tissue microarrays (TMA) were constructed from formalin-fixed paraffin-embedded (FFPE) tumor blocks originating from surgical samples. In short, for TMA construction each sample was represented in triplicates of 0.6 mm diameter cores. A tumor sample was incorporated in further analysis if at least one core was evaluable. The validation cohort included 55 chemo-naive LUAD and LUSC, respectively, with no history of previous malignancies or history of receiving chemotherapy or radiotherapy. Immunohistochemistry (IHC) staining was performed according to the manufacturer's instructions, using the Ventana Discovery (Ventana Medical System) automated staining system. Slides were incubated with a primary antibody against CD4 (CD4 SP35, Ventana, RTU) to detect $CD4^+$ lymphocytes and with a primary antibody against CD68 (KP1, Ventana, RTU) to detect macrophages (Table S7). They were further stained with chloroacetate-esterase (Naphthol-AS-D-chloracetate, Serva) to highlight neutrophils. To evaluate the immune cell infiltration, $CD4^+$ lymphocytes, macrophages and neutrophils were counted in three high power fields (HPFs) per core, meaning that up to nine HPFs per case were assessed. Two experienced pathologists (SP and CK) performed an independent evaluation of the slides.

Staining of CXCR2 to detect neutrophils was performed using the rabbit antibody against CXCR2 (EPR22301-103, Abcam) diluted in a Primary Antibody Diluent Buffer (Primary antibody diluent, Leica Biosystems). The staining protocol included a standard antigen retrieval step with CC1/pH9 buffer (Discovery CC1, Ventana), incubation with the primary antibody for 0.5 h at room temperature (RT). Antibody staining was detected by DAB. Neutrophils were counted in 5 HPFs.

Generation of the core atlas

The data for the core atlas was from previously published NSCLC studies,[3–8,10,12,15,90,91] our own data (n = 3), and 7 studies for control purpose.[91–97] The selected studies were published between July 2018 and May 2021 and the incorporated datasets were generated with six different sequencing platforms, including the most commonly applied 10x Chromium (10x Genomics) as well as

Smart-seq2,[98] GEXSCOPE (Singleron), inDrop[99] and Drop-Seq.[100] We further integrated our own data generated with the microwell-based BD Rhapsody scRNA-seq platform (BD Biosciences). We specifically selected studies using comparable protocols for sample processing and data generation, such as sequencing of whole cells. We did not exclude studies that applied flow cytometry-based cell-sorting prior to sequencing, as these incorporate relevant information on rare cell types.[6,9,15] From non-NSCLC studies we exclusively included those parts of the published data that were relevant for our atlas: from the Madissoon dataset[92] we only included lung samples, from Adams,[95] Reyfman,[93] Habermann,[96] Vieira Braga,[94] and Mayr[90] datasets we only used the control samples (including normal lung tissue of tumor patients, which we termed "normal_adjacent" or lung tissue of organ donors without history of pulmonary disease). From the Adams et al. dataset we also included data from patients (n = 18) with chronic obstructive pulmonary disease (COPD) as chronic inflammatory pulmonary disease cohort with an increased lung cancer risk.

Preprocessing and quality control of scRNA-seq data

We distinguish between studies (i.e. a scientific publication) and datasets (i.e. scRNA-seq samples that were generated using the same sample preparation and the same experimental platform). Each study may contain one or multiple datasets. Demultiplexed FASTQ files of the UKIM-V datasets were merged and processed using the Seven Bridges Genomics cloud server with the BD Rhapsody WTA Analysis Pipeline. Samples from the studies Chen_Zhang_2020, Guo_Zhang_2018, He_Fan_2021, Lambrechts_Thienpont_2018 and Maynard_Bivona_2020 were obtained as raw fastq files from the identifiers specified in the key resources table. Smart-seq2 data were processed using the nf-core RNA-seq pipeline[101,102] with the GRCh38 reference genome and GENCODE v33 annotations. 10x datasets were processed with cellranger v5.0.0 (10x Genomics) and the GRCh38-2020-A reference database as provided by 10x Genomics. All other datasets were obtained as count tables from their respective identifiers. All datasets were loaded into AnnData containers[103] with consistent structure. Quality control was performed with scanpy[104] by thresholding the number of detected genes, counts and the fraction of mitochondrial reads. Thresholds were determined per dataset by visual inspection of the distributions and are listed in Table S8.

Integration of scRNA-seq datasets

Individual datasets were merged into a single AnnData object. Since genome annotations partly differed between the datasets, we re-mapped gene identifiers on the latest version of HGNC gene symbols using the https://mygene.info API.[105] In case of duplicate gene symbols, the one with the maximum read count was retained. If gene symbols were missing from a dataset, the values were filled with zeros. Gene symbols that were missing in more than 5 datasets (25%) were excluded altogether.

We integrated the datasets using the scANVI algorithm,[106,107] as it has been demonstrated to be one of the top-performing methods for atlas-level integration and to scale to >1M cells.[108] Since scANVI requires cell-type annotations for at least one of the input datasets, we manually annotated two "seed" datasets based on unsupervised clustering as described below. We chose Lambrechts_Thienpont_2018_6653 and Maynard_Bivona_2020 as seed datasets as they were not experimentally enriched for specific cell-types and were sequenced on two platforms with very different characteristics (10x and Smart-seq2). Raw counts were used as input for scANVI. The Smart-seq2 counts were scaled by the gene length as recommended on the scvi-tools website. The scANVI model was initialized with a pre-trained scVI model,[109] as recommended in the scvi-tools tutorial. The scVI model was trained on the 6000 most highly variable genes as determined with scanpy's[104] *pp.highly_variable_genes* with parameters *flavor="seurat_v3"* and *batch_key="dataset"*. Each sample was considered as an individual batch for both scVI and scANVI. Other than that the algorithms were run with default parameters.

Doublet-detection

For droplet-based scRNA-seq datasets we ran the SOLO algorithm[110] to computationally detect multiplets. We chose SOLO over other doublet detection methods as it is readily integrated into scvi-tools,[107] and was found to be one of the top-performing methods in an independent benchmark.[111] We used the SOLO implementation from scvi-tools and initialized SOLO with a pre-trained scVI model.

Unsupervised clustering and cell-type annotation

We computed UMAP embeddings[112] and unsupervised Leiden-clustering[113] with scanpy,[104] based on a cell-cell neighborhood graph derived from scANVI latent space. Coarse, lineage-specific clusters were iteratively sub-clustered to identify cell-types at a more fine-grained resolution. Cell type clusters were annotated based on previously reported marker genes[92,114,115] (Figure S1A). $CD8^+$ T cell subclusters were annotated based on gene sets from Oliveira et al.[22]

Integrating additional datasets

Two datasets, Leader_Merad_2021 and UKIM-V-2, were added after the completion and annotation of the core atlas. The datasets ("query") were projected onto the atlas ("reference") using scArches[29] as implemented in scvi-tools.[107] scVI and scANVI models were re-trained on the fully annotated, doublet-filtered core atlas, with the parameters recommended for scArches: *use_layer_norm="both"*, *use_batch_norm="none"*, *encode_covariates=True*, *dropout_rate=0.2*, *and n_layers=2*. Gene-symbols of the query datasets were re-mapped as described above and missing gene symbols filled with zeros. For each query dataset, scArches yielded an embedding in the same latent space as the core atlas. Based on the joint latent space, a neighborhood graph and UMAP embedding were computed for the "extended" atlas. Cell-types were annotated automatically, based on a majority vote of nearest

neighbors. To this end, let C be the pairwise weighted connectivity matrix of the scanpy neighborhood graph computed on the scArches embedding. Then, the transitive connectivity matrix C' (i.e., including connections to neighbors of neighbors) is defined as $C' = C \cdot C$ where the dot operator refers to the matrix product. Let further Q be the set of all query cells, R the set of all reference cells, and T the set of all cell-types. Then, for every cell $q \in Q$ the cell-type is determined as

$$\underset{t \in T}{argmax} \sum_{r \in R} \sigma(t, r)\, C'_{qr}$$

where the indicator function $\sigma(t, r)$ is 1 if cell r is of type t and 0 otherwise. The transitive connectivity matrix C' was chosen over C to increase robustness by increasing the number of neighbors, and to ensure that every cell from the query has connection to a cell in the reference.

Comparing cell-type abundances

Since comparing cell-type fractions between groups is challenging due to different characteristics of the datasets and the inherent compositional nature of cell-type fractions, we applied the scCODA[30] model, which addresses this issue. We were interested in the differences between conditions (LUAD *vs*. LUSC). To this end, we ran the scCODA model with the formula $\sim$ *condition* + *tumor_stage* + *dataset* with 500,000 iterations using "cancer cells" as the reference cell-type, where *tumor_stage* is a binary vector classifying datasets into early (stages I-II) and advanced (stages III-IV), and dataset is a categorical vector encoding the different datasets. For the comparison, we excluded the Guo_Zhang_2018 dataset, which only contains T cells. The final result shows *credible effects* with a false-discovery-rate (FDR) of 0.1.

Patient stratification

We stratified patients into immune phenotypes based on immune cell-type fractions. We selected all patients with primary tumor samples and excluded the Guo_Zhang_2018 dataset, because it contains only T cells. Neutrophil fractions were excluded, since they are not appropriately captured in the majority of datasets. Cell-type fractions of primary tumor samples were loaded into a patient × cell-type AnnData container. Dataset-specific batch-effects were removed using a linear model as implemented in *scanpy.pp.regress_out*. Patients were clustered using graph-based Leiden clustering with the "correlation" distance metric for computing the neighborhood graph. Patient clusters were labeled according to their predominant cell-types. In addition to the histological subtypes based on the annotation of the original datasets, we annotated tumor types based on the transcriptomics data according to the most abundant cancer cell cluster.

RNA velocity analysis

We performed RNA-velocity analysis on the UKIM-V dataset using velocyto.py[116] and scvelo.[117] BAM files as generated by the BD Rhapsody WTA analysis pipeline were preprocessed with samtools[118] to make them compatible with velocyto.py (see preprocessing/bd_rhapsody/velocyto.nf in our git repository for more details). Loom files generated by velocyto.py were loaded into scvelo to estimate and visualize RNA velocities according to the scvelo tutorial. Partition-based graph abstraction (PAGA,[73]) was computed based on the RNA velocity graph, using neutrophil subclusters as grouping variable and the option *minium_spanning_tree=False*. The result was visualized as a graph showing the transition confidences as directed edges.

Differential gene expression testing

We used DESeq2[119] on pseudo-bulk samples for differential expression testing which has been demonstrated to perform well and properly correct for false discoveries.[120] For each cell-type and patient, we summed up transcript counts for each gene. Pseudo-bulk samples consisting of fewer than 10 cells were discarded. We compared primary tumor samples from LUAD *vs*. LUSC (*condition*), primary tumor samples from the patient subtypes M/B/T/desert (*group*), NANs *vs*. TANs (*cell_type_tan_nan*), and Neutrophil clusters (NAN1-3, TAN1-4), including the dataset as a covariate. For comparisons between multiple groups, we used contrasts with sum-to-zero coding. p-values were adjusted for multiple hypothesis testing with independent hypothesis weighting (IHW).[121]

Pathway, TF and cytokine signaling signatures

We performed pathway, transcription factor (TF), and cytokine signaling analysis on primary tumor samples with PROGENy,[31,34] DoROthEA[33,34] and CytoSig,[36] respectively. Scores were computed using the *dorothea-py* and *progeny-py* packages. The top 1,000 target genes of the progeny model were used, as recommended for single-cell data. For dorothea, only regulons of the highest confidence levels "A" and "B" were used. The cytosig signature matrix was obtained from the *data2intelligence/CytoSig* GitHub repository and used with the scoring function implemented in the *progeny-py* package. The methods were run with the options *num_perm=0*, *center=True*, *norm=True scale=True*, and *min_size=5*. No permutations were used, as we perform statistics in a separate step at the level of biological replicates. Pathway-, transcription factor-, and cytosig scores were then compared between *condition* (LUAD *vs*. LUSC) and patient *group* (T *vs*. B *vs*. M *vs*. deserted) using an ordinary least-squares (OLS) linear model, as implemented in the statsmodels package.[122] Scores were aggregated into pseudobulk samples by computing the mean of each variable for each patient and cell-type. Samples consisting of less than 10 cells were discarded. For each variable, we fitted a model with the formulas $\sim$ *condition* + *dataset* + *tumor_stage* or $\sim$ *group* + *dataset* + *condition* + *tumor_stage*, respectively. Coefficients were obtained from the linear model and p-values calculated with the f-test. p-values were adjusted for multiple testing with the Benjamini-Hochberg procedure.

Cellphonedb analysis

We used the cellphonedb (CPDB) database[123] as obtained from omnipathdb[124] to investigate differences in cell-to-cell communication in primary tumor samples. The original CPDB algorithm performs statistical comparisons based on a permutation test which is designed to find differences between cell-types. For our study, on the other hand, we were interested in differences between conditions, using patients as biological replicates. Therefore, we followed an approach similar to the *degs_analysis* mode recently added to CPDB v3[125]: For each cell-type of interest, we considered the list of significantly differentially expressed signaling molecules in CPDB (ligands or receptors, for outgoing and incoming interactions, respectively). For each of those differentially expressed signaling molecules and for each cell-type, we determined interaction partners that are potentially affected by that change, as those that are expressed in at least 10% of the cells in a certain cell-type. Differentially expressed signaling molecules were determined with DESeq2 as described above. The fraction of cells expressing a signaling molecule was computed as the mean of fractions per patient, to avoid biases due to different cell-counts per patient.

SCISSOR analysis

We used SCISSOR[126] to associate phenotypic data from bulk RNA-seq experiments with our single-cell data. TCGA mutation and gene expression data was obtained from the GDC portal, survival data from.[127] SCISSOR was run on primary tumor cells of each patient individually according to the SCISSOR tutorial using mutation data (logistic regression) and overall survival (cox-regression) as dependent variables. A grid search for the alpha-parameter was performed in $2^{-i/2}$ with $i \in [24, 23, \ldots, 2]$ and a cutoff parameter of 0.3. 21 of 176 samples with low overall cell count failed during SCISSOR's Seurat-preprocessing step and were excluded from the subsequent analysis. For each patient and cell-type, we computed the fraction of *scissor + cells* (i.e. positively associated with a mutation or worse survival), *scissor- cells* (i.e. negatively associated), and *neutral cells* and added a pseudo-count of 0.01. A sample was excluded from a cell-type if it contributed $\leq$ 10 cells. For each cell-type, we computed the log2-ratio of scissor+ and scissor- cells as the mean fraction of scissor + cells *vs*. the mean fraction of scissor- cells. Significant differences were determined by comparing the fractions of scissor+ and scissor- cells with a paired wilcoxon test with *zero_method="zsplit"* as implemented in the scipy package. p-values were Benjamini-Hochberg-adjusted and considered significant at an FDR <0.01.

TRN clusters

For an unbiased discovery of TRN subtypes, we performed unsupervised clustering of all cells annotated as neutrophils. The neighborhood graph was computed with *scanpy.pp.neighbors* with *n_neighbors=30* based on the scANVI latent space. Clusters were determined with *scanpy.tl.leiden* with *resolution=0.75*. Two subclusters dominated by cells from normal adjacent tissue were labeled normal-associated neutrophils (NAN) 1, 2, and 3, whereas four subclusters of cells from primary tumor samples were labeled tumor-associated neutrophils (TAN) 1, 2, 3 and 4.

TRN signatures

Gene signatures for TRN and TRN clusters were determined based on fold-change (FC), specific fold-change (sFC), and area under the receiver operator characteristics curve (AUROC), applying an approach previously used to find cell-type-specific marker genes.[78] We have previously shown the resulting gene signatures to be highly specific for their respective cell-types.[128] To avoid marker genes being biased towards samples contributing more cells than on average we aggregated single cells to pseudo-bulk samples[120] by patient before deriving marker genes. For each set of marker genes derived, pseudo-bulk samples were generated by summing up raw counts for each patient and cell-type of interest. The resulting samples were normalized to counts per million (CPM) and log2-transformed with *scanpy.pp.log1p(adata, base=2)*. Pseudo-bulk samples consisting of fewer than 10 cells were discarded. For each gene and cell-type, FC and sFC were computed as described in.[78] AUROC was computed using *roc_auc_score* as implemented in scikit-learn. For identifying marker genes for the 7 neutrophil subclusters, we applied a permissive cutoff of sFC >1 and FC > 1.5 and ranked genes by AUROC. For the TAN and NAN signature used to compute signature scores in bulk RNA-seq data, we empirically determined optimal cut-offs by grid search and cross-validation: First, the single-cell input data were randomly split by patients into 80% training data and 20% independent test set. On the training data, five-fold cross validation was performed. On the training set of each fold, metrics were computed as described above and all possible combinations of sFC $\in$ {0.5, 0.6, …, 2.9}, FC $\in$ {0.5, 0.6, …, 2.9} and AUROC $\in$ {0.7, 0.75, 0.8, 0.85, 0.9, 0.95, 0.96, 0.97} were tested, resulting in a total of 5,000 possible signatures. On the test set of each fold, a pseudo-bulk sample per patient (mixing all cell-types) was generated and the true fraction of the cell-type of interest calculated. The quality of each signature was evaluated as the Pearson correlation between the signature score (see section "Signature scoring in bulk RNA-seq samples" below) and the true cell-type fraction. The cut-off with the highest average correlation across the five folds was chosen as optimal. Finally, the signature was re-calculated on the entire training set using the optimal cut-off, and a final Pearson correlation determined on the independent test set. We defined a TRN signature to capture Neutrophils independent of their subtype as the union of the TAN and the NAN signature genes.

Signature scoring in bulk RNA-seq samples

Signature scores in scRNA-seq data were computed using *scanpy.tl.score_genes*. Bulk RNA-seq primary tumor samples samples of TCGA LUAD and LUSC were retrieved as TPM from the GDC portal. Bulk RNA-seq samples from NSCLC patients treated with atezolizumab (anti-PD-L1) or docetaxel (chemotherapy) from the POPLAR[79] and OAK[80] trials were retrieved using the accession numbers reported in.[81] Similar to an approach previously described,[11] enrichment scores for our TRN signatures were calculated

as follows: For all signature genes, z-scores were computed across all samples from a dataset. The final signature score was defined as the mean of the z-scores of the signature genes for each sample. Associations of the TRN signature with response to immunotherapy or chemotherapy in the POPLAR and OAK datasets was tested using logistic regression in R with the formula *response* ~ *signature_score* + *tumor_type* + *dataset*, where tumor_type represents LUAD and LUSC encoded as a binary vector.

Survival analysis

Survival analysis was performed using CoxPH-regression as implemented in the R package *survival*. Kaplan-Meyer plots were created using the R package *survminer*, showing the top 25% *vs*. bottom 25% of samples stratified by signature score. B cell fractions in TCGA samples were estimated using EPIC[129] as implemented in *immunedeconv*, as we have previously shown EPIC to be one of the best performing methods on B cells.[128] Cox-regression was performed on B cell fractions (TCGA data) with the formula *survival* ~ *signature_score* + *ajcc_stage* + *age*, where ajcc_stage is a categorical vector with tumor stages I-IV. For neutrophil fractions (POPLAR + OAK data) the formula *survival* ~ *signature_score* + *dataset* + *treatment* was used. For comparisons comprising the entire NSCLC cohort (i.e. both LUAD and LUSC), *tumor_type* was included as an additional covariate.

QUANTIFICATION AND STATISTICAL ANALYSIS

Statistical analysis was performed using the statsmodels library in Python (scRNA-seq data) or GraphPad Prism (flow cytometry and imaging data) using a linear model, t-test or wilcoxon test as appropriate. Single cell-data were aggregated into pseudobulk samples by biological replicates. Compositional analysis of cell-type fractions was performed using scCODA; survival analysis using CoxPH regression in R. P-values for untargeted analyses (DE genes, TFs, or pathways) were FDR-adjusted. Significance levels and more details on the statistical tests are indicated in the figure captions.

ADDITIONAL RESOURCES

The single-cell atlas can be assessed *via* cell-x-gene (https://luca.icbi.at), a web-based viewer for single-cell datasets that allows visualization of metadata and gene expression.

Cancer Cell, Volume *40*

Supplemental information

High-resolution single-cell atlas reveals diversity and plasticity of tissue-resident neutrophils in non-small cell lung cancer

Stefan Salcher, Gregor Sturm, Lena Horvath, Gerold Untergasser, Christiane Kuempers, Georgios Fotakis, Elisa Panizzolo, Agnieszka Martowicz, Manuel Trebo, Georg Pall, Gabriele Gamerith, Martina Sykora, Florian Augustin, Katja Schmitz, Francesca Finotello, Dietmar Rieder, Sven Perner, Sieghart Sopper, Dominik Wolf, Andreas Pircher, and Zlatko Trajanoski

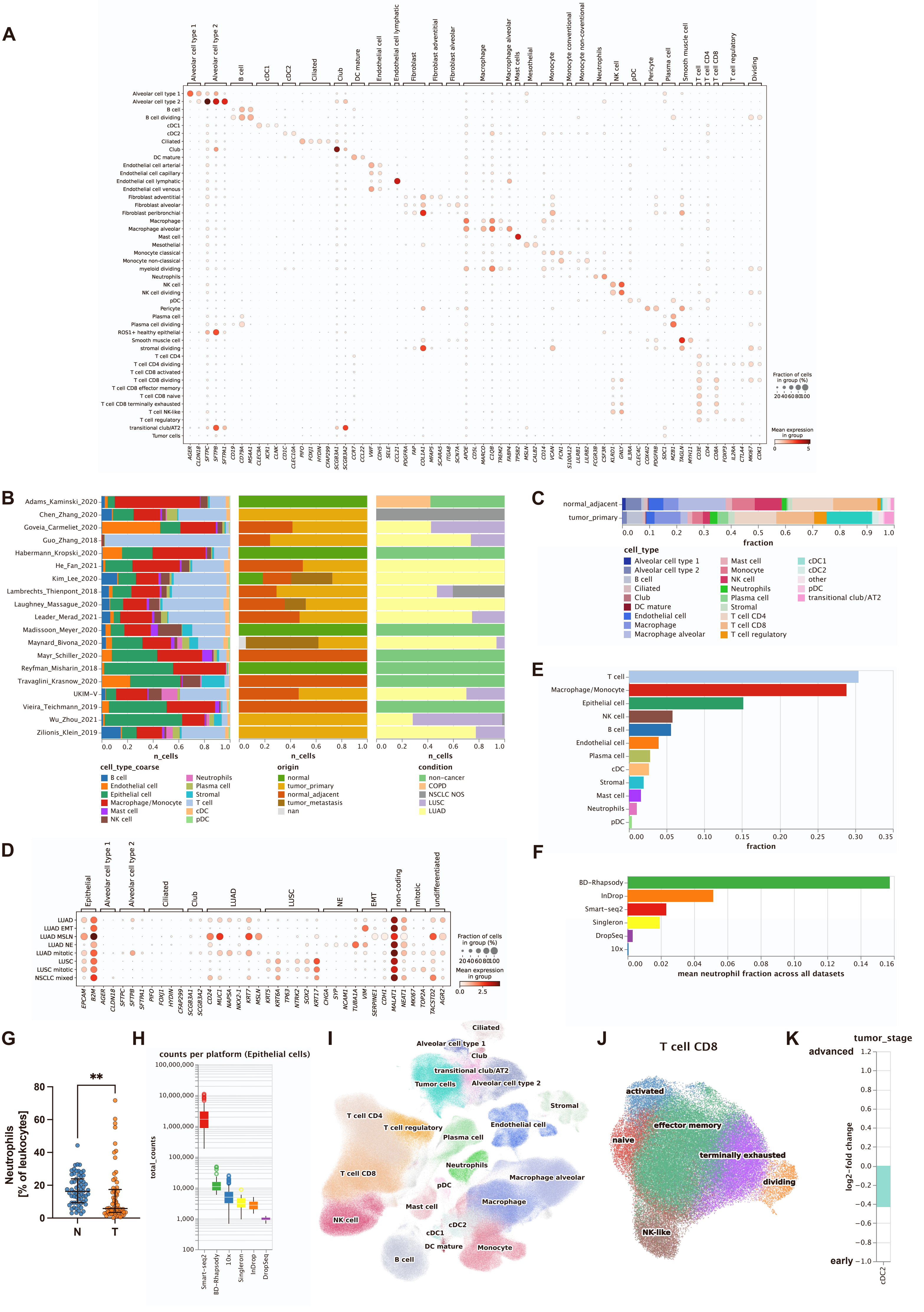
A
Alveolar cell type 1
Alveolar cell type 2
B cell
cDC1
cDC2
Ciliated
Club
DC mature
Endothelial cell
Endothelial cell lymphatic
Fibroblast
Fibroblast adventitial
Fibroblast alveolar
Macrophage
Macrophage alveolar
Mast cells
Mesothelial
Monocyte
Monocyte conventional
Monocyte non-coventional
Neutrophils
NK cell
pDC
Pericyte
Plasma cell
Smooth muscle cell
T cell
T cell CD4
T cell CD8
T cell regulatory
Dividing
Alveolar cell type 1
Alveolar cell type 2
B cell
B cell dividing
cDC1
cDC2
Ciliated
Club
DC mature
Endothelial cell arterial
Endothelial cell capillary
Endothelial cell lymphatic
Endothelial cell venous
Fibroblast adventitial
Fibroblast alveolar
Fibroblast peribronchial
Macrophage
Macrophage alveolar
Mast cell
Mesothelial
Monocyte classical
Monocyte non-classical
myeloid dividing
Neutrophils
NK cell
NK cell dividing
pDC
Pericyte
Plasma cell
Plasma cell dividing
ROS1+ healthy epithelial
Smooth muscle cell
stromal dividing
T cell CD4
T cell CD4 dividing
T cell CD8 activated
T cell CD8 dividing
T cell CD8 effector memory
T cell CD8 naive
T cell CD8 terminally exhausted
T cell NK-like
T cell regulatory
transitional club/AT2
Tumor cells
AGER CLDN18 SFTPC SFTPB SFTPA1 CD19 CD79A MS4A1 CLEC9A XCR1 CLNK CD1C CLEC10A PIFO FOXJ1 HYDIN CFAP299 SCGB3A1 SCGB3A2 CCR7 CCL22 VWF CDH5 SELE CCL21 PDGFRA FAP COL1A1 MFAP5 SCARA5 ITGA8 SCN7A APOE CD5L MARCO C1QB TREM2 FABP4 TPSB2 MSLN CALB2 CD14 VCAN FCN1 S100A12 LILRB1 LILRB2 FCGR3B CSF3R KLRD1 GNLY IL3RA CLEC4C COX4I2 PDGFRB SDC1 MZB1 TAGLN MYH11 CD3E CD4 CD8A FOXP3 IL2RA CTLA4 MKI67 CDK1
Fraction of cells in group (%)
20 40 60 80 100
Mean expression in group
0 5
B
Adams_Kaminski_2020
Chen_Zhang_2020
Goveia_Carmeliet_2020
Guo_Zhang_2018
Habermann_Kropski_2020
He_Fan_2021
Kim_Lee_2020
Lambrechts_Thienpont_2018
Laughney_Massague_2020
Leader_Merad_2021
Madissoon_Meyer_2020
Maynard_Bivona_2020
Mayr_Schiller_2020
Reyfman_Misharin_2018
Travaglini_Krasnow_2020
UKIM-V
Vieira_Teichmann_2019
Wu_Zhou_2021
Zilionis_Klein_2019
0.0 0.2 0.4 0.6 0.8 1.0
n_cells
cell_type_coarse
B cell
Endothelial cell
Epithelial cell
Macrophage/Monocyte
Mast cell
NK cell
Neutrophils
Plasma cell
Stromal
T cell
cDC
pDC
origin
normal
tumor_primary
normal_adjacent
tumor_metastasis
nan
condition
non-cancer
COPD
NSCLC NOS
LUSC
LUAD
C
normal_adjacent
tumor_primary
0.0 0.1 0.2 0.3 0.4 0.5 0.6 0.7 0.8 0.9 1.0
fraction
cell_type
Alveolar cell type 1
Alveolar cell type 2
B cell
Ciliated
Club
DC mature
Endothelial cell
Macrophage
Macrophage alveolar
Mast cell
Monocyte
NK cell
Neutrophils
Plasma cell
Stromal
T cell CD4
T cell CD8
T cell regulatory
cDC1
cDC2
other
pDC
transitional club/AT2
E
T cell
Macrophage/Monocyte
Epithelial cell
NK cell
B cell
Endothelial cell
Plasma cell
cDC
Stromal
Mast cell
Neutrophils
pDC
0.00 0.05 0.10 0.15 0.20 0.25 0.30 0.35
fraction
D
Epithelial
Alveolar cell type 1
Alveolar cell type 2
Ciliated
Club
LUAD
LUSC
NE
EMT
non-coding
mitotic
undifferentiated
LUAD
LUAD EMT
LUAD MSLN
LUAD NE
LUAD mitotic
LUSC
LUSC mitotic
NSCLC mixed
EPCAM B2M AGER CLDN18 SFTPC SFTPB SFTPA1 PIFO FOXJ1 HYDIN CFAP299 SCGB3A1 SCGB3A2 CD24 MUC1 NAPSA NKX2-1 KRT7 MSLN KRT5 KRT6A TP63 NTRK2 SOX2 KRT17 CHGA SYP NCAM1 TUBA1A VIM SERPINE1 CDH1 MALAT1 NEAT1 MKI67 TOP2A TACSTD2 AGR2
Fraction of cells in group (%)
20 40 60 80 100
Mean expression in group
0.0 2.5
F
BD-Rhapsody
InDrop
Smart-seq2
Singleron
DropSeq
10x
0.00 0.02 0.04 0.06 0.08 0.10 0.12 0.14 0.16
mean neutrophil fraction across all datasets
G
**
Neutrophils [% of leukocytes]
0 20 40 60 80
N T
H
counts per platform (Epithelial cells)
total_counts
100 1,000 10,000 100,000 1,000,000 10,000,000 100,000,000
Smart-seq2
BD-Rhapsody
10x
Singleron
InDrop
DropSeq
I
Ciliated
Alveolar cell type 1
Club
transitional club/AT2
Tumor cells
Alveolar cell type 2
Stromal
T cell CD4
T cell regulatory
Plasma cell
Endothelial cell
Neutrophils
T cell CD8
Macrophage alveolar
pDC
Macrophage
Mast cell
NK cell
cDC2
cDC1
DC mature
Monocyte
B cell
J
T cell CD8
activated
effector memory
naive
terminally exhausted
dividing
NK-like
K
tumor_stage
advanced
early
log2-fold change
−1.0 −0.8 −0.6 −0.4 −0.2 0.0 0.2 0.4 0.6 0.8 1.0 1.2
cDC2

Figure S1: related to Figure 1. Composition of the NSCLC single-cell atlas.

(A) Dotplot of cell type marker genes used for cell-type annotation.

(B) Fractions of cell types, sample origins and conditions per study (extended atlas).

(C) Relative cell type proportions by tissue origin in the core atlas. The depicted fractions are the average of all patients, independent of their cell-count.

(D) Marker genes for cancer cell classification.

(E) Cell type fractions in the extended atlas.

(F) Mean neutrophil fraction per sequencing platform across all datasets.

(G) Flow-cytometry of neutrophils (shown as percentage of leucocytes) in tumor tissue and patient-matched normal-adjacent tissue (n=63; Paired Wilcoxon test, **p<0.01). The horizontal line represents the median, whiskers extend to the inter-quartile range.

(H) Number of reads (Smart-seq2) or UMIs (other platforms) in epithelial cells by sequencing platform. The central line denotes the median, boxes represent the interquartile range (IQR) and whiskers extend to the most extreme values within 1.5 * IQR. Points outside 1.5 * IQR are shown as outliers.

(I) UMAP of the extended atlas, colored by the medium resolution cell-type annotation used for most data analyses.

(J) UMAP of $CD8^{+}$ T cell subclusters according to gene signatures published by Oliveira et al.[22].

(K) Cell type composition by tumor stage (early, late) calculated with scCODA (Bayesian model for differential composition analysis) using cancer cells as reference cell-type (assumed to be constant between conditions), including tumor type (LUAD, LUSC) as a covariate and running 500,000 Markov-chain monte carlo iterations. FDR=0.1.

Table S2: related to Figure 1. Patient number per cell type.

	Number of patients with ≥ 5/ ≥10 / ≥ 30 cells		
**Cell type**	**5**	**10**	**30**
Alveolar cell type 1	133	113	69
Alveolar cell type 2	172	161	122
B cell	226	196	164
B cell dividing	26	16	6
cDC1	131	99	32
cDC2	256	231	172
Ciliated	175	140	87
Club	101	54	21
DC mature	133	79	21
Endothelial cell arterial	108	65	33
Endothelial cell capillary	118	93	54
Endothelial cell lymphatic	129	90	37
Endothelial cell venous	207	171	96
Fibroblast adventitial	123	80	33
Fibroblast alveolar	107	75	38
Fibroblast peribronchial	115	78	38
Macrophage	291	273	242
Macrophage alveolar	228	201	177
Mast cell	205	178	120
Mesothelial	31	18	2
Monocyte classical	276	257	222
Monocyte non-classical	149	120	74
Myeloid dividing	195	153	76
Neutrophils	60	48	35
NK cell	238	219	186
NK cell dividing	71	28	3
pDC	151	113	55
Pericyte	83	52	22
Plasma cell	218	186	131
Plasma cell dividing	44	26	6
ROS1+ healthy epithelial	38	29	16
Smooth muscle cell	85	48	25
Stromal dividing	14	9	3
T cell CD4	269	253	209
T cell CD4 dividing	141	99	32
T cell CD8 activated	144	122	76
T cell CD8 dividing	109	71	27
T cell CD8 effector memory	252	223	193
T cell CD8 naive	183	156	115
T cell CD8 terminally exhausted	166	131	89
T cell NK-like	192	163	125
T cell regulatory	216	192	150
Transitional club/AT2	193	159	106

	Number of patients with ≥ 5/ ≥10 / ≥ 30 cells		
**Cell type**	**5**	**10**	**30**
Tumor cells LUAD	225	192	136
Tumor cells LUAD EMT	43	26	13
Tumor cells LUAD mitotic	98	64	23
Tumor cells LUAD MSLN	6	3	1
Tumor cells LUAD NE	6	3	3
Tumor cells LUSC	86	62	36
Tumor cells LUSC mitotic	79	60	32
Tumor cells NSCLC mixed	24	19	12

Table S3: related to Figure 1. Sample disposition.

Figure	Dataset	Sample origin
1A	overview	
1B	core atlas	all
1C	extended atlas	all
1D	UKIM-V	all
1E	extended atlas	all
1F	extended atlas	primary tumor
1G	independent Lübeck cohort	
2A	extended atlas	primary tumor
2B	extended atlas (cancer cells)	primary tumor
2C	extended atlas (cancer cells)	primary tumor
3A	extended atlas	primary tumor
3B	extended atlas	primary tumor
4A-E	extended atlas	primary tumor
4F	TCGA LUAD/LUSC	
4G	extended atlas (CD8+ T cells)	primary tumor
5A-B	extended atlas (neutrophils)	all
5C	independent FACS cohort	
5D	extended atlas (neutrophils)	all
5E	extended atlas	primary tumor
5F	extended atlas (neutrophils)	all
5G	independent FACS cohort	
5H	representative imaging of UKIM-V patient	
6A-B	extended atlas (neutrophils)	all
6C	independent FACS cohort	
6D-E	UKIM-V (neutrophils)	all
6F	ligands: extended atlas (neutrophils); receptors: extended atlas	ligands: all; receptors: primary tumor
6G	extended atlas	all
6H	extended atlas (neutrophils)	all
6I-K	POPLAR/OAK cohort	
S1A	core atlas	all
S1B	extended atlas	all
S1C	extended atlas	primary tumor + adjacent normal
S1D	core atlas (cancer cells)	primary tumor
S1E	extended atlas	all
S1F	extended atlas	all
S1G	independent FACS cohort	
S1H	extended atlas	all
S1I	extended atlas	all
S1J	extended atlas (CD8+ T cells)	all
S1K	extended atlas	primary tumor
S2A	independent Lübeck cohort	

Figure	Dataset	Sample origin
S2B-C	independent FACS cohort	
S2D	extended atlas	primary tumor
S2E	extended atlas	primary tumor
S3A	extended atlas	primary tumor
S4A-B	extended atlas	primary tumor
S4C	independent IF cohort	
S4E-F	extended atlas	primary tumor
S4G-H	TCGA LUAD/LUSC	
S5A	extended atlas (neutrophils)	primary tumor
S5B	extended atlas (neutrophils)	all
S5C	representative imaging of UKIM-V patient	
S6A-E	extended atlas (neutrophils)	all
S6F	independent FACS cohort	
S6G-K	POPLAR/OAK cohort	
S7A	gating strategy	

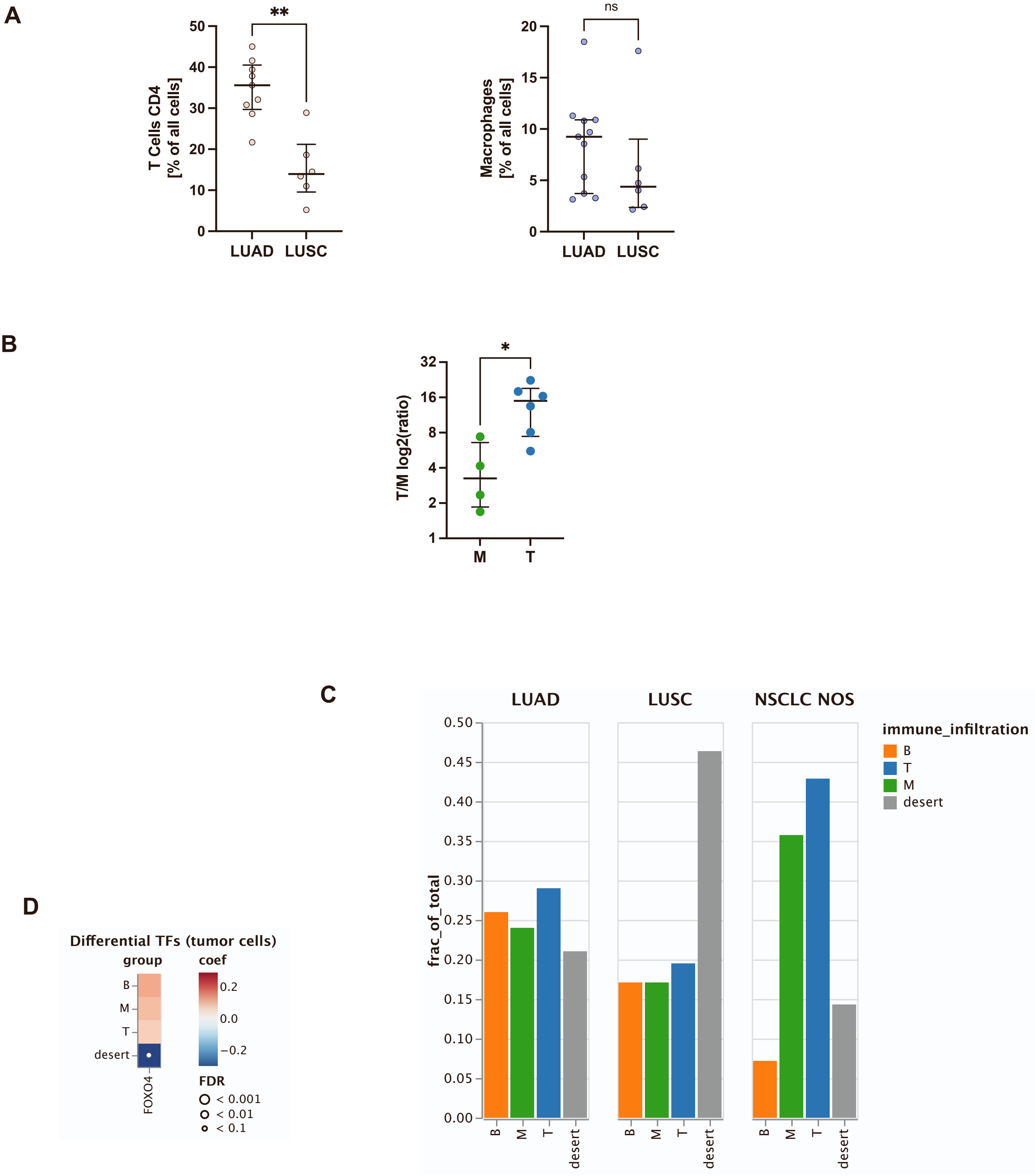
A
**
T Cells CD4
[% of all cells]
50
40
30
20
10
0
LUAD
LUSC
ns
Macrophages
[% of all cells]
20
15
10
5
0
LUAD
LUSC
B
*
T/M log2(ratio)
32
16
8
4
2
1
M
T
C
LUAD
LUSC
NSCLC NOS
frac_of_total
0.50
0.45
0.40
0.35
0.30
0.25
0.20
0.15
0.10
0.05
0.00
B
M
T
desert
immune_infiltration
B
T
M
desert
D
Differential TFs (tumor cells)
group
coef
B
M
T
desert
FOXO4
0.2
0.0
−0.2
FDR
< 0.001
< 0.01
< 0.1

Figure S2: related to Figure 2. Immune phenotypes in histological NSCLC subtypes.

(A) Flow cytometry analysis of $CD4^+$ lymphocytes and macrophages as percentage of all cells in LUAD (n=9) *versus* LUSC (n=6) tumor samples. The horizontal line represents the median, whiskers extend to the inter-quartile range (Wilcoxon test, **p<0.01).

(B) Flow cytometry analysis of the T cell-to myeloid cell ratio (T/M ratio) in tumor tissue (T subtype n=6, M subtype n=4). The horizontal line represents the median, whiskers extend to the inter-quartile range (Wilcoxon test, *p<0.05).

(C) Fractions of immune phenotypes in histological subtypes.

(D) Differential of DoRothEA transcription factor signatures in cancer cells between the four immune phenotypes. Heatmap colors indicate the deviation from the overall mean, independent of tumor histology and stage. White dots indicate significant interactions at different false-discovery-rate (FDR) thresholds. P-values have been calculated using a linear model f-test. Only transcription factors with an FDR < 0.1 in at least one patient group are shown.

A

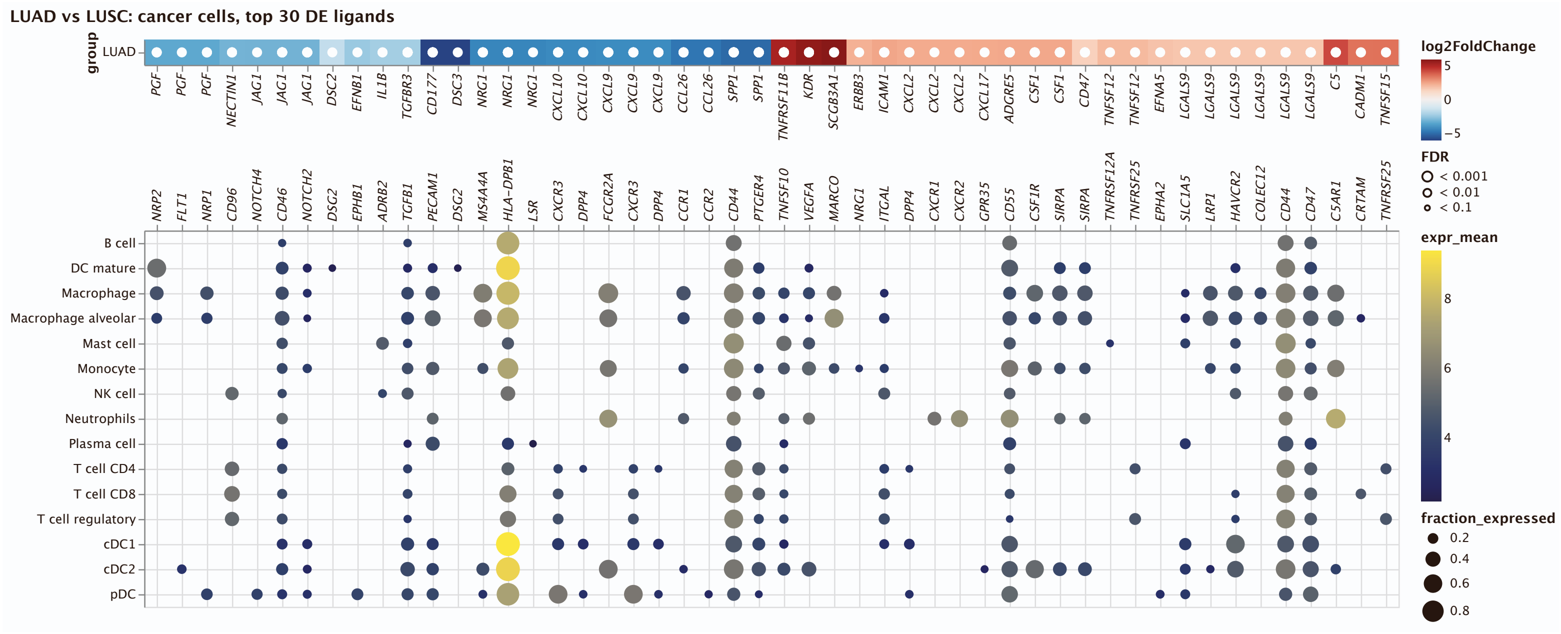

Figure S3: **related to Figure 3. Tumor-immune crosstalk in LUAD *vs.* LUSC,**

(A) Upper panel: top 30 differentially expressed ligands in LUAD *vs.* LUSC (DESeq2 on pseudo-bulk, FDR < 0.01). Heatmap colors indicate log2 fold changes clipped at ±5, where blue indicates upgregulation in LUSC and red indicates upregulation in LUAD. Bottom panel: Respective receptors and the expression by cell type. Dot sizes and colors refers to the fraction of cells expressing the receptor and gene expression, respectively, averaged over all patients. Dots are only shown for receptors that are expressed in at least 10% of the respective cell-types.

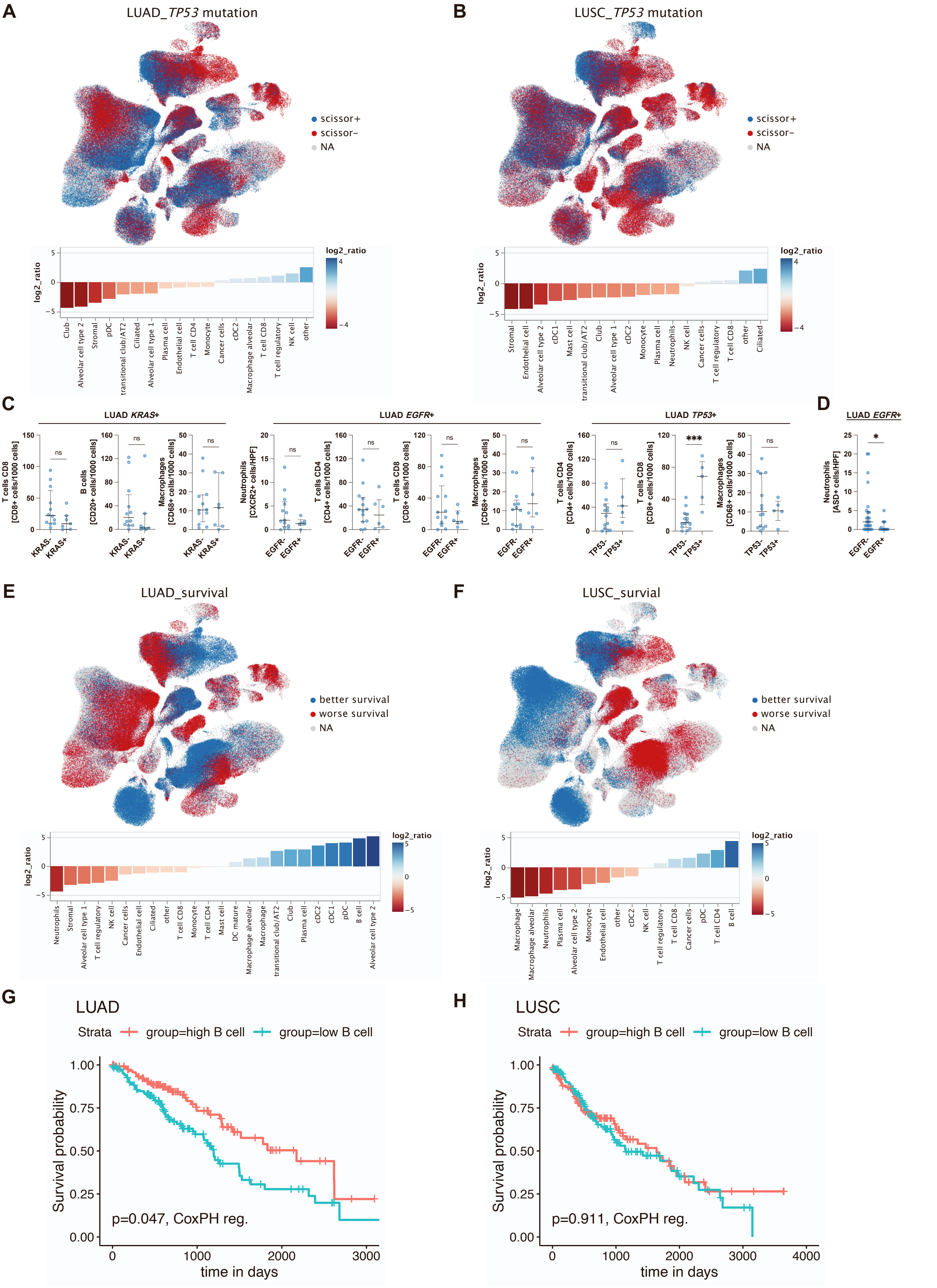
A
LUAD_TP53 mutation
scissor+
scissor−
NA
log2_ratio
B
LUSC_TP53 mutation
C
LUAD KRAS+
LUAD EGFR+
LUAD TP53+
D
LUAD EGFR+
E
LUAD_survival
better survival
worse survival
F
LUSC_survial
G
LUAD
Strata
group=high B cell
group=low B cell
Survival probability
time in days
p=0.047, CoxPH reg.
H
LUSC
p=0.911, CoxPH reg.

Figure S4: related to Figure 4. Association of cellular composition and distinct genotypes and survival in the TCGA data,

(A-B) *SCISSOR* analysis showing the association of cellular composition and *TP53* mutation in LUAD and LUSC derived from the TCGA reference dataset.

(C) Staining of tumor tissue from LUAD patients with *KRAS* (n=7), *EGFR* (n=6) or *TP53* (n=5) mutation compared to LUAD tumor tissue negative for the respective mutation (n=12). Multiplex immunofluoresence was performed to detect $CD4^+$ T cells, $CD8^+$ T cells, $CD20^+$ B cells, or $CD68^+$ macrophages, respectively. Positive stained cells per 1000 cells are given. *CXCR2* expression was analyzed by immunohistochemistry and quantified per high power field in patients with *EGFR* mutation compared to EGFR wt patients. Analyses related to the corresponding *SCISSOR* analyses are shown. The horizontal line represents the median, whiskers extend to the inter-quartile range (Wilcoxon test, ***p<0.001).

(D) Immunohistochemistry staining of neutrophils (ASD^+ cells) quantified per high power field in patients with *EGFR* mutation (n=11) compared to *EGFR* wt patients (n=26). The horizontal line represents the median, whiskers extend to the inter-quartile range (Wilcoxon test, *p<0.05).

(E-F) Association of cellular composition with overall survival for LUSC and LUAD patients.

(G-H) Kaplan-Meyer plot of LUAD and LUSC patients with high (top 25%) and low (bottom 25%) B cell fractions of TCGA lung cancer patients as determined by deconvolution with EPIC. P-value has been determined using CoxPH-regression using tumor stage and age as covariates.

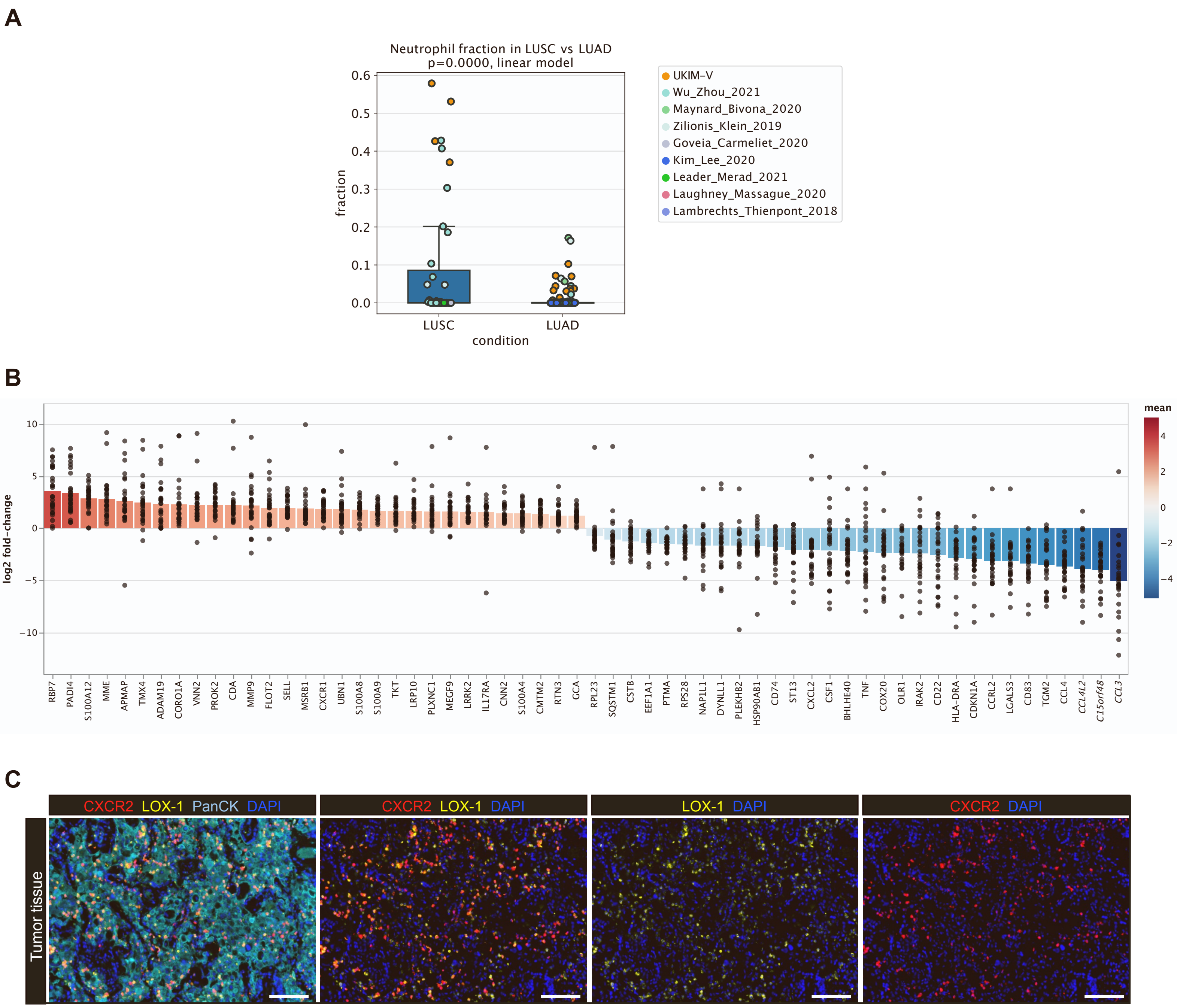

Figure S5: related to Figure 5. Characterization of tissue-resident neutrophils.

(A) Neutrophil fraction in LUSC *vs.* LUAD (extended atlas). P-value derived using linear model f-test including dataset as a covariate.

(B) Expression of top 30 marker genes (AUROC > 0.75) for NANs and TANs. Every dot is the log2 fold change on a single patient. Bars show the average log2-fold change.

(C) Multiplex immunofluorescence co-staining of CXCR2 (red), LOX-1 (yellow) and pan-cytokeratin (blue) in LUSC tumor tissue. Scale bar = 100 µm.

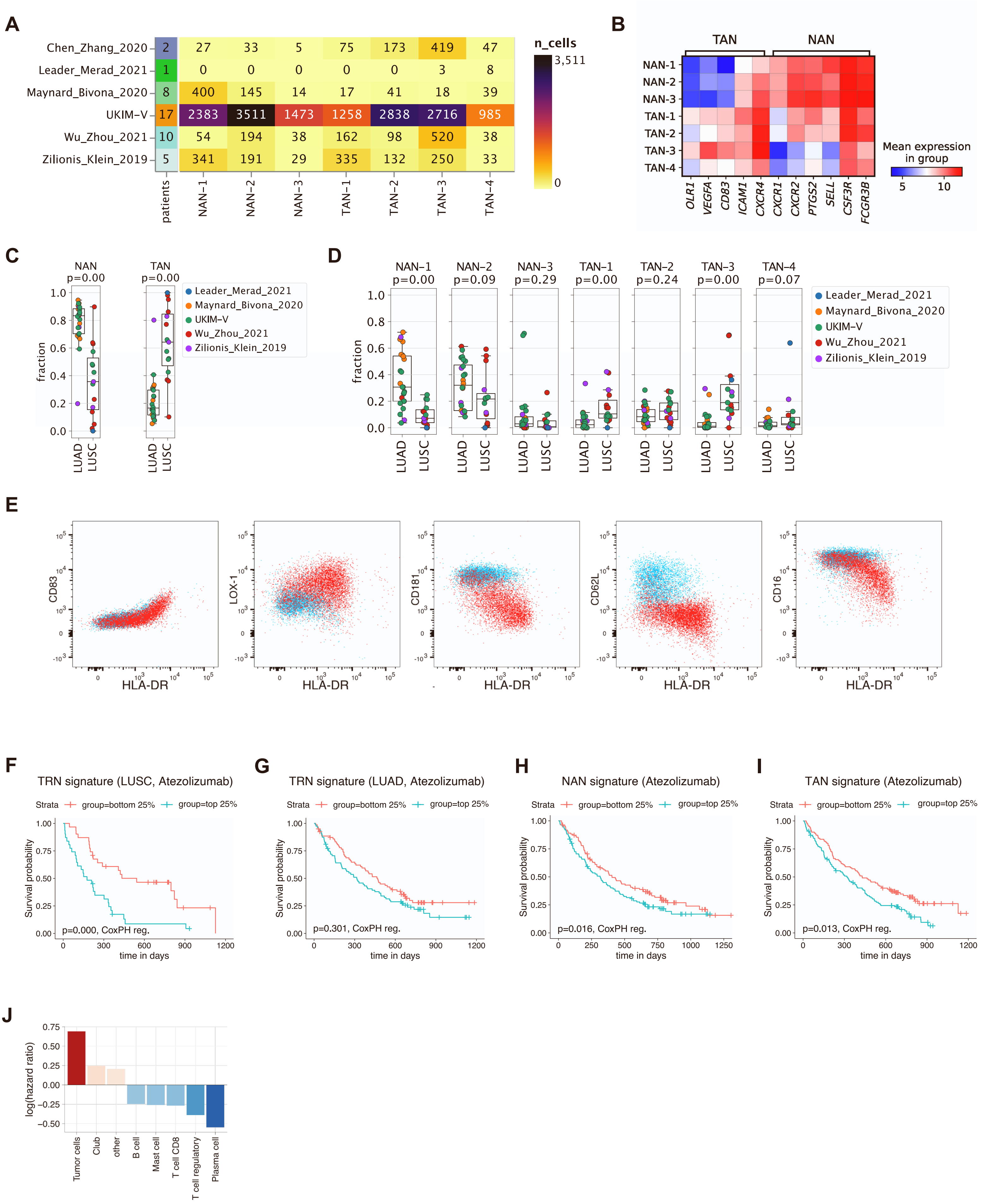
A
Chen_Zhang_2020
Leader_Merad_2021
Maynard_Bivona_2020
UKIM-V
Wu_Zhou_2021
Zilionis_Klein_2019
patients
NAN-1
NAN-2
NAN-3
TAN-1
TAN-2
TAN-3
TAN-4
2 27 33 5 75 173 419 47
1 0 0 0 0 0 3 8
8 400 145 14 17 41 18 39
17 2383 3511 1473 1258 2838 2716 985
10 54 194 38 162 98 520 38
5 341 191 29 335 132 250 33
n_cells
3,511
0
B
TAN
NAN
NAN-1
NAN-2
NAN-3
TAN-1
TAN-2
TAN-3
TAN-4
OLR1
VEGFA
CD83
ICAM1
CXCR4
CXCR1
CXCR2
PTGS2
SELL
CSF3R
FCGR3B
Mean expression in group
5
10
C
NAN p=0.00
TAN p=0.00
fraction
LUAD
LUSC
Leader_Merad_2021
Maynard_Bivona_2020
UKIM-V
Wu_Zhou_2021
Zilionis_Klein_2019
D
NAN-1 p=0.00
NAN-2 p=0.09
NAN-3 p=0.29
TAN-1 p=0.00
TAN-2 p=0.24
TAN-3 p=0.00
TAN-4 p=0.07
fraction
LUAD
LUSC
E
CD83
LOX-1
CD181
CD62L
CD16
HLA-DR
F
TRN signature (LUSC, Atezolizumab)
Strata group=bottom 25% group=top 25%
Survival probability
p=0.000, CoxPH reg.
time in days
G
TRN signature (LUAD, Atezolizumab)
p=0.301, CoxPH reg.
H
NAN signature (Atezolizumab)
p=0.016, CoxPH reg.
I
TAN signature (Atezolizumab)
p=0.013, CoxPH reg.
J
log(hazard ratio)
Tumor cells
Club
other
B cell
Mast cell
T cell CD8
T cell regulatory
Plasma cell

Figure S6: related to Figure 6. Tissue-resident neutrophil subtypes in NSCLC.

(A) TRN subclusters by the contributing datasets. Left column shows the number of patients with >10 neutrophils in the respective study, the heatmap depicts the number of cells per neutrophil cluster. Data of 43 patients with each > 10 neutrophils.

(B) TAN/NAN candidate marker gene expression by neutrophil subclusters. Expression values are the mean across pseudobulk samples by patient.

(C) NAN and TAN fractions in LUAD *versus* LUSC. Each dot refers to a patient with at least 10 neutrophils. P-values are derived from a t-test and not adjusted for multiple testing. In all boxplots, the central line denotes the median. Boxes represent the interquartile range (IQR) of the data, whiskers extend to the most extreme data points within 1.5 times the IQR.

(D) Neutrophils subcluster fractions in LUAD versus LUSC. Each dot refers to a patient with at least 10 neutrophils. P-values are derived from a t-test and not adjusted for multiple testing. In all boxplots, the central line denotes the median. Boxes represent the interquartile range (IQR) of the data, whiskers extend to the most extreme data points within 1.5 times the IQR.

(E) Flow cytometry analysis demonstrating the correlation between HLA-DR expression and CD83, LOX-1, CD181, CD62L or CD16 expression, respectively. Representative analysis of neutrophils derived from NSCLC normal-adjacent tissue (blue) and tumor tissue (red) are shown.

(F) Kaplan-Meyer plot of LUSC patients form the POPLAR (Fehrenbacher et al., 2016) and OAK (Rittmeyer et al., 2017) cohorts treated with atezolizumab with high (top 25%) and low (bottom 25%) TRN signature score. P-value has been determined using CoxPH-regression.

(G) Kaplan-Meyer plot of LUAD patients form the POPLAR (Fehrenbacher et al., 2016) and OAK (Rittmeyer et al., 2017) cohorts treated with atezolizumab with high (top 25%) and low (bottom 25%) TRN signature score. P-value has been determined using CoxPH-regression.

(H) Kaplan-Meyer plot comparing patients treated with atezolizumab with high (top 25%) and low (bottom 25%) NAN signature scores. P-value has been determined using CoxPH-regression including cohort and histology as covariates.

(I) Kaplan-Meyer plot comparing patients treated with atezolizumab with high (top 25%) and low (bottom 25%) TAN signature scores. P-value has been determined using CoxPH-regression including cohort and histology as covariates.

(J) Predictive value of cell-type signatures in bulk RNA-seq data from the OAK[80] and POPLAR[79] cohorts of NSCLC patients treated with atezolizumab (anti-PD-L1). The bar charts show cell-type signatures that are associated with worse (log hazard ratio > 0) or better (log hazard ratio < 0) survival at an FDR < 0.1. The hazard ratio and p-values have been determined using CoxPH regression including cohort and histology as covariates.

Table S4: related to Figure 6. Genes signatures.

TRN signature (n=38)	NAN signature (n=20)	TAN signature (n=18)
AGO4	AGO4	CCR3
ARG1	ARG1	CCRL2
CCR3	CYP4F3	DDIT3
CCRL2	ERGIC1	FLOT1
CYP4F3	FLOT2	HIF1A
DDIT3	FRAT2	IRAK2
ERGIC1	LRP10	MAFF
FLOT1	MGAM	MAP1LC3B2
FLOT2	MMP25	MCOLN1
FRAT2	MSRB1	NBN
HIF1A	NDEL1	NOD2
IRAK2	NFE2	PI3
LRP10	PADI4	PLAU
MAFF	PBX2	PPIF
MAP1LC3B2	PHOSPHO1	TGM3
MCOLN1	RASGRP4	TOM1
MGAM	REPS2	UBR5-AS1
MMP25	SULT1B1	ZNF267
MSRB1	TSEN34	
NBN	XKR8	
NDEL1		
NFE2		
NOD2		
PADI4		
PBX2		
PHOSPHO1		
PI3		
PLAU		
PPIF		
RASGRP4		
REPS2		
SULT1B1		
TGM3		
TOM1		
TSEN34		
UBR5-AS1		
XKR8		
ZNF267		

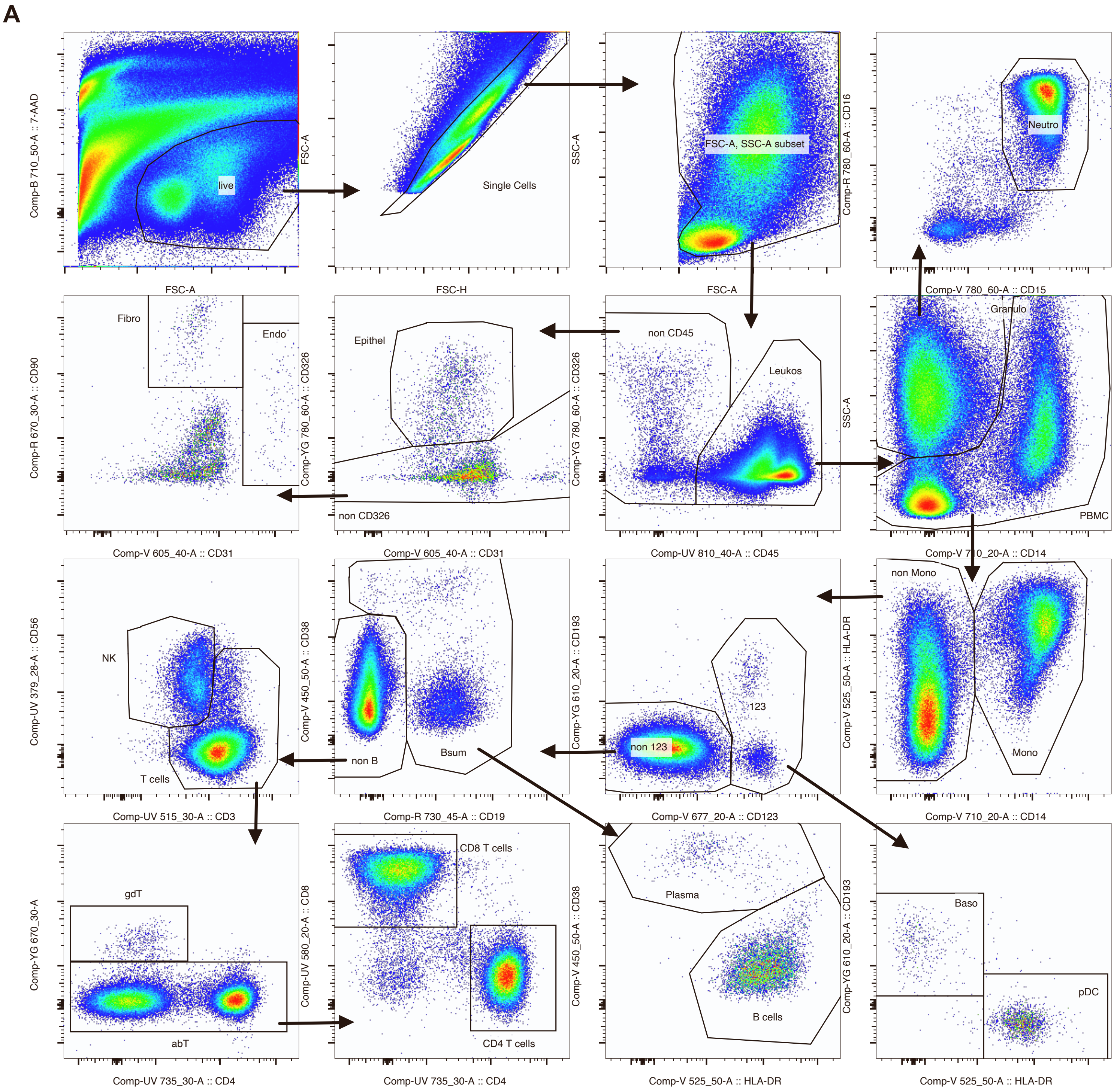

Figure S7 related to STAR Methods. Flow cytometry gating strategy to define cell populations from NSCLC tumor tissue and normal adjacent tissue.

(A) In initial cleaning steps dead cells, debris and doublets were removed using 7-AAD staining and scatter characteristics. Leukocytes were defined by CD45 staining and sequentially gated into subtypes including neutrophils, monocytes, T cells and B cells. Non-CD45^{+} cells were gated into epithelial cells, endothelial cells and fibroblasts.

Table S7: related to STAR Methods. Overview of antibody information, imultispectral imaging, and mmunohistochemistry.

Antibody cocktails used for flow cytometry:

Backbone		Cell definition		Neutrophil Characterization	
**Antigen**	**Fluorochrome**	**Antigen**	**Fluorochrome**	**Antigen**	**Fluorochrome**
CD56	BUV395	CD28	BUV615P	CD54	FITC
CD3	BUV496	CD38	BV421	CD83	FITC
CD8	BUV563	CD123	BV650	CD49b	PE
CD4	BUV737	CD34	FITC	CD62L	PE
CD45	BUV805	CD161	PE	LOX-1	PE
HLA-DR	BV480	CD193	PE-CF594	CD181	APC
CD31	BV605	TCRgd	PE-Cy5		
CD14	BV711	CD90	APC		
CD15	BV786				
CD326	PE-Cy7				
CD19	APC-R700				
CD16	APC-eF780				

Antibody-antigen retrieval and opal fluorophore pairing related to multispectral imaging:

Antibody	pH (AR)	Opal Pairing	Clone	Provider	Dilution
CXCR2	9	570	EPR22301-103	Abcam	1:500
LOX-1	9	540	polyclonal	Sigma-Aldrich	1:200
CD16	9	650	EPR22409-124	Abcam	1:600
CD8	9	570	C8\144B	Dako/Agilent	1:200
CD3	6	620	polyclonal	Dako/Agilent	1:250
CD68	9	650	PG-M1	Dako/Agilent	1:200
CD20	6	540	L26	Dako/Agilent	1:200
DAPI	7.4	450	-	Akoya Biosciences	1:15
Cytokeratin	9	450	AE1/AE3	Dako/Agilent	1:500
Cytokeratin	9	690	C-11	Abcam	1:1000

Overview of antibodies used for immunohistochemistry:

Antibody	Clone	Provider	Dilution
CD4	SP35	Ventana	pre-diluted
CD68	KP1	Ventana	pre-diluted
CXCR2	EPR22301-103	Abcam	1:500

Table S8: related to STAR methods. Quality control thresholds related to datasets integrated into the NSCLC single-cell atlas.

Dataset	min counts	max counts	min genes	max genes	max pct_mito
Adams_Kaminski_2020_COPD	1000	35000	500	10000	20
Chen_Zhang_2020_NSCLC	600	30000	250	10000	20

Goveia_Carmeliet_2020_NSCLC	600	30000	250	10000	20
Guo_Zhang_2018_NSCLC	20000	3000000	1000	20000	20
Habermann_Kropski_2020_pulmonary-fibrosis	600	30000	200	10000	20
Kim_Lee_2020_LUAD	1000	35000	300	10000	20
He_Fan_2021_LUAD	600	30000	250	10000	20
Lambrechts_2018_LUAD_6149v1	600	30000	200	10000	15
Lambrechts_2018_LUAD_6149v2	600	30000	250	10000	20
Lambrechts_2018_LUAD_6653	1200	40000	250	10000	20
Laughney_Massague_2020_NSCLC	1800	40000	500	10000	20
Madissoon_Meyer_2020_pulmonary-fibrosis	600	30000	300	10000	20
Maier_Merad_2020_NSCLC	1000	30000	400	10000	15
Maynard_Bivona_2020_NSCLC	20000	20000000	600	20000	30
Mayr_Schiller_2020_pulmonary-fibrosis	600	30000	250	10000	10
Reyfman_Misharin_2018_pulmonary-fibrosis	1000	30000	250	10000	20
Travaglini_Krasnow_2020_Lung_10x	1000	30000	500	10000	0
Travaglini_Krasnow_2020_Lung_SS2	20000	6000000	600	20000	30
UKIM-V	2000	100000	200	8000	30
Vieira_Teichmann_2019_asthma	600	30000	200	10000	20
Wu_Zhou_2021_NSCLC	600	30000	300	10000	30
Zilionis_Klein_2019_NSCLC	600	30000	200	10000	20
UKIM-V-2	1000	60000	200	8000	30
Leader_Merad_2021_10x_3p_v1_sort	600	30000	220	10000	10
Leader_Merad_2021_10x_3p_v2_beads_cite	600	30000	300	10000	25
Leader_Merad_2021_10x_3p_v2_beads	1000	30000	500	10000	20
Leader_Merad_2021_10x_3p_v2_digest-deadcell_cite	1000	30000	500	10000	20
Leader_Merad_2021_10x_3p_v2_sort	600	30000	250	10000	25
Leader_Merad_2021_10x_3p_v3_beads	600	30000	250	10000	30
Leader_Merad_2021_10x_5p_v1_beads	1100	30000	500	10000	25
Leader_Merad_2021_10x_5p_v1_CD2	1100	30000	500	10000	15
